# Activation of Ca^2+^ phosphatase Calcineurin regulates Parkin translocation to mitochondria and mitophagy in flies

**DOI:** 10.1038/s41418-023-01251-9

**Published:** 2024-01-18

**Authors:** Elena Marchesan, Alice Nardin, Sofia Mauri, Greta Bernardo, Vivek Chander, Simone Di Paola, Monica Chinellato, Sophia von Stockum, Joy Chakraborty, Stephanie Herkenne, Valentina Basso, Emilie Schrepfer, Oriano Marin, Laura Cendron, Diego L. Medina, Luca Scorrano, Elena Ziviani

**Affiliations:** 1 Department of Biology, University of Padova, Padova, Italy; 2 Telethon Institute of Genetics and Medicine (TIGEM), Pozzuoli, Naples Italy; 3 Dulbecco-Telethon Institute, Venetian Institute of Molecular Medicine (VIMM), Padova, Italy; 4 Department of Biomedical Sciences (DSB), University of Padova, Padova, Italy; 5 Medical Genetics Unit, Department of Medical and Translational Science, Federico II University, Naples, Italy; 6 Present Address: Institute for Experimental Endocrinology and Oncology (IEOS), National Research Council (CNR), Napoli, Italy

**Keywords:** Macroautophagy, Neurological disorders

## Abstract

Selective removal of dysfunctional mitochondria via autophagy is crucial for the maintenance of cellular homeostasis. This event is initiated by the translocation of the E3 ubiquitin ligase Parkin to damaged mitochondria, and it requires the Serine/Threonine-protein kinase PINK1. In a coordinated set of events, PINK1 operates upstream of Parkin in a linear pathway that leads to the phosphorylation of Parkin, Ubiquitin, and Parkin mitochondrial substrates, to promote ubiquitination of outer mitochondrial membrane proteins. Ubiquitin-decorated mitochondria are selectively recruiting autophagy receptors, which are required to terminate the organelle via autophagy. In this work, we show a previously uncharacterized molecular pathway that correlates the activation of the Ca^2+^-dependent phosphatase Calcineurin to Parkin translocation and Parkin-dependent mitophagy. Calcineurin downregulation or genetic inhibition prevents Parkin translocation to CCCP-treated mitochondria and impairs stress-induced mitophagy, whereas Calcineurin activation promotes Parkin mitochondrial recruitment and basal mitophagy. Calcineurin interacts with Parkin, and promotes Parkin translocation in the absence of PINK1, but requires PINK1 expression to execute mitophagy in MEF cells. Genetic activation of Calcineurin in vivo boosts basal mitophagy in neurons and corrects locomotor dysfunction and mitochondrial respiratory defects of a *Drosophila* model of impaired mitochondrial functions. Our study identifies Calcineurin as a novel key player in the regulation of Parkin translocation and mitophagy.

## Introduction

The Ubiquitin Proteasome System (UPS) and mitophagy are dysregulated in many neurological diseases, including Parkinson’s Disease (PD), a neurodegenerative condition characterized by dopaminergic neuron loss [1, 2], accumulation of ubiquitinated unfolded protein aggregates [3, 4], mitochondrial dysfunction and mitophagy dysregulation [5, 6]. Although most PD cases are sporadic, a small proportion derives from mutations in PD-associated genes [7, 8], which have been identified by characterizing familiar Mendelian inherited PD forms, and include mutations in the mitophagy genes PINK1 and Parkin [9–11]. Parkin belongs to the RBR (RING-between-RING) type of E3 ubiquitin ligases [12], also known as RING/HECT hybrids, consisting of a ubiquitin-like domain (Ubl), followed by two RING fingers domains (RING0 and RING1), an in-between RING finger domain (IBR), a linker domain called Repressor Element of Parkin (REP) and a third RING finger domain called RING2 [13–16]. Under basal conditions, Parkin activity is repressed and the protein maintains a close structure with the Ubl domain and the REP fragment occluding the RING1 domain, and the RING0 domain impeding on the catalytic site-containing RING2 domain [14–16]. The gene product has a number of neuroprotective roles and pleiotropic functions and its activation is involved in many different survival pathways [17–19], including those that are affecting mitochondrial function by regulating mitochondria quality control [20–24]. How Parkin controls so many different cellular processes is under intense investigation. However, by being a versatile E3 ubiquitin ligase that both promotes degradative Lys 48-mediated ubiquitination and nonclassical, proteosomal-independent ubiquitination [25–31], Parkin has the potential of controlling a broad subset of cellular processes. Not surprisingly, Parkin activity is repressed under basal conditions, and its activation is tightly regulated by a number of molecular processes, which are largely mediated by post-translational modifications [13, 14, 16].

Parkin function is closely related to the activity of another PD-related gene, PARK6, which encodes for a protein called PINK1 [32–34]. PINK1 is a Serine/Threonine kinase that is imported into mitochondria, where it gets cleaved by the inner membrane protease PARL and then eliminated by the proteasome [35–37]. On depolarized mitochondria, PINK1 accumulates on the outer mitochondrial membrane (OMM), where it promotes Parkin translocation and mitochondrial recruitment [32–34, 38]. Independent studies showed that PINK1-mediated phosphorylation of Parkin and Ubiquitin at residue Serine 65 (Ser65) is required for Parkin translocation to defective mitochondria, and for its E3-ubiquitin ligase activity [39–42]. This process leads to Parkin-dependent ubiquitination and proteasomal degradation of OMM proteins, and to the selective autophagy of damaged mitochondria [43–47]. PINK1 phosphorylates a number of Parkin substrates including the pro-fusion protein Mitofusin, Mfn2, which work as Parkin receptor [48]. It was proposed that Parkin-dependent ubiquitination of Mitofusins prevents mitochondrial fusion of dysfunctional mitochondria by promoting proteasomal degradation of Mfn1 and Mfn2 [24], and segregates depolarized mitochondria from the mitochondrial network, impairing their ability to refuse [49]. In addition, PINK1 directly promotes mitochondrial fission by regulating the recruitment and activation of pro-fission protein Drp1 [50]. In resting conditions, Drp1 is phospho-inhibited at Ser 637 by AKAP1-PKA (A-kinase anchoring protein complex and protein kinase A, respectively) [51]. Following mitochondria damage, PINK1 becomes active and disrupts the AKAP1-PKA complex to promote Drp1-dependent fission [50]. Moreover, PINK1 directly phosphorylates Drp1 on Ser 616 to regulate mitochondrial fission [52]. Interestingly, Drp1 is selectively recruited to dysfunctional mitochondria in the proximity of PINK1/Parkin suggesting that mitochondrial division occurs at sites where the PINK1/Parkin-dependent mitochondrial clearance program is initiated [53]. Expression of dominant-negative Drp1 to inhibit mitochondrial fission prevents mitophagy, indicating the importance of fission in mitophagy [54], which is also supported by studies on yeasts [55]. Translocation of Drp1 to mitochondria is also mediated by selective dephosphorylation of residue Serine 637, which is controlled by Calcium (Ca^2+^) and Ca^2+^/Calmodulin dependent phosphatase Calcineurin (CaN) [51]. Rise of cytosolic Ca^2+^ associated to mitochondrial depolarization [56], leads to CaN-dependent Drp1 dephosphorylation and Drp1 mitochondrial recruitment to promote mitochondrial fission [51]. CaN also dephosphorylates transcription factor TFEB to promote its nuclear translocation and the expression of autophagy and lysosomal genes, thus actively contributing to autophagy and lysosomal biogenesis [57], and the proteostatic activity of the cell.

Mitochondrial fragmentation, which is required to execute mitophagy [58, 59], is not the only important requisite for efficient mitochondrial quality control. Several studies highlighted the importance of mitochondrial trafficking and segregation as a mean to isolate dysfunctional mitochondria from the mitochondrial network, and allow efficient elimination. In this respect, Parkin was found to ubiquitinate the mitochondrial outer membrane Rho GTPases Miro1/2, a Ca^2+^ -binding protein that is involved in the regulation of mitochondrial movement by Ca^2+^. Parkin-dependent ubiquitination of Miro1/2 leads to mitochondrial arrest facilitating the removal of damaged mitochondria via autophagy. Moreover, Miro1 recruits Parkin to dysfunctional mitochondria in a PINK1-independent fashion, functioning as a Ca^2+^-sensitive mitochondrial-docking site for Parkin, independently of PINK1 activation.

Despite the crucial role of Ca^2+^ signalling in cellular homeostasis and mitochondrial function, the involvement of Ca^2+^ and Ca^2+^-regulated proteins in mitochondrial quality control have been poorly explored.

In this work, we show that Ca^2+^/Calmodulin phosphatase CaN is required for Parkin mitochondrial recruitment and mitophagy in CCCP-treated mouse embryonic fibroblast cells (MEFs). CaN activation is sufficient to promote Parkin translocation under basal condition in a PINK1-independent fashion, but requires Miro1 for Parkin mitochondrial recruitment. Moreover, genetic activation of CaN in vivo promotes basal mitophagy in neurons, and corrects locomotor dysfunction and mitochondrial defects of a *Drosophila* model of impaired mitochondrial function. Thus, activation of Ca^2+^-dependent phosphatase CaN enhances basal mitophagy in vivo, and favor survival.

## Results

### Parkin translocation to mitochondria is regulated by Calcineurin

**Fig. 1:**
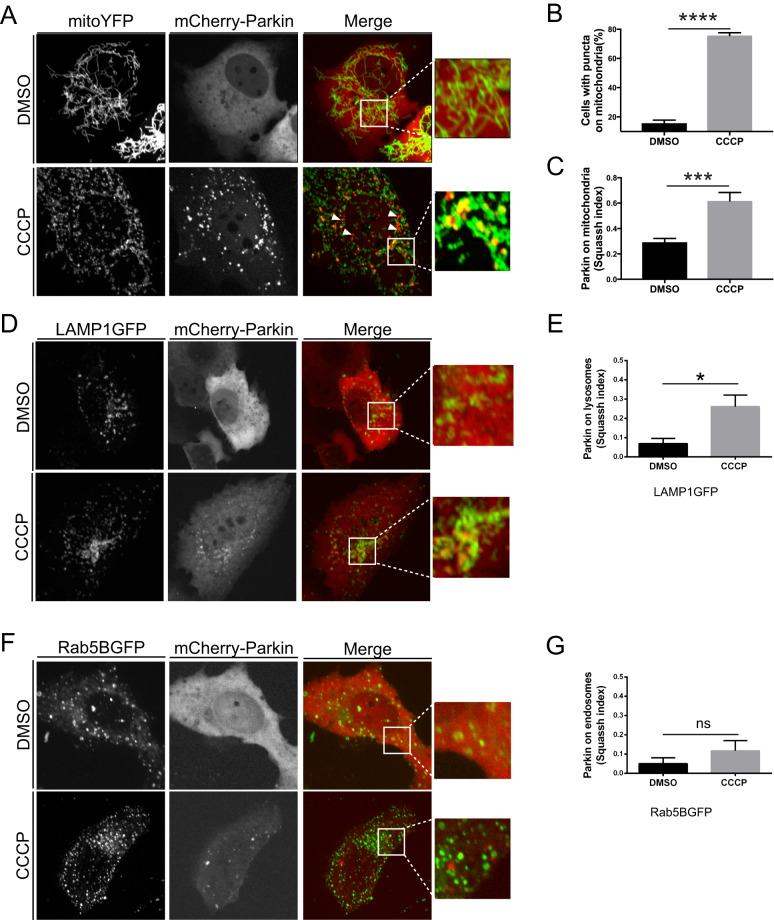
CCCP promotes Parkin translocation to mitochondria and lysosomes. **A** Representative confocal images of MEF cells transfected with mCherry-Parkin and mito-YFP for 2 days before being treated with DMSO or 10 μM CCCP for 3 h. The panels on the right show enlarged merged views of the boxed areas. **B** Quantification of A. Graph bar shows mean ± SEM of percentage of cells with mCherry-Parkin on mitochondria for at least ≥ 300 cells per biological replicate. Student’s t-test (*n* = 9-10; *p* < 0.0001). **C** Quantification of A by using Squassh. The graph bars show mean ± SEM of Squassh colocalization coefficient for at least ≥ 50 images per biological replicate. 0=no colocalization, 1 = perfect colocalization. Student’s t-test (*n* = 5; *p* < 0.0001). **D** Representative confocal images of MEF cells transfected with mCherry-Parkin and LAMP1GFP for 2 days and treated with 10 μM CCCP for 3 h. **E** Quantification of D by using Squassh. The graph bars show mean ± SEM of Squassh colocalization coefficient for at least ≥ 50 images per biological replicate. Student’s t-test (*n* = 3; *p* < 0.05). **F** Representative confocal images of MEF cells transfected with mCherry-Parkin and with Rab5GFP for 2 days and treated with 10 μM CCCP for 3 h. **G** Quantification of F by using Squassh. The graph bars show mean ± SEM of Squassh colocalization coefficient for at least ≥ 50 images per biological replicate. Student’s t-test (*n* = 3; *p* < 0.05).

We transfected mouse embryonic fibroblasts (MEFs), with fluorescent mCherry-Parkin and mitochondrial-targeted YFP (mitoYFP), and analyzed Parkin subcellular localization by confocal microscopy, following established experimental protocols [34, 38, 45]. Consistent with previous studies [33, 38, 45, 60], we observed that mCherry-Parkin was predominantly located in the cytosol in non-treated cells (Fig. 1A, upper panel). Following the treatment with uncoupling agent carbonyl cyanide m-chlorophenylhydrazone (CCCP), a significant proportion of cells (75,7 ± 1,9%) showed mCherry-Parkin accumulated on or near fragmented mitochondria (Fig. 1A, lower panel), forming discrete dots, which we called puncta (quantified in Fig. 1B). An automated analysis of the confocal images with Squassh, an ImageJ plugin that calculates the degree of colocalization between two channels [61], allowed to consolidate this result. In this analysis, the colocalization coefficient computed by the Squassh plugin ranges from 0 to 1, where 0 indicates no colocalization, and 1 perfect colocalization between mCherry-Parkin and YFP-labeled mitochondria. According to this analysis, Squassh index raised from 0,29 ± 0,03 to 0,62 ± 0,06 following CCCP treatment, indicative of increased Parkin mitochondrial recruitment (Fig. 1C). A small proportion of Parkin puncta did not seem to colocalize with mitochondria (Fig. 1A, arrowhead) suggesting a potential recruitment of Parkin to other organelles. To test this hypothesis, we cotransfected mCherry-Parkin expressing MEFs with the lysosomal marker LAMP1GFP or the endosomal marker Rab5BGFP. Colocalization analysis using Squassh demonstrated that in CCCP-treated cells a small proportion of Parkin puncta colocalized with lysosomes (Fig. 1D, quantified in Fig. 1E), whether no significant recruitment to endosomes occurs (Fig. 1F; quantified in Fig. 1G).

**Fig. 2:**
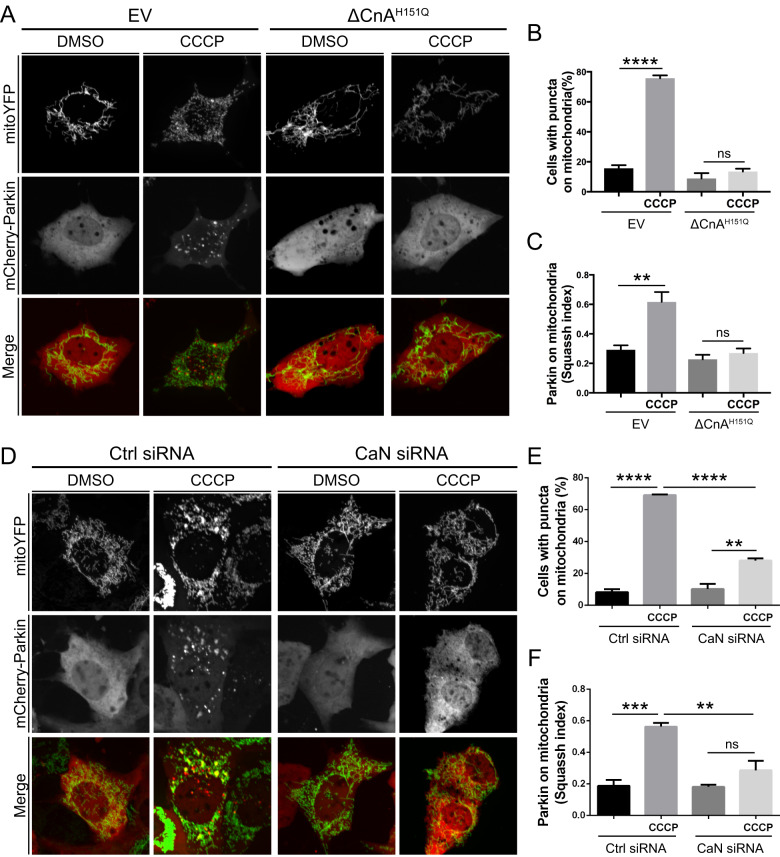
Parkin translocation to mitochondria is regulated by Calcineurin. **A** Representative confocal images of MEF cells transfected with mCherry-Parkin, mito-YFP and dominant negative CaN (ΔCnA^H151Q^) or the empty vector (EV) for 2 days before being treated with DMSO or 10 μM CCCP for 3 h. **B** Quantification of **A**. Graph bar shows mean ± SEM of percentage of cells with mCherry-Parkin on mitochondria for at least ≥ 300 cells per biological replicate. Two-way ANOVA followed by Tukey’s multiple comparison test (*n* = 3-9; *p* < 0.0001). **C** Quantification of **A** using Squassh. The graph bars show mean ± SEM of Squassh colocalization coefficient for at least ≥ 50 images per biological replicate. 0 = no colocalization, 1 = perfect colocalization. Two-way ANOVA followed by Tukey’s multiple comparison test (*n* = 4-5; *p* < 0.001). **D** Representative confocal images of MEF cells transfected with mCherry-Parkin, mito-YFP in which CaN was downregulated, and relative control. **E** Quantification of **D**. Graph bar shows mean ± SEM of percentage of cells with mCherry-Parkin on mitochondria for at least ≥ 300 cells per biological replicate. Two-way ANOVA followed by Tukey’s multiple comparison test (*n* = 3; *p* < 0.01). **F** Quantification of **D** using Squassh. The graph bar shows mean ± SEM of Squassh colocalization coefficient for at least ≥ 50 images per biological replicate. 0=no colocalization, 1 = perfect colocalization. At least 3 independent experiments were performed. Two-way ANOVA followed by Tukey’s multiple comparison test (*n* = 3; *p* < 0.01).

In neuronal cells cytosolic Ca^2+^ rise associated to glutamate-induced excitotoxicity promotes Parkin translocation [62] whereas Ca^2+^ chelation prevents mitophagy [63], indicating a role for Ca^2+^-dependent signaling in the regulation of Parkin mitochondrial recruitment and mitophagy. Indeed, the addition of the protophore CCCP, required for triggering Parkin translocation, induces a transient increase of Ca^2+^ influx [56], and Ca^2+^ chelation with BAPTA completely abolished Parkin translocation (Supplementary Fig. 1). Because mitochondria need to arrest and fragment to be engulfed by the autophagosome [58, 59], and rise of cytosolic Ca^2+^ associated to mitochondrial depolarization leads to mitochondrial arrest [64] and Calcineurin (CaN)-dependent Drp1 fission [51] and autophagy [57], we thought that CaN may also play a role in mitochondrial quality control, and specifically in Parkin recruitment and Parkin-dependent mitophagy. Treatment with FK506, an immunosuppressive agent that blocks CaN without affecting the permeability transition pore (PTP) [65], impaired Parkin translocation (Supplementary Fig. 2). This was also the case upon addition of smaller concentration of CCCP, and following treatment with other mitochondrial damaging compounds that promoted mitochondrial depolarization (Supplementary Fig. 3). To further support this result, we took advantage of the existing dominant negative mutant of CaN (ΔCnA^H151Q^) [66, 67]. CaN is a heterodimer, composed of a catalytic subunit (CnA) that binds calmodulin and a regulatory subunit (CnB) that binds Ca^2+^. Ca^2+^/calmodulin activates CaN upon binding to the calmodulin-binding domain of CnA and inducing the dissociation of the autoinhibitory domain from the catalytic domain [51]. ΔCnA^H151Q^ dominant negative mutant misses the calmodulin-binding domain and the autoinhibitory domain and harbors an inactivating His-151 to Gln point mutation. We cotransfected MEFs with mCherry-Parkin and dominant negative CaN (CnB plus ΔCnA^H151Q^) and looked at Parkin localization. As previously showed, CCCP-induced Parkin mitochondrial recruitment was clearly visible following 3 h CCCP (Fig. 2A, left panel). This event was completely abolished when in the presence of the dominant negative CaN, ΔCnA^H151Q^ (Fig. 2A, right panel; quantified in Fig. 2B, C). CaN downregulating cells (Supplementary Fig. 4) also exhibited a significant decrease in Parkin recruitment upon CCCP treatment (Fig. 2D, quantified in Fig. 2E, F).

### Parkin translocation is induced by Calcineurin in the absence of PINK1 but depends on Miro1 expression

**Fig. 3:**
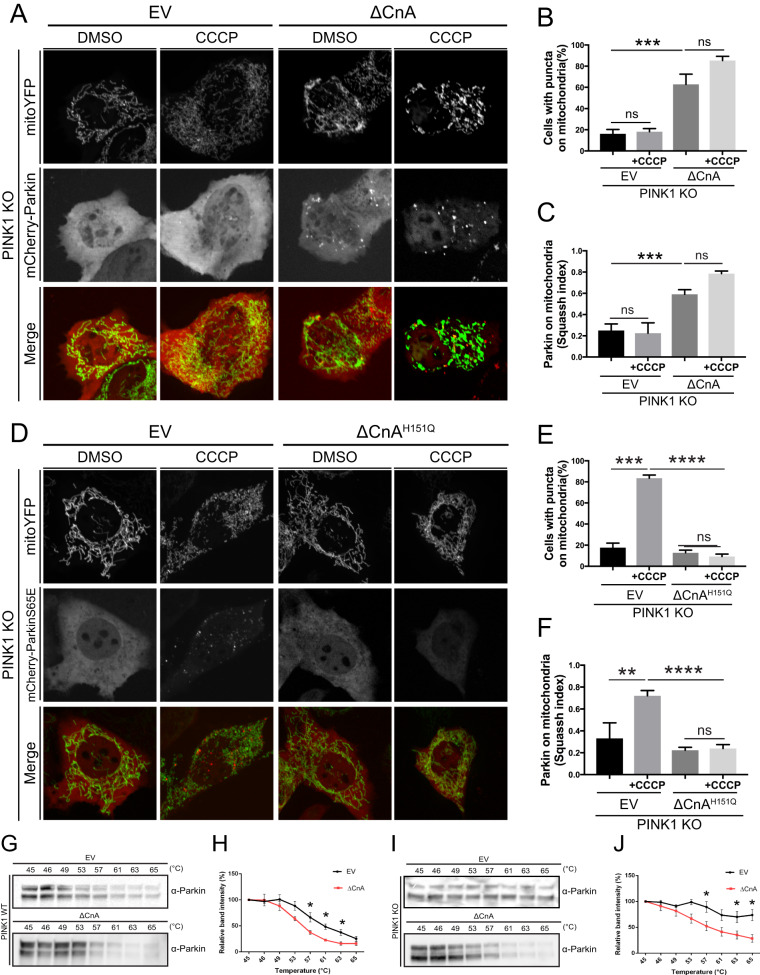
Parkin translocation is induced by Calcineurin in the absence of PINK1. **A** Representative confocal images of PINK1 KO MEF cells transfected with mCherry-Parkin, mito-YFP and with empty vector (EV) or constitutively active CaN (ΔCnA). **B** Quantification of **A** Graph bar shows mean ± SEM of percentage of cells with mCherry-Parkin on mitochondria for at least ≥ 300 cells per biological replicate. Two-way ANOVA followed by Tukey’s multiple comparison test (*n* = 3-4; p < 0.001). **C** Quantification of **A** using Squassh. The graph bars show mean ± SEM of Squassh colocalization coefficient for at least ≥ 50 images per biological replicate. 0 = no colocalization, 1 = perfect colocalization. At least 3 independent experiments were performed. Two-way ANOVA followed by Tukey’s multiple comparison test (*n* = 3-4; *p* < 0.001). **D** Representative confocal images of PINK1 KO MEF cells transfected with mCherry-ParkinS65E, UbS65E, mito-YFP and with empty vector (EV) or dominant negative CaN (ΔCnA^H151Q^). **E** Quantification of **D** Graph bar shows mean ± SEM of percentage of cells with mCherry-Parkin on mitochondria for at least ≥ 300 cells per biological replicate. Two-way ANOVA followed by Tukey’s multiple comparison test (*n* = 4; *p* < 0.0001). **F** Quantification of **D** using Squassh. The graph bars show mean ± SEM of Squassh colocalization coefficient for at least ≥ 50 images per biological replicate. 0 = no colocalization, 1 = perfect colocalization. Two-way ANOVA followed by Tukey’s multiple comparison test (*n* = 4; *p* < 0.0001). **G** Parkin thermal stability assay. WT MEFs expressing constitutive active CaN (ΔCnA) or empty vector (EV) were suspended in PBS and snap-freezed in liquid nitrogen before being aliquoted into a PCR strip and incubated at the indicated temperature for 3 min. The lysates were centrifugated at high speed and the soluble fraction was loaded into SDS-PAGE gel. Representative Western blotting analysis for Parkin stability is shown. **H** Densitometric analysis of **G**. Student’s t-test (*n* = 4; *p* < 0.05). **I** Parkin thermal stability assay. PINK1 KO MEFs expressing constitutive active CaN (ΔCnA) or empty vector (EV) were suspended in PBS and snap-freezed in liquid nitrogen before being aliquoted into a PCR strip and incubated at the indicated temperature for 3 min. The lysates were centrifugated at high speed and the soluble fraction was loaded into SDS-PAGE gel. Representative Western blotting analysis for Parkin stability is shown. **J** Densitometric analysis of **I**. Student’s t-test (*n* = 6; *p* < 0.05).

Parkin translocation and activity are strictly controlled by mechanisms of autoinhibition that can be released by PINK1 [13, 15, 16, 68–70]. The current model for the mechanism of Parkin activation and recruitment by PINK1 depicts that under basal conditions Parkin activity is repressed and the protein maintains a close structure with the ubiquitin-like domain (UBL) and the repressor element of Parkin (REP) fragment occluding the RING1 domain and the RING0 domain impeding on the catalytic site-containing RING2 domain [14–16]. Structural studies have provided evidence that phosphorylation of Ubiquitin and Parkin at Serine 65 by PINK1 causes displacement of the inhibitory UBL and stretches the REP [42]. This affects Parkin autoinhibitory structure and contributes to promote Parkin conformational change that leads to Parkin mitochondrial recruitment and activation [42, 71, 72]. In line with this model and as already reported [32, 38], we found that Parkin does not translocate to CCCP-treated mitochondria in the absence of PINK1 (Fig. 3A, left panel). Intriguingly, expression of constitutive active CaN (CnB plus ΔCnA) promoted Parkin translocation in PINK1 KO cells, even in the absence of CCCP (Fig. 3A, right panel; quantified in Fig. 3B, C), a condition that was hold true also in PINK1 wild-type cells (Supplementary Fig. 5). These microscopy evidences were further supported by cell fractionation experiments (Supplementary Fig. 6). Moreover, in PINK1 KO MEFs transfected with phospho-mimetic Ub (Ub S65E), a large proportion of phospho-mimetic mCherry-Parkin (Parkin S65E) translocated to CCCP-treated mitochondria (Fig. 3D, left panel), a condition that was abolished upon expression of CaN dominant negative ΔCnA^H151Q^ (Fig. 3D, right panel; quantified in Fig. 3E, F). Based on these results, it is expected that the expression of CaN can affect Parkin conformational change in the absence of PINK1 to promote Parkin translocation. Because conformational changes are paralleled by changes in protein solubility, which can be assessed by thermal shift [73, 74], we performed a thermal stability assay for Parkin to test this hypothesis. Expression of constitutive active CaN leads to a decrease in Parkin thermal stability in wild type (Fig. 3G, H) and PINK1 KO cells (Fig. 3I, J), supporting the hypothesis that Parkin undergoes a conformational change in this condition regardless PINK1 expression.

**Fig. 4:**
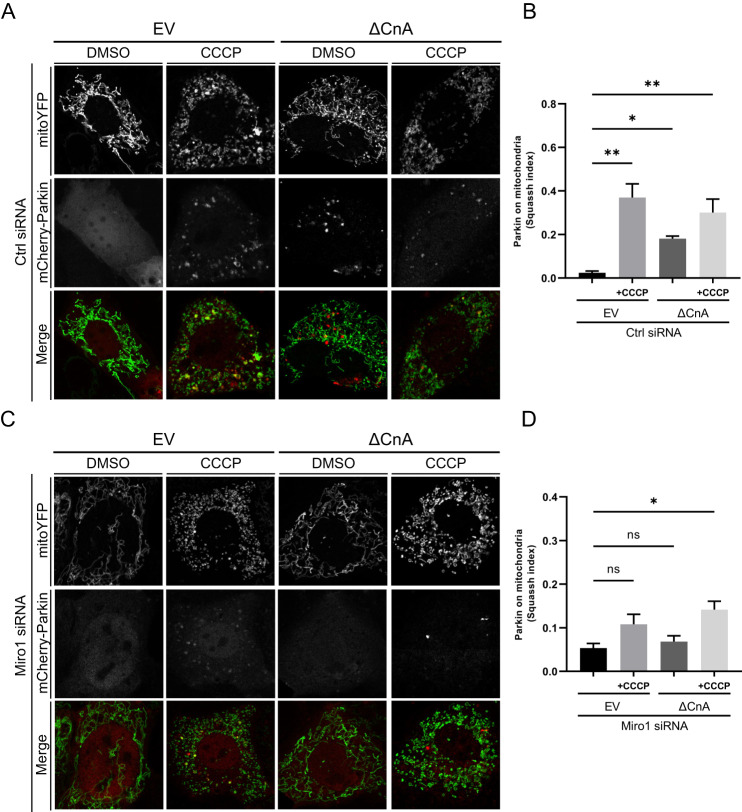
Parkin translocation induced by Calcineurin is Miro1-dependent. **A** Representative confocal images of Ctrl siRNA MEF cells transfected with mCherry-Parkin, mito-YFP and with empty vector (EV) or constitutively active CaN (ΔCnA) for 2 days before being treated with DMSO or 10 μM CCCP for 3 h. **B** Quantification of **A** using Squassh. The graph bars show mean ± SEM of Squassh colocalization coefficient for at least ≥ 10 images per biological replicate. 0 = no colocalization, 1 = perfect colocalization. Two-way ANOVA followed by Holm-Sidak multiple comparison test (*n* = 3; *p* < 0.01). **C** Representative confocal images of Miro1 siRNA MEF cells transfected with mCherry-Parkin, mito-YFP and with empty vector (EV) or constitutively active CaN (ΔCnA) for 2 days before being treated with DMSO or 10 μM CCCP for 3 h. **D** Quantification of **C** using Squassh. The graph bars show mean ± SEM of Squassh colocalization coefficient for at least ≥ 10 images per biological replicate. 0=no colocalization, 1 = perfect colocalization. Two-way ANOVA followed by Holm-Sidak multiple comparison test (*n* = 3; *p* < 0.01).

The fact that Parkin translocation induced by CaN is PINK1 independent was unexpected. Of note, the effect of CaN on Parkin translocation is not secondary to membrane potential alteration because we did not record any significant difference in mitochondrial membrane potential upon expression of constitutive active CaN (Supplementary Fig. 7). Thus, PINK1 is presumably not stabilized on the outer mitochondrial membrane to promote Parkin recruitment because it is imported by functional mitochondrial import machinery and degraded [32]. Based on these evidences, we explored the potential involvement of Miro1 in Parkin translocation. Miro1 is a mitochondrial outer membrane Rho GTPase that binds Ca^2+^, which was previously showed to attract cytosolic Parkin to mitochondria in a PINK1-independent fashion, and without affecting the mitochondrial membrane potential [75]. We tested Parkin translocation in control (Fig. 4A) and Miro1 downregulating cells (Fig. 4B and Supplementary 8A, B). As previously reported [75], we observed impairment in Parkin mitochondrial recruitment in CCCP-treated Miro1 KD cells (Fig. 4C; quantified in Fig. 4D). Importantly, expression of constitutive active CaN (ΔCnA), which promotes Parkin translocation in control cells (Fig. 4A; quantified in Fig. 4B), failed to induce mitochondrial translocation of Parkin when Miro1 is downregulated (Fig. 4C; quantified in Fig. 4D). In conclusion, expression of constitutively active CaN promotes Parkin translocation in PINK1 KO cells, but requires Ca^2+^ -binding protein Miro1 for Parkin mitochondrial recruitment.

### Calcineurin interacts with Parkin

**Fig. 5:**
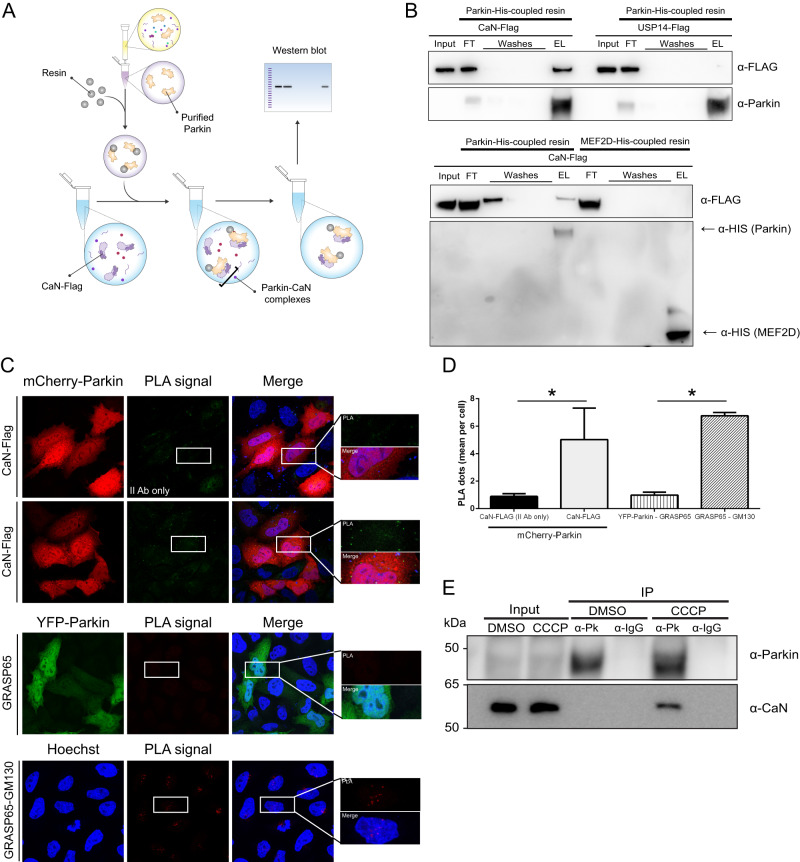
Calcineurin interacts with Parkin. **A** In vitro interaction assay: schematic representation. Affinity-purified recombinant His-tagged Parkin is produced from bacteria, and coupled to a His-affinity resin to generate Parkin-His-resin. The resin is incubated with protein lysate extracted from cells expressing Flag-tagged CaN. The resin is washed to remove nonspecifically adhering proteins, and Imidazole is used to elute the complexes from the resin. The obtained eluate is separated by SDS-PAGE and analyzed by immunoblotting. **B** In vitro interaction assay. His-tagged Parkin or His-tagged MEF2D coupled to a His-affinity resin are incubated with cell lysate obtained from MEFs transfected with CaN-Flag or USP14-Flag, as indicated. The eluate is separated by SDS-PAGE and analyzed by immunoblotting using anti-Flag, anti-Parkin and anti-His antibodies (FT = flow through; EL=eluate). **C** Representative images of Proximity Ligation Assay (PLA) performed on HeLa cells transfected with Parkin-mCherry and CaN-FLAG (PPP3CB-FLAG). After fixation, cells were processed for investigating Parkin proximity interactions by incubating with anti-Parkin antibody and anti-PPP3CB antibody, and corresponding secondary antibodies, or secondary antibodies alone as negative control. CaN and Parkin interactions are represented by green dots. As negative biological control for PLA we used anti-Parkin antibody and anti-GRASP65 antibody in YPF-Parkin expressing HeLa cells. As positive control for PLA, we used anti-GRASP65 antibody and anti-GM130 antibody. GRASP65 is a peripheral membrane protein that resides in the *cis-*Golgi apparatus, and interacts with GM130 (also expressed in the *cis*-Golgi network) but not with Parkin. GRASP65 and GM130 interactions are represented by red dots. White squares contain higher-magnification images. **D** Quantification of C. Graph bar shows mean ± SEM of PLA dots per cell for at least ≥ 350 cells per biological replicate. Student’s t-test (*n* = 3-4; *p* < 0.05). **E** HEK 293 T cells were treated with DMSO or CCCP-2 h and subjected to immunoprecipitation (IP) of Parkin using mouse anti-Parkin antibody or anti-mouse IgG as negative control. Western Blot analysis was performed with rabbit anti-CaN antibody or mouse anti-Parkin antibody on the pulled-down samples. Inputs represent 5% of the protein lysates and IP eluate 100% of the protein lysates. Mouse IgG TrueBlot® ULTRA enabled detection of Parkin band, without hindrance by interfering immunoprecipitating IgG heavy chains.

Our results prompted us to evaluate the possibility of an interaction between CaN and Parkin. To evaluate this possibility, we first performed an in vitro interaction assay by generating affinity-purified recombinant His-tagged Parkin from bacteria, which was coupled to a His-affinity resin and incubated with protein lysate extracted from cells expressing Flag-tagged CaN. The resin was washed to remove nonspecifically adhering proteins, and Laemmli buffer was used to elute the complexes from the resin (Fig. 5A). Importantly, in the eluted complexes we were able to identify CaN (Fig. 5B), indicating that CaN binds to Parkin in vitro. The interaction was specific for CaN because an unrelated Flag-tagged protein that is not supposed to interact with Parkin (USP14-Flag) was not retrieved in the eluted complexes when protein lysate from cells expressing USP14-Flag was incubated with the resin. Likewise, no interaction was retrieved when His-tagged MEF2D, which does not interact with CaN, was used as a bait to retrieve CaN (Fig. 5B).

We next performed a proximity ligation assay (PLA) to investigate Parkin and CaN interaction in situ. We were able to visualize discrete spots (PLA signal) in HeLa cells co-expressing mCherry-Parkin and CaN-Flag, representative of Parkin-CaN close proximity. In this assay, the interaction between GRASP65 and Parkin was used as negative biological control, whereas the interaction between GRASP65 and GM130 was used as positive control for PLA [76, 77]. PLA detection dots indicate positive interaction (Fig. 5C; quantified in Fig. 5D). The specificities of the antibodies used in the PLA were tested by immunofluorescence (Supplementary Fig. 9).

Finally, to evaluate the possibility of an interaction at the endogenous level, we performed an immunoprecipitation (IP) assay in HEK 293 T cells, which express relatively high levels of endogenous Parkin. HEK cells were treated with DMSO or CCCP for 2 h, and endogenous Parkin from cell lysate was captured by specific Parkin antibody. The antibody-protein complexes were pulled out of the sample using Protein A-coupled agarose beads. Endogenous CaN co-immunoprecipitated with Parkin in these complexes, indicating that endogenous CaN and Parkin interact. Notably, the interaction between endogenous Parkin and CaN was detectable only upon CCCP treatment to activate CaN, and not under basal conditions (Fig. 5E). To further strengthen this result, we also performed the reverse IP, i.e. we pulled down endogenous CaN from lysate of cells treated with CCCP as before, and we were able to retrieve endogenous Parkin from the pulled down sample (Supplementary Fig. 10).

Thus, CaN interacts with Parkin.

### Parkin translocation is induced by Calcineurin independently of Drp1 mitochondrial recruitment and activity

**Fig. 6:**
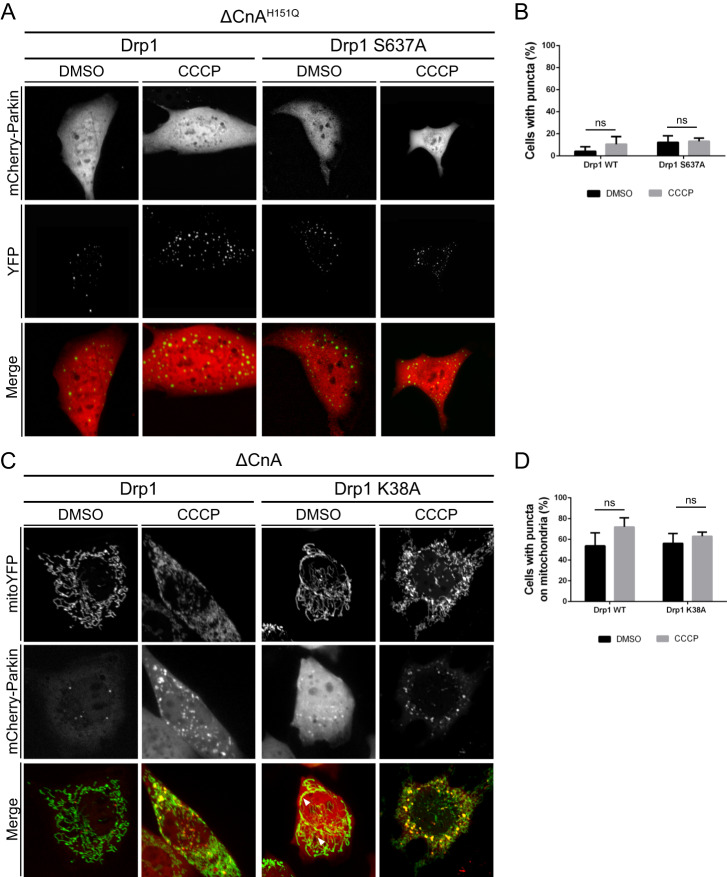
Parkin translocation is induced by Calcineurin independently of Drp1 mitochondrial recruitment and activity. **A** Representative confocal images of wild type MEF cells transfected with mCherry-Parkin and YFP-Drp1 or YFP-Drp1 S637A plus dominant negative CaN (ΔCnA^H151Q^) for 2 days before being treated with DMSO or 10 μM CCCP for 3 h. **B** Quantification of A. Graph bar shows mean ± SEM of percentage of cells with mCherry-Parkin puncta for ≥ 300 cells per biological replicate. Two-way ANOVA followed by Tukey’s multiple comparison test (*n* = 3). **C** Representative confocal images of wild type MEF cells transfected with mCherry-Parkin, mito-YFP and constitutive active CaN (ΔCnA) plus Drp1 or Drp1 K38A for 2 days before being treated with DMSO or 10 μM CCCP for 3 h. **D** Quantification of **C**. Graph bar shows mean ± SEM of percentage of cells with mCherry-Parkin on mitochondria for ≥ 300 cells per biological replicate. Two-way ANOVA followed by Tukey’s multiple comparison test (*n* = 3).

Previous reports demonstrated that CaN regulates the phosphorylation status of mitochondrial pro-fission protein Drp1, and that dephosphorylation by CaN on Serine 637 is required for Drp1 translocation and in the process of mitochondrial fission [51]. Because CaN activation promotes mitochondrial recruitment of Drp1 and Parkin, we addressed whether these two events were correlated. Interestingly, expression of constitutive dephosphorylated Drp1 (Drp1 S637A) that was shown to be mostly mitochondrial and to promote mitochondrial fragmentation [51], did not affect Parkin translocation in the presence of CaN dominant negative (Fig. 6A, B). Moreover, promotion of Parkin recruitment by expression of constitutive active CaN was not inhibited in the presence of Drp1 dominant negative (Drp1 K38A) (Fig. 6C, D), indicating that Parkin translocation is independent of Drp1 recruitment to the mitochondria to drive mitochondrial fragmentation.

### Calcineurin is required for CCCP-induced mitophagy

Our data demonstrate that CaN interacts with Parkin, and that CaN activity is required for Parkin recruitment to mitochondria. What about mitophagy? We used four different approaches to evaluate mitophagy in vitro in MEFs, namely (i) quantification of mitochondrial mass, estimated by western blotting analysis of inner mitochondrial membrane protein ATP synthase (ATP5A), (ii) confocal analysis of LC3-decorated mitochondria, (iii) FACS and confocal analysis of mitochondria-targeted fluorescent probe mt-Keima, and (iv) electron microscopy (EM).

**Fig. 7:**
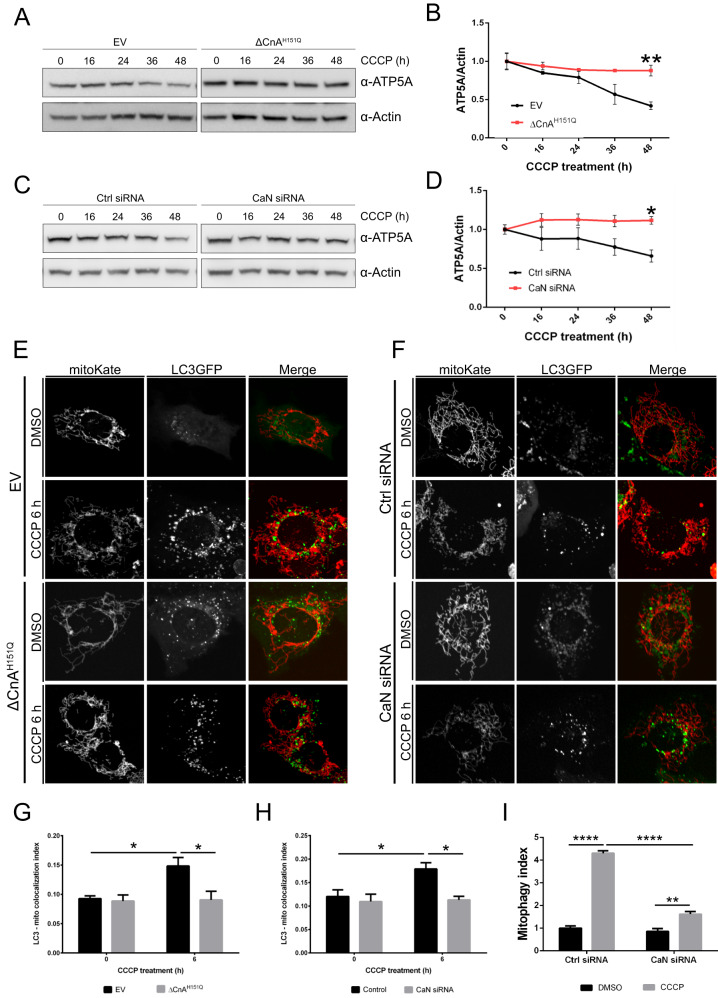
Calcineurin is required for CCCP-induced mitophagy. **A** Western blot analysis of protein lysates extracted from MEFs expressing empty vector (EV) or dominant negative CaN (ΔCnA^H151Q^). Cells were treated with 10 μM CCCP for the indicated time. **B** Quantification of **A**. Line chart shows mean ± SEM of ATP5A protein level normalized to Actin. Student’s t-test (*n* = 3; *p* < 0.01). **C** Western blot analysis of protein lysates extracted from MEFs downregulating Calcineurin and control. Cells were treated with 10 μM CCCP for the indicated time. **D** Quantification of **C**. Line chart shows mean ± SEM of ATP5A protein level normalized to Actin. Student’s t-test (*n* = 5; p < 0.05). **E** Representative confocal images of cells transfected with LC3GFP and MitoKate plus dominant negative CaN (ΔCnA^H151Q^) or empty vector (EV). **F** Representative confocal images of cells transfected with LC3GFP and MitoKate in CaN downregulating condition and matching control. **G** Quantification of **E** using Squassh. Two-way ANOVA followed by Tukey’s multiple comparison test (*n* = 3; *p* < 0.05). **H** Quantification of **F** using Squassh. Two-way ANOVA followed by Tukey’s multiple comparison test (*n* = 3; *p* < 0.05). **I** mt-Keima analysis in CaN downregulation condition upon CCCP treatment. mt-Keima demonstrates a greater than 4-fold change in ratiometric fluorescence in CCCP-treated cells, which was blunted upon CaN downregulation. Two-way ANOVA followed by Tukey’s multiple comparison test (*n* = 3; *p* < 0.001).

Because MEFs have negligible levels of Parkin [43, 78–80], which might affect our ability to investigate Parkin-dependent events, we utilized a well established experimental system [34, 45, 78, 81, 82] in which we introduced exogenous Parkin into MEFs by generating a Parkin-flag stable cell line by retroviral infection [83]. MEFs stably expressing Parkin-flag were transfected with dominant negative CaN (ΔCnA^H151Q^) or corresponding empty vectors, and mitochondrial mass was assessed following treatment with CCCP. In this condition, mitochondrial protein ATP synthase subunit alpha, ATP5A was lost after 36 h CCCP treatment, while it was retained in cells expressing dominant negative CaN, ΔCnA^H151Q^ (Fig. 7A, B). Cells downregulating CaN also exhibited impaired CCCP-induced mitochondrial degradation (Fig. 7C, D). These quantitative immunoblotting data were confirmed by confocal analysis of colocalization of mito-Kate labelled mitochondria with GFP-LC3 labelled autophagosomes. Cells expressing dominant negative CaN (Fig. 7E) or downregulating CaN (Fig. 7F) presented impaired LC3-mitochondria colocalization, following CCCP (quantified in Fig. 7G, H). To further evaluate mitophagy in our model system, we infected Parkin expressing MEFs with mt-Keima, a pH-dependent fluorescence probe targeted to the mitochondrial matrix, which has different excitation spectra at neutral and acidic pH [84]. Keima has a single emission peak at 620 nm with a bimodal excitation spectrum. These properties of mt-Keima allow rapid FACS determination of “acidic” mitochondria undergoing autolysosome degradation. The mt-Keima assay showed that CCCP-induced mitophagy was greatly reduced in cells downregulating CaN, in line with the results obtained with the biochemical approaches (Fig. 7I and Supplementary Fig. 11).

### Constitutive active Calcineurin induces an increase in basal mitophagy

**Fig. 8:**
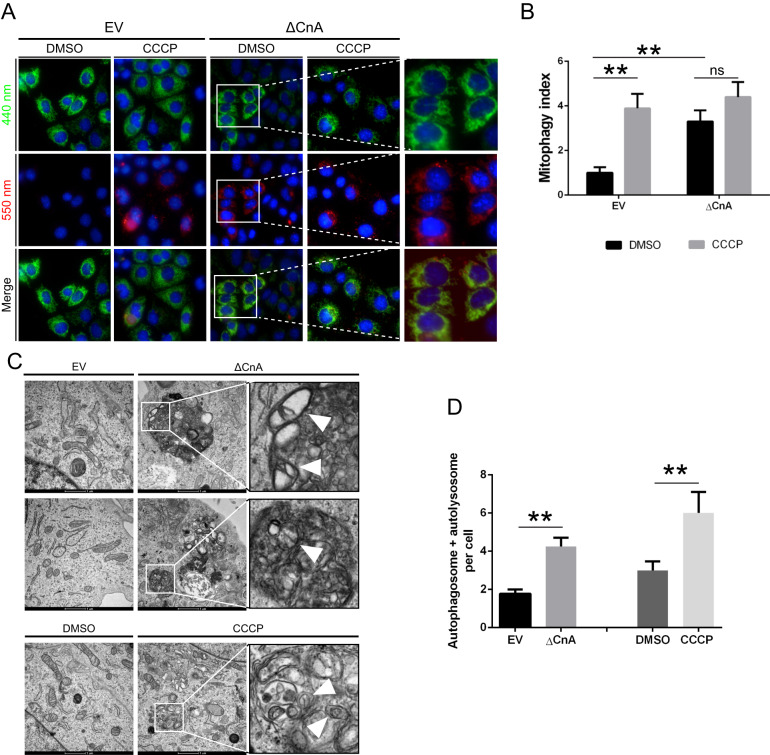
Calcineurin is required for CCCP-induced mitophagy. **A** Representative image of mt-Keima expressing cells acquired on confocal microscope Operetta High-Content Imaging system. MEFs were transfected with constitutive active CaN (ΔCnA) or empty vector (EV). **B** mt-Keima analysis in MEFs expressing constitutive active CaN (ΔCnA) and relative control (EV). Two-way ANOVA followed by Tukey’s multiple comparison test (*n* = 6; *p* < 0.01). **C** Representative electron microscope images showing the presence of autophagosomes with mitochondrial-like structures inside in MEFs expressing constitutive active CaN (ΔCnA) and control (EV). 24 h CCCP-treated cells were used as positive control. Mitochondrial-like structures were identified as described in Chakraborty et al. [130]. **D** Bar graph represents mean ± SEM of the number of autophagosomes and autolysosomes per cell. At least 60 cells per biological replicate were analyzed from each group. Student’s t-test (*n* = 3-4; *p* < 0.01).

We previously demonstrated that the expression of constitutive active CaN promotes Parkin translocation even in absence of CCCP treatment. To address whether this was also the case for mitophagy, we performed mt-Keima assay in Parkin expressing MEFs transiently transfected with constitutively active CaN. Representative image of mt-Keima acquired at confocal microscope clearly showed increased number of red fluorescence in control cells expressing constitutively active CaN, ΔCnA (Fig. 8A). In these cells basal mitophagy (i.e. untreated cells) was increased (Fig. 8B). Qualitative electron microscopy demonstrated an increase in autophagosomes and autolysosomes with mitochondrial like-structures inside in this condition (Fig. 8C, D).

### Calcineurin promotes Parkin translocation and activation in the absence of PINK1 but requires PINK1 to promote mitophagy in MEFs

**Fig. 9:**
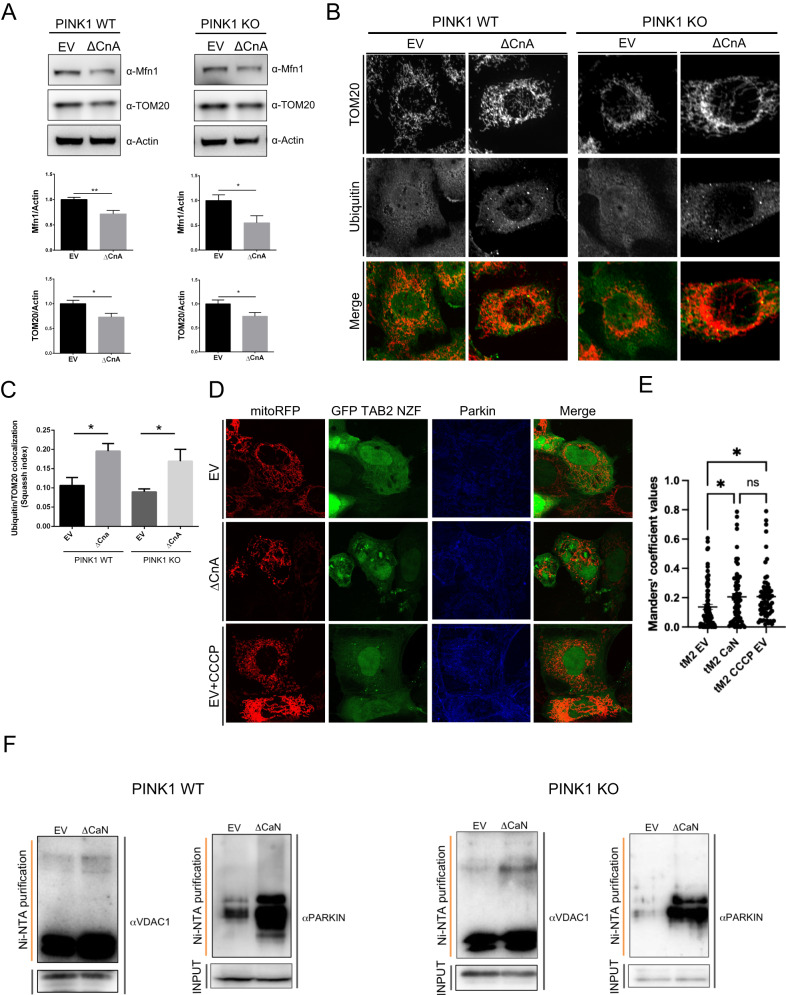
Calcineurin requires PINK1 to induce mitophagy in MEFs. **A** Representative Western blot analysis of protein lysates extracted from PINK1 WT and PINK1 KO MEFs. Cells of the indicated genotype were transfected with constitutive active CaN (ΔCnA) or empty vector (EV). Graph bars shows mean ± SEM of indicated mitochondrial protein level normalized to Actin. Student’s t-test (*n* = 8-11; *p < 0.05; **p < 0.01). **B** Representative confocal images of cells of the indicated genotype transfected with constitutive active CaN (ΔCnA) or empty vector (EV). Cells were pretreated with proteasome inhibitor MG132 before being fixed, permeabilized and incubated with the indicated primary antibodies and corresponding fluorophore-conjugated secondary antibodies. **C** Quantification of **B** using Squassh. Two-way ANOVA followed by Tukey’s multiple comparison test (*n* = 4; *p* < 0.05). **D** Representative confocal images of MEFs transfected with constitutive active CaN (ΔCnA) or empty vector (EV), and with the GFP-TAB2 NZF sensor for K63-linked ubiquitin chain, and mitoRFP to visualize the mitochondrial network. In one group 10μM CCCP was used as a positive control to trigger Parkin translocation and Parkin-dependent ubiquitination of mitochondria. **E** Quantification of **D**. Graph bars shows the co-localisation of k63 linked ubiquitin probe (GFP-TAB2 NZF) with mitochondria. Co-localisation index was analysed with Coloc2 plugin (Fiji). tM2 = fraction of GFP TAB2 NZF co-localising with mitochondria (0 = null, 1 = 100%). **F** Representative Western blot analysis of protein lysates extracted from PINK1 WT and PINK1 KO MEFs, and incubated with the indicated antibodies, after being immunoprecipiated with HisPur Ni-NTA Magnetic Beads (Thermoscientific). Cells of the indicated genotype were transfected with His-Ubiquitin and constitutive active CaN (ΔCnA) or empty vector (EV). Cells were pretreated with proteasome inhibitor MG132 for 4 h before being collected.

While investigating Parkin translocation in PINK1 KO background, we came across the unexpected finding that expression of constitutive active CaN triggered Parkin translocation in the absence of PINK1 (Fig. 3). Parkin translocation induced by CaN is independent of Drp1 activity and Drp1 mitochondrial recruitment to promote mitochondrial fission (Fig. 6). Based on these observations, we now want to address the potential “mitophagic” effect of CaN activation in PINK1 KO background. Unexpectedly, expression of constitutive active CaN in PINK1 KO cells failed to promote the degradation of mitochondrial protein ATP5A (Supplementary Fig. 12A, B). These quantitative immunoblotting results were confirmed by mt-Keima assay (Supplementary Fig. 12C). Analysis of protein levels of bona-fide Parkin substrates [47] revealed that the levels of TOM20 and Mfn1 were decreased when constitutive active CaN was expressed in WT and PINK1 KO background (Fig. 9A). In this condition, we also tracked an increase in the coefficient of colocalization between Ubiquitin and mitochondria, stained with TOM20 (Fig. 9B, C), and in the mitochondrial recruitment of K-63-linked ubiquitin chains [85], known to be Parkin-dependent [45](Fig. 9D, E).More importantly, ubiquitination levels of critical Parkin targets, VDAC [86] and Parkin [47], were increased upon expression of constitutive active CaN (ΔCnA), a condition that was hold true also in PINK1 KO cells (Fig. 9F).

Thus, in PINK1 KO MEFs expressing constitutive active CaN, mitochondria cannot be efficiently eliminated by mitochondrial autophagy. Nevertheless, in this condition, Parkin appears to be on ubiquitinated mitochondria, protein levels of TOM20 and Mfn1 are decreased, while ubiquitination levels of its crucial targets VDAC and Parkin are increased.

### Constitutive active Calcineurin corrects locomotor ability and mitochondrial function of PINK1 KO flies

**Fig. 10:**
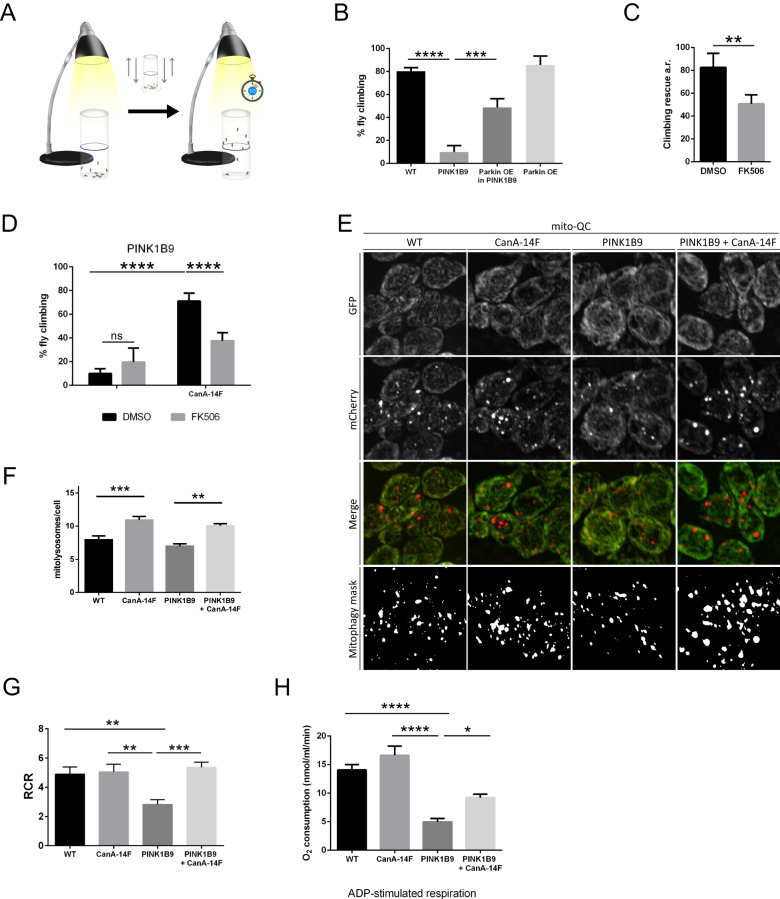
Constitutive active Calcineurin corrects locomotor ability and mitochondrial function of PINK1 KO flies. **A** Schematic representation of the climbing assay. 10 flies were put into a tube in a dark room. A light was put on the top of the tube. After tapping the flies at the bottom of the tube, the number of flies that successfully climbed above the 6-cm mark after 10 s was recorded. **B** Graph bar shows mean ± SEM of the climbing performance of flies of the indicated genotype. One-way ANOVA followed by Sidak’s multiple comparison test (*n* = 5; *p* ≤ 0.001). **C** Graph bar shows mean ± SEM of the climbing rescue (arbitrary unit) of PINK1 KO flies (PINK1B9) overexpressing Parkin and treated as indicated for 48 h with DMSO or FK506. Student’s t-test (*n* = 5; *p* ≤ 0.01). **D** Graph bar shows mean ± SEM of the climbing performance of PINK1 KO flies (PINK1B9) upon expression of constitutive active calcineurin (CanA-14F), and treated as indicated for 48 h with DMSO or FK506. Two-way ANOVA followed by Sidak’s multiple comparison test (*n* = 5; *p* ≤ 0.001). **E** Representative confocal microscopy images of neurons in the ventral nerve chord of third instar larvae. Fluorescence corresponding to neutral pH (GFP) and acidic pH (mCherry) are shown. Mitolysosomes are counted as GFP-negative/mCherry-positive (red-only) puncta. The mitophagy mask generated by the mito-QC Counter allows visualization of the quantified mitophagy areas [129]. **F** Quantification of total number of mitolysosomes per cell using the mito-QC Counter [129] (Fiji). At least 40 cells were analyzed per animal. One-way ANOVA followed by Sidak’s test for multiple comparisons (*n* = 6; *p* ≤ 0.01). **G** Quantitative analysis of respiratory fitness of mitochondria from adult flies of the indicated genotype. Graph shows respiratory control ratio (RCR) calculated as described in Materials and Methods. One-way ANOVA followed by Sidak’s test for multiple comparison test (*n* = 10–11, *p* ≤ 0.01). **H** Quantitative analysis of respiratory fitness of mitochondria from adult flies of the indicated genotype. Graph bar shows ADP-stimulated respiration calculated as described in Materials and Methods. One-way ANOVA followed by Sidak’s test for multiple comparison test (*n* = 10-11, *p* ≤ 0.01).

To evaluate the physiological relevance of our finding in vivo, we turned to a well-established *drosophila* model of mitochondrial dysfunction that was successfully used in many studies before: the PINK1 KO flies [87–94]. At the systemic level, PINK1 KO flies show characteristic locomotor defects in flight and climbing ability, degeneration of muscle fibers of the thorax and reduced lifespan. The phenotype of PINK1 KO flies can be rescued by overexpression of Parkin, whereas Parkin KO phenotype, which phenocopies PINK1 KO, cannot be rescued by PINK1 overexpression, and combined mutation of PINK1 and Parkin genes does not exacerbate the phenotype [88–90]. These evidences have been interpreted as PINK1 and Parkin interacting functionally in a linear pathway with PINK1 operating upstream of Parkin [88–90]. With that in mind and to assess the role of CaN in this pathway, we evaluated the effect of CaN inhibition and activation in vivo in PINK1 KO flies. To do so, we used a locomotor assay in which 10 flies for each strain were collected in a vertical plastic tube positioned with a line drawn at 6 cm from the bottom of the tube and under a light source. After tapping the flies to the bottom of the tube, the flies that successfully climbed above the mark after 10 s were counted (Fig. 10A). As already reported [89, 90], PINK1 KO flies (PINK1B9) performed poorly in the climbing assay compared to wild type (Fig. 10B), and Parkin overexpression (Parkin OE) rescued PINK1 KO climbing defects (Fig. 10B). Feeding PINK1 KO flies with CaN specific inhibitor FK506 diminished the rescuing effect of Parkin overexpression on climbing performance (Fig. 10C), while FK506 administration alone did not show any adverse effects on the climbing ability of PINK1 KO flies (Fig. 10D). Importantly, expression of CaN constitutive active (CanA-14F) [71] in PINK1 KO background rescued PINK1 KO climbing defects (Fig. 10D), an effect that was partially suppressed by feeding flies with CaN inhibition FK506 (Fig. 10D). Moreover, expression of constitutive active CaN enhanced basal mitophagy in WT and PINK1 KO flies (Fig. 10E, F), and rescued PINK1 KO mitochondrial respiratory defects (Fig. 10G, H). Thus, constitutive active CaN corrects locomotor behaviour and mitochondrial function of PINK1 KO flies.

## Discussion

Mitophagy, a selective kind of autophagy during which defective mitochondria are recognized and degraded, depends on Serine/threonine kinase PINK1 and E3 ubiquitin ligase Parkin [95], two genes which mutations have been linked to the onset of an autosomal recessive juvenile parkinsonism [9, 96]. Previous studies independently showed that during stress-induced mitophagy, the E3 ubiquitin ligase Parkin translocates in a PINK1-dependent manner to depolarized mitochondria [33, 38, 45]. In this process, kinase PINK1 phosphorylates Ubiquitin [42], Parkin [97] and its targets [48, 64], and promotes mitochondrial Parkin translocation [38] and Parkin activity [42, 98]. On depolarized mitochondria, Parkin ubiquitinates mitochondrial pro-fusion proteins Mitofusin (Mfn) [38, 46, 47, 99, 100] leading to its chaperone p97/VCP-mediated retrotranslocation for proteosomal degradation [46]. In addition, Parkin ubiquitinates the mitochondrial protein translocase TOM20, mitochondrial VDAC/Porin and Fis1 [47], and it also promotes the degradation of Miro [64], a protein that couples mitochondria to microtubules. Selected mitochondria are therefore deprived of their pro-fusion protein Mfn, they are isolated from the mitochondrial network, and driven to autophagic degradation via autophagy adaptors like p62 (SQSTM1) [101], HDAC6 [102], Optineurin and NDP52 [103]. As shown in many studies, CCCP treatment is one of the most consolidated stimuli to promote Parkin translocation and stress-induced mitophagy. CCCP triggers mitochondrial depolarization by transporting protons inside the mitochondrial matrix, and induces a transient increase of cytosolic Ca^2+^ influx [56] that leads to CaN activation [51]; CaN activation promotes Drp1 translocation to mitochondria to induce mitochondrial fission [51], and the transcription of autophagy and lysosomal genes via TFEB [57]. In perfect agreement with this coordinated set of events, we found that activated CaN interacts with the mitophagic protein Parkin, and promotes Parkin mitochondrial recruitment. Importantly, the effect of CaN activation on mitochondrial recruitment of Parkin is PINK1-independent, and does not affect mitochondrial membrane potential. Also, Parkin translocation induced by CaN is Miro1-dependent. A recent work highlighted the importance of Miro1-dependent recruitment of Parkin on mitochondria under basal conditions, which occurs independently of PINK1 expression and does not require mitochondrial membrane depolarization. In this respect, Miro1 operates as mitochondrial docking site for Parkin under basal conditions that facilitates/accelerates its recruitment when needed [75]. Similar mechanisms of docking substrates have been suggested for other Parkin substrates located on mitochondria to favour efficient and rapid Parkin recruitment [104, 105], and specifically in the absence of PINK1 activation i.e. under basal conditions.

Indeed, Parkin recruitment and Parkin-dependent mitophagy has been described in the absence of mitochondrial membrane potential depolarization, following proteotoxic stress [54, 106] or upon specific induction of mitochondrial Ca^2+^ oscillation [107]. In this scenario, PINK1 is presumably not stabilized on the outer mitochondrial membrane to promote Parkin recruitment because it is imported by a functional mitochondrial import machinery, and rapidly degraded [32]. Also, PINK1-independent mechanisms of Parkin recruitment have been previously described [108]. These studies suggest that Parkin recruitment and Parkin-dependent mitophagy can be activated upon stimuli, which do not necessarily culminate in mitochondrial damage or mitochondrial ROS production, and more importantly that alternative PINK1-independent mechanisms of Parkin recruitment can be predicted. Our study further supports the existence of these alternative mechanisms of Parkin recruitment that are PINK1-independent. In this work we show that not only Parkin can be recruited to mitochondria under basal conditions (i.e. not to depolarized mitochondria) in a PINK1-independent fashion, but also that Parkin ubiquitinates its targets in the absence of PINK1, indicating that Parkin is presumably active in this condition. It is possible that additional kinases other than PINK1 can phosphorylate Parkin and Ubiquitin for Parkin activation. Parkin is a heavily phosphorylated protein, and several kinases have been reported to phosphorylate Parkin (for example AMPKA1 [109], CDK5 [110], PLK1 [111], ULK1 [112] and GSK3β [113]). It is possible that one of these kinases or another kinase yet to be identified are responsible for Ubiquitin phosphorylation and Parkin activation, or that phospho-ubiquitin independent mechanisms of Parkin activation, such as Parkin neddylation [113, 114], are triggered by CaN activation. Undoubtedly, the mechanism underlying activation of Parkin is not completely unravelled, and remains fertile ground for further debate.

Interestingly, although Parkin is efficiently recruited to mitochondria in PINK1 KO cells expressing constitutive active CaN, this is not sufficient to promote mitophagy in vitro in MEFs. As shown in many in vitro studies, the autophagic engulfment of mitochondria need to be paralleled by a tight control of mitochondrial size, and mitochondria need to fragment prior to mitophagy [59]. Because PINK1 inhibits PKA-mediated phospho-inhibition of Drp1 [50] and it directly phosphorylates Drp1 on S616 to regulate mitochondrial dynamic [52], PINK1 expression is required to release Drp1 inhibition and promote its mitochondrial recruitment to initiate mitochondrial fragmentation. Following mitochondria damage, PINK1 becomes active, disrupts the AKAP1-PKA and phosphorylates Drp1 to promote Drp1-dependent fission [50], which is required to execute mitophagy. Thus, it is possible that in MEFs expression of CaN does not promote mitophagy in the absence of PINK1 simply because the mitochondrial network of PINK1 KO cells does not fragment., Supporting this hypothesis, we see that expression of constitutive active CaN promotes mitochondrial fragmentation in WT cells but not in PINK1 KO cells (Supplementary Fig. 13A, B). In addition, although PINK1 KO cells do not seem to have more elongated mitochondria than WT cells under basal condition, mitochondria do not fragment when treated with CCCP, indicating impaired fission in this condition (Supplementary Fig. 13A, B).

At the systemic level, CaN activation in PINK1 KO flies rescues the characteristic locomotor defects in climbing ability and mitochondrial dysfunction of these flies. Importantly, in the brain of the fly larvae, CaN expression enhances basal mitophagy even in the absence of PINK1 expression. This result highlights a fundamental difference between CaN-dependent mitophagy in MEFs compared to neuronal cells of the fly head: in MEFs, PINK1 expression seems to be an absolute prerequisite for mitophagy execution, presumably because PINK1 is required to promote Drp1-dependent fission. It is possible that in the neurons of the fly brain, mitophagic events predominately occur in a piece meal fashion, by selective targeting portions of mitochondria for turnover via mitochondria derived vesicles (MDV) [115, 116], a process that does not seem to require Drp1-dependent fission [115]. How mitochondrial autophagy is differentially executed in specific cell type and in the complexity of the whole organism remains poorly understood, and open to new discoveries.

As observed by others before us [93], we did not see defective mitophagy in the brain of PINK1 KO flies, raising questions regarding the physiological relevance of PINK1 activation in vivo during mitophagy. One possible explanation for this lack of effect might be that PINK1-independent mitophagic pathways are activated during development to compensate for PINK1 loss. Recent publications provide hints toward this hypothesis, and suggest that the mitochondrial quality control pathways are intertwined so that activation of one specific pathway can compensate for loss of another [117–119]. Nevertheless, PINK1 KO flies do present a very obvious mitochondrial phenotype: mitochondrial ultrastructure is deranged, Complex I activity is compromised, and mitochondrial respiration is defective [87–89, 92, 120]. These conditions increase the risk of oxidative stress and inflammation deriving from cytosolic release of mitochondrial DAMPs and exposure of mitochondrial antigens [121]. In this scenario, enhancement of PINK1-independent mitophagy can promote elimination of dysfunctional mitochondria, prevent ROS builds up, and mitigate inflammation. While we cannot exclude the possibility that CaN promotes pro-survival and neuroprotective pathways via functions that are independent of Parkin-driven mitophagy, our in vivo results fully support the hypothesis that the rescue depends at least in part on an amelioration of mitochondrial quality control, which in the fly head it occurs in the absence of PINK1.

**Fig. 11:**
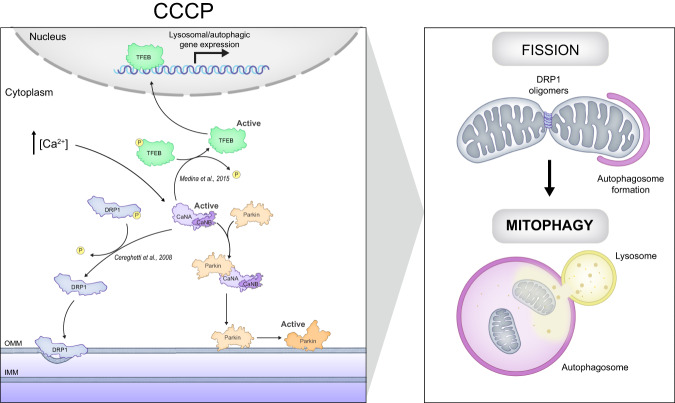
Schematic representation of the pathway regulating Parkin translocation and mitophagy induced by Calcineurin. Mitochondrial membrane potential drives PINK1 import into healthy mitochondria through the TOM and TIM complexes. Once on the IMM, PINK1 gets cleaved by MPP and PARL and eventually degraded by the ubiquitin-proteasome system [45, 131]. In this scenario, CaN is not active and Parkin is kept in the cytosol. Mitochondria depolarization induced by CCCP is followed by cytosolic Ca^2+^ rise [56, 132], which activates CaN [133]. Activated CaN promotes mitochondrial recruitment of Drp1 and Drp1-dependent mitochondrial fission by dephosphorylating Drp1 [51]. Activated CaN also promotes dephosphorylation of TFEB to induce its activation and the expression of autophagy and lysosomal genes [57]. In parallel, CaN interacts with Parkin and promotes Parkin translocation to mitochondria in a PINK1-independent fashion. The interaction between CaN and Parkin might be direct or via a binding partner.

In conclusion, this study highlights an unprecedented role for Ca^2+^-dependent phosphatase CaN in the regulation of Parkin translocation and mitophagy. A transient increase of cytosolic Ca^2+^ influx promotes activation of CaN, which interacts with Parkin and induces Parkin mitochondrial recruitment in a Miro1-dependent fashion, but independently of PINK1. In parallel, CaN activates the transcription of autophagy and lysosomal genes via TFEB [57], and allows Drp1 mitochondrial recruitment and mitochondrial fission to execute mitophagy (Fig. 11).

## Materials and Methods

### Cells

Mouse embryonic fibroblast cells (MEFs) and Human embryonic kidney 293T (HEK293T) were cultured in Dulbecco’s modified Eagle medium (DMEM) (Thermo Fisher Scientific) supplemented with 10% Fetal Bovine Serum (FBS), 2 mM L-glutamine, 1% MEM non-essential amino acids solution, 50 U/ml penicillin and 50 µg/ml streptomycin (Thermo Fisher Scientific),and incubated at 37 °C, in a humidified 5% CO2 atmosphere. PINK1 wildtype and knock-out cells were kindly provided by Prof. Francesco Cecconi lab (Danish Cancer Society, Denmark) and Prof. Juan Pedro Bolaños lab (Universidad de Salamanca, Spain).

Transfection was performed using Transfectin™ Lipid Reagent (BIO-RAD) following manufacturer instruction. 4–6 h after transfection the medium was changed and cells were processed for the indicated experiment 24/48 h after. This protocol has been used both for confocal microscope analysis and for protein assays. Alternatively, cells were transiently transfected with plasmid DNA using PEI (Polysciences, 24765) according to manufacturer’s instructions. Cells were silenced with Calcineurin siRNA oligo duplex (OriGene, SR416619) by direct transfection, using Transfectin (BIO-RAD) according to the protocol of the manufacturer. Cells were silenced with Miro1(Rhot1) siRNA Smartpool (Dharmacon, M-063998-01-0005) and PINK1 siRNA (Thermo Fisher Scientific, 1299001) by reverse transfection, using Lipofectamine 2000 (Thermo Fisher Scientific) according to the manufacturer’s protocol. siRNA transfected cells were collected after 48–72 h. Mitochondria fractions were isolated from HEK293T cells using the Qproteome Mitochondria Isolation Kit (Qiagen) following the manufacturer’s instructions.

The following drugs were used: CCCP 10 μM (Sigma-Aldrich), Rotenone 2 μM (Sigma-Aldrich), Oligomycin 1 mg/ml (Sigma-Aldrich), Antimycin A 2.5 μM (Sigma-Aldrich), FK506 0.6 μM (Sigma-Aldrich).

### Constructs and molecular biology

mCherry-Parkin and HA-Ubiquitin plasmids were obtained from Addgene. Site-directed mutagenesis, using QuickChange II XL kit (Agilent) and the following primers were used to generate a point mutation on Serine 65 in Parkin (S65E): F-MutpkSer65E (5’- GAC CTG GAT CAG CAG GCC ATT GTT CAC ATT GT- 3’) and R-MutpkSer65E (5’-ACA ATG TGA ACA ATG GCC TGC TGA TCC AGG TC- 3’). The same protocol was used for Ubiquitin point mutation at Serine65, and the following primers were used: F-MutUbSer65E (5’-ATC CAG AAG GAG GAG ACC CTG CAC CT- 3’) and R-MutUbSer65E (5’- AGG TGC AGG GTC TCC TCC TTC TGG AT- 3’). These constructs were named Parkin S65E and Ub-S65E.

Flag-tagged Parkin was inserted into pMSCV vector by using the pCR®8/GW/TOPO® TA Cloning® Kit (Thermo Fisher Scientific). To perform the pCR®8/GW/TOPO® cloning, Flag-Parkin construct was PCR amplified from pEYFP-C1-Parkin vector (available in the lab) using the following primers: Parkin-forward-BglII-Flag (5’ -AGCT AGATCT ATG GAT TAC AAG GAT GAC GAC GAT AAG ATG ATA GTG TTT GTC AGG- 3’) and EYFP-reverse (5’ -ACC ATG GTG AGC AAG GGC GAG- 3’).

pcDNA3.1-ΔCnA^H151Q^ (ΔCnA^H151Q^), pDCR-HA-ΔCnA (ΔCnA), pDCR-CnB, Drp1-YFP and variants, and mito-YFP were plasmids already available in the lab and described in [51, 122, 123]. pLVX-Puro-mitoKeima was kindly provided by Prof. Finkel (Center for Molecular Medicine, National Heart Lung and Blood Institute, NIH Bethesda, USA).

GFP-LAMP1 and GFP-Rab5 were purchased from Addgene.

### Production of lentiviral particles and infection

pLVX-Puro-mitoKeima was kindly provided by Toren Finkel (Center for Molecular Medicine, National Heart, Lung, and Blood Institute, NIH, Bethesda, MD, USA). HEK293T cells were seeded onto 100 mm-diameter tissue culture Petri dishes. 24 h after plating, cells were cotransfected using PEI with LV-mitokeima and the packaging plasmids pMDLg/pRRE, pRSV-Rev, pMD2.G. After 8 h, the transfection medium was replaced with fresh culture medium. 2 million MEFs were seeded onto 60 mm Petri dishes. After 48 h, the cells medium was collected and mixed with 6 μg/ml of Polybrene (Sigma-Aldrich). Receiver MEFs were infected for 36 h, before changing the medium. All procedures for the production and use of lentiviral particles were performed in a biosafety level-2 (P2) environment.

### Retroviral infection and generation of Parkin-flag stable cell lines

To generate Parkin-flag retroviruses, HEK 293 T cells were plated at 2 M cells/100-mm-diameter tissue culture dishes and transfected with the pN8E VSGV, Gag-pol packaging vectors and the retroviral vector (empty or containing Parkin-flag) by PEI (Polyscience) direct transfection. At 5 h post-transfection, the medium was replaced with fresh DMEM containing 10% FBS, and cells were grown for an additional 24 h, before the transfection was repeated. After 24 h, the conditioned medium containing recombinant retroviruses was collected and filtered through 0.45 μm-pore-size filters. Samples of these supernatants were applied immediately to MEFs cells, which had been plated 18 h before infection at a density of 10^5 cells/60-mm-diameter tissue culture dishes. Polybrene (Polyscience) was added to a final concentration of 6 μg/ml, and the supernatants were incubated with the cells for 24 h. After infection, the cells were placed in fresh growth medium and cultured in DMEM culture medium. Selection with 200 μg of Hygromycin B/ml (Sigma-Aldrich) was initiated 24 h after infection. After about 15 days, cells were expanded.

### Immunofluorescence (IF)

For Ubiquitin-TOM20 immunostaining, MEFs transiently expressing constitutively active CaN or corresponding empty vector were pretreated with 50 µM proteasome inhibitor Mg132 (Sigma-Aldrich) for 30 min. Cells were then fixed with 4% PFA in PBS for 15 min at room temperature, permeabilized with 0,1% Triton X-100 in PBS, and blocked with BSA 4% in PBS supplemented with 0,05% Tween20. Cells were incubated overnight with the following antibodies: Ubiquitin (1:100; Enzo Life Science; BML-PW8810) and TOM20 (1:200; Santa Cruz; sc-11415). Cells were washed three times in PBS supplemented with 0,05% Tween20 and subsequently incubated with the corresponding Alexa secondary antibodies (Thermo Fisher Scientific).

For GFP TAB2 NZF (k63 linked ubiquitin chain binding probe)-mitoRFP co-localization experiment, MEF cells stably expressing Parkin FLAG were plated on coverslips in 24 well plates in 500 ul of complete media, and transfected with mitoRFP, GFP TAB2 NZF, kindly provided by Prof. Ivan Dikic (Institute of Biochemistry II, Goethe University School of Medicine, Theodor-Stern-Kai 7, 60590 Frankfurt am Main, Germany) and either with empty vector (pcDDNA3.1+pDCR) or constitutive active CaN (CanB+CnA) by using Lipofectamine 2000. After 48 h of transfection one group was treated with 10□M of CCCP for 3 hr. After completion of the experiment, cells were rinsed with PBS and then fixed with 3.7–4% paraformaldehyde pH (7.4) for 20 min at room temperature, and permeabilised with 0.2% Triton X-100 in PBS for 15 min, blocked with 3% bovine serum albumin in PBST (PBS with 0.1% Triton X-100) for 30 min at room temperature, and subsequently incubated with anti-Parkin (1:200 Sc32282), and corresponding Alexa Fluor 647 conjugated secondary antibody (Thermo Fisher Scientific). High-resolution confocal 3D images (z steps 200 nm) were acquired sequentially using ZEN3 software on Laser scanning confocal microscope (Zeiss LSM 900 confocal microscope system) equipped with X100 /1.4 NA oil immersion objective with excitation laser 647, 561, and 488 and emission filters Alexa Fluor 647, Alexa fluor 568 and eGFP respectively. All images were processed with Fiji (National Institute of Health, Bethesda, MD). Colocalisation analysis was performed on stacked images with Coloc2 plugin using ROI, and percentage of colocalisation was calculated from Menders’ coefficients following Bolte S. et al. [124] Background subtraction was performed with rolling ball radius of 50 pixels before analysis.

### Imaging

For confocal imaging experiments of Parkin localization, transfected MEFs cells were seeded onto round glass coverslips. Cells were co-transfected with mCherry-Parkin and mito-YFP. When indicated, cells where cotransfected with CnB, the regulatory Calcineurin (Cn) domain, plus ΔCnA (constitutively active Cn) or ΔCnAH151Q (dominant negative mutant of Cn), and/or one of the Ubiquitin constructs (Ub or UbS65E). These constructs were then excited using 561 nm or 488 nm laser and using a *UPlanSApo 60x/1.35* objective (iMIC Andromeda) or Plan Apochromat 100x/1.4 objective (ZEISS LSM900 Airyscan2). Stack of images separated by 0.2μm along the z-axis were acquired. Image analysis was performed using ImageJ. The quantification was performed as calculation of the percentage of cells with Parkin puncta on mitochondria or through an ImageJ plugin for colocalization quantification (see the following paragraph for details).

### Image analysis using Squassh

To quantify Parkin colocalization with mitochondria, we created maximum-intensity projections of z-series with 0.2 µm increments. Quantification was then performed by using ‘Squassh’ (Segmentation and QUAntification of Subcellular SHapes), a plugin compatible with the imaging processing softwares ImageJ or Fiji, freely available from http://mosaic.mpi-cbg.de/?q=downloads/imageJ. Squassh is a segmentation method that enables both colocalization and shape analyses of subcellular structures in fluorescence microscopy images [61]. For Parkin-mitochondria colocalization analysis, segmentation was performed with the minimum intensity threshold for the first channel set to 0.35, for the second to 0.15 and the regularization weight to 0.015. Among the three different colocalization coefficients (C_signal_, C_number_ and C_size_), we preferentially used C_number_. It must be noted that Squassh-based analysis is unbiased as this method is completely automated and performed by computer software.

Colocalization analysis of Ubiquitin with TOM20 was performed on single plane images using Squassh. For this analysis, segmentation was performed with the minimum intensity threshold for the first channel set to 0.2, for the second to 0.2 and the regularization weight to 0.05. As colocalization coefficient we used C_number_.

### Mitokeima mitophagy analysis by flow cytometry

Mitokeima expressing MEFs were analyzed by flow cytometry (BD FACSAria™ sorter) as previously reported [125]. Cells were analyzed with flow cytometer equipped with a 405-nm and a 561-nm laser. Cells were excited with violet laser (405 nm) with emission detected at 610 ± 10 nm with a BV605 detector and with a yellow-green laser (561 nm) with emission detected at 610 ± 10 nm by a PE-CF594 detector simultaneously.

### Immunoblotting

At the established time points, the medium was removed and MEFs washed with ice-cold PBS. After withdrawing PBS, cells were scraped off the wells using a plastic cell scraper, they were resuspended in 1,5 ml of cold PBS and they were centrifuged at 3’000 g at 4 °C for 5 min. Supernatant was discarded and then the pellet was resuspended in an appropriate volume of radioimmunoprecipitation assay (RIPA) buffer (150 mM NaCl, 50 mM Tris-HCl, 1% NP-40, 0.25% Sodium Deoxycholate, 1 mM EDTA in distilled water and adjusted pH to 7.4) with freshly added protease inhibitors cocktail (PIC). Cells were kept on ice for 30 min. Lysate were cleared by centrifugation at 20’000 g for 15 min at 4 °C.

Protein concentrations of samples was determined using Pierce™ BCA Protein Assay Kit (Thermo Fisher scientific).

NuPAGE® LDS Sample Buffer (Invitrogen) and 2-Mercaptoethanol (Sigma-Aldrich) were mixed to samples and proteins were then denaturated at 95 °C for 15 min. Proteins were separated on ExpressPlus^TM^ PAGE gels (GenScript) and transferred to PVDF membrane (MERCK-Millipore). Membranes were incubated with indicated antibodies and imaged with ImageQuant LAS4000. Band densiometry quantification was performed using ImageJ software. The following antibodies were used: Actin (1:5000; Chemicon; MAB1501), ATP5A (1:5000; Abcam; ab14748), Calcineurin (1:1000; BD Bioscience; 556350 and 1:1000; Abcam; ab52761), His (1: 5000; Proteintech; 66005-1), Mfn1 (1:600; Proteintech; 13798-1-AP) RHOT1/MIRO1 (1:500, Abclonal; A22551), Parkin (1:1000, Santa Cruz; SC32282), Parkin (1:500, Abclonal; A0968), FLAG (1:3000; Cell Signalling; 2368 S), TOM20 (1:2000; Santa Cruz; sc-11415), VDAC (1:1000; Abcam; ab15895). Canonical secondary antibodies used were sheep anti-mouse or donkey anti-rabbit HRP (GE Healthcare). Mouse TrueBlot® ULTRA: Anti-Mouse Ig HRP (Rockland) was used for immunoprecipitation experiment.

### Thermal stability assay

Cells were plated onto 100 mm Petri dishes (10 M/dish). After 24 h, transfection was performed using PEI (Polyscience) with ΔCnA and CnB plasmids and the corresponding empty vectors. After 48 h from the transfection, cells were resuspended in PBS and snap-freezed in liquid nitrogen. The solution was aliquot into a PCR strip and incubated at the indicated temperatures for 3 min. The lysates were centrifuged at 16’000 g for 30 min at 4 °C. The soluble fraction was loaded into SDS-PAGE gel.

### In vitro interaction assay

Bacterial expression Parkin construct (NM_004562.2 GI:169790968) was transformed into BL21 DE3 E. Coli. Overnight culture inoculated from fresh colony was grown in Terrific broth media containing 2% glucose and 50 μg/ ml kanamycin at 37 °C. The following morning overnight cultures were diluted to OD 600 0.1 and continued shaking at 37 °C until OD 600 reached 0.8, flasks were transferred to 4 °C, cultures were then induced with 0.2 mM IPTG and expression was allowed to proceed overnight at 18 °C. Cells were harvested by centrifugation at 6’000 g for 15 min at 4 °C. For purification of Parkin construct, bacteria were resuspended in buffer A (50 mM Hepes pH 8.0, 200 mM NaCl, 10 mM imidazole, 250 μM TCEP and EDTA-free Complete protease inhibitor tablets (Roche)) and lysed using a French Press homogenizer. The lysate was cleared at 45’000 g for 25 min at 4 °C and the supernatant was loaded into HisPur NiNTA Chromatography 1 ml cartridge (Thermo Fisher Scientific). The resin was washed with 50 ml of buffer A containing 20 mM imidazole. IMAC purification was performed on an ÄKTA Purifier FPLC system (GE Healthcare) and 20 fractions were eluted in imidazole gradient (20 mM ÷ 500 mM) for 20 min. After elution, the protein was desalted using Vivaspin™ ultrafiltration spin columns into Buffer A without imidazole. The protein was then loaded onto a HiLoad Superdex 75 16/60 size exclusion chromatography column (GE Healthcare) that had been pre-equilibrated in Hepes 30 mM, NaCl 150 mM, Glycerol 10%. Collected fractions were then concentrated and loaded on Buffer A equilibrated NeutrAvidin agarose resin (Thermo Fisher) for 40 min at 4 °C. In parallel, CaN-flag expressing cells were lysed and protein extract was incubated with Parkin-bound resin overnight at 4 °C under gentle rotation. The resin was washed three times and boiled at 95 °C in 2X Laemmli buffer (160 mM Tris-HCl, pH 6.8, 4% SDS, 0.7 M Sucrose, 4% β-mercaptoethanol) in order to elute the protein sample and subsequently resolved it in a SDS-PAGE. As negative control for the assay we used USP14-Flag, a protein that is not supposed to interact with Parkin. USP14-Flag expressing cells were lysed and protein extract was incubated with Parkin-bound resin and processed as just described. As additional negative control, we immobilized MEF2D-His [126], a protein that is not supposed to interact with Calcineurin. CaN-Flag expressing cells were lysed and protein extract was divided in two fractions: first fraction was incubated with Parkin-bound resin, while the second fraction with MEF2D-bound resin overnight. Samples were processed as previously described.

### Proximity Ligation Assay (PLA)

For proximity ligation assay, we used a PLA kit from Sigma-Aldrich (Sigma-Aldrich DUO92014), and followed the manufacturer’s instructions. HeLa cells were grown on coverslips and transiently transfected with mCherry Parkin and PPP3CB-Flag using Trans-IT transfection reagent (Mirus Bio) for 18 h. They were then fixed with 4% paraformaldehyde (Electron Microscopy Sciences, Hatfield, Pennsylvania) for 10 min at room temperature. The blocking buffer (0.5% bovine serum albumin, 0.1% saponin, 50 mM NH_4_Cl in PBS) was then added to the cells for 20 min. The samples were washed in PBS and incubated overnight at 4 °C with the primary antibodies in the blocking buffer. The following antibodies were used for the PLA experiments: anti-GM130 (1:700; BD Biosciences); anti-PPP3CB (Origene, 1:200); anti-GRASP65 (1:1000) and anti-Parkin (1:1000) were from Abcam; Alexa 488-, Alexa 633- and Alexa 568-conjugated secondary antibodies (1:400) were from Invitrogen. As negative controls we used anti GRASP65, which is not supposed to interact with Parkin. As positive controls, we performed the PLA by using GRASP65 and GM130. The PLA was performed according to the manufacturer instructions. Finally, Hoechst was incubated for 10 min at room temperature. The coverslips were then mounted on glass microscope slides with Mowiol4-88 (Sigma-Aldrich). Immunofluorescence samples were examined using a confocal laser microscope (Zeiss LSM700 confocal microscope system; Carl Zeiss, Gottingen, Germany) equipped with × 63 1.4 NA oil objective. Optical confocal sections were taken at 1 Airy unit, with a resolution of 512 × 512 pixels or 1.024 × 1024 pixels. PLA analysis was performed using Fiji. Images were subjected to thresholding, and the number of particles (“PLA dots”) was calculated with the Analyze Particles function. As negative controls we used anti GRASP65, which is not supposed to interact with Parkin. As a positive control, we performed the PLA by using GRASP65 and GM130 (both expressed in the cis golgi network [77]).

### Co-Immunoprecipitation assay

MEFs and HEK293T cells were pretreated with 10 µM CCCP for 2 h and cell pellet was resuspended in ice-cold lysis buffer (Hepes 50 mM, Tween-20 0,1%, Triton X-100 1%, Glycerol 10% with freshly added phosphatase and protease inhibitor, pH 7.2). Cells were kept on ice for 30 min and the soluble fractions from cell lysates were isolated by centrifugation at 12,000 *g* for 15 min. In the meantime, 50 µl of Protein-A Agarose (Roche) were washed and equilibrated in lysis buffer. Protein extract was pre-cleared in the equilibrated resin with 30 min incubation at 4 °C. Pre-cleared sample was quantified using Pierce™ BCA Protein Assay Kit (Thermo Fisher Scientific). For co-immunoprecipitations, 5 mg of lysate was incubated with Parkin antibody (2 µg; Santa Cruz 32282) or with Calcineurin antibody (2 µg; BD Bioscience 556350). Anti-mouse IgG was used in both cases as negative control (2 µg; Santa Cruz sc-2025) with constant rotation overnight at 4 °C. Then, 50 μl of Protein-A beads (Roche) was added to lysates and incubated with rotation for 2 h at 4 °C. After incubation, the resin was washed three times in ice-cold PBS and the samples were eluted in Laemmli buffer with β-mercaptoethanol at 70 °C for 15 min and loaded on 8% SDS-PAGE.

### Real time PCR

Total RNA was extracted from the cells using ReliaPrep RNA Cell Miniprep isolation kit (Promega). RNA was quantified using Nanodrop spectrophotometer, 1 µg of total RNA was used to synthesize cDNA in 20 μl reaction mix using SensiFast cDNA synthesis kit (Meridian Life Science) according to the manufacturer’s protocol. HOT FIREPol SolisGreen qPCR mix (Solis BioDyne) was used for real-time PCR with the following conditions: 95 °C 10 min/40 cycles (95 °C 15 s, 60 °C 1 min). Dissociation curve was generated for checking the amplification specificity. The data were analyzed by comparative CT method to determine fold differences in expression of target genes with respect to the internal control. Target genes were amplified using the following primers: hPINK1-FW (5’- CGG ACG CTG TTC CTC GTT AT-3’), hPINK1-RW (5’- CGA TGC CCT GTT GAA CCA GA-3’), RHOT1(Miro1)-FW (5’- CTC CAC CTC AAG CCT TCA CTT G-3’), RHOT1(Miro1)-RW (5’- GCT CTT GAG GTC AGC TTG TGT C-3’), GAPDH FW (5’- GGC CAT CCA CAG TCT TCT G-3’), GAPDH RW (5’- TCA TCA GCA ATG CCT CCT G-3’), Actin FW (5’- GAT CAT TGC TCC TCC TGA GC-3’), Actin RW (5’- ACA TCT GCT GGA AGG TGG AC-3’).

### Electron microscopy

Samples were fixed with 2.5% glutaraldehyde in 0.1 M sodium cacodylate buffer pH 7.4 ON at 4 °C. The samples was postfixed with 1% osmium tetroxide plus potassium ferrocyanide 1% in 0.1 M sodium cacodylate buffer for 1 h at 4°. After three water washes, samples were dehydrated in a graded ethanol series and embedded in an epoxy resin (Sigma-Aldrich). Ultrathin sections (60-70 nm) were obtained with an Ultrotome V (LKB) ultramicrotome, counterstained with uranyl acetate and lead citrate and viewed with a Tecnai G2 (FEI) transmission electron microscope operating at 100 kV. Images were captured with a Veleta (Olympus Soft Imaging System) digital camera.

### Mitochondria membrane potential analysis

Mitochondria membrane potential was measured with the fluorescent dye Tetramethylrhodamine Methyl Ester (TMRM). MEFs cells were previously seeded on 24 mm round coverslips and transfected with ΔCnA + CnB plasmids or with the corresponding empty vectors. 48 h after transfection, cells were washed with HBSS containing 20 mM HEPES (pH 7.4). Cells were then incubated in HBSS with 1 µM Cyclosporin H and 10 nM TMRM for30 min at 37 °C. Cellular fluorescence images were acquired at room temperature with UPlanSApo 60x/1.35 objective (iMIC Andromeda). Sequential images were acquired every 1 min. After 5 min, 2.5 µg/ml Oligomycin was added directly into the acquisition chamber. After 30 min, mitochondria were fully depolarized by the addition of 2 µM of the protonophore carbonylcyanide-p-trifluoromethoxyphenyl hydrazone (FCCP) and recorded for further 5 min. Images were analyzed using Image J software. Mitochondria network of each cell was identified as region of interest (ROI) and field not containing cells was used as background. The mean fluorescence intensity was measured for each ROI. Following background substraction, each value of TMRM fluorescence was normalized with initial fluorescence and expressed as relative TMRM intensity.

### In vitro ubiquitination assay

In vitro ubiquitination assays were conducted as previously described [127] with little modification. Briefly, 2.4 to 3 × 10 ^5^ MEF cells were plated in 6 well in 2 ml complete media and co transfected with His-ubiquitin (pCI-HisUbi was a gift from Astar Winton), and either with empty vector or CaN using Lipofectamine 2000 (Invitrogen) in complete media. After 48 hr of transfection cells were treated with proteasome inhibitor Mg132 (30uM) for 4 h before being harvested and lysed in Lysis buffer (100 mM Na_2_ HPO_4_ /NaH_2_ PO_4_, 300 mM NaCl, 8 M Urea, 10 mM Tris (pH 8.0), 15 mM imidazole and 0.1% Tween 20) with addition of 20 uM N-Ethylmaleimide and 1X Halt Protease inhibitor cocktail (thermoscientific). Lysate was cleared by passing through 25 G needle and centrifuged for 20 min at 20000 *g* at 4 °C. Protein was quantified with Pierce BCA protein estimation kit and equal amount of protein was incubated with HisPur Ni-NTA Magnetic Beads (thermoscintific) in Binding buffer (Lysis buffer with 10 mM 2-mercapto-ethanol pH 8.0) for 4 h at room temperature. Beads were washed once with binding buffer and twice with Washing buffer (Binding buffer containing 30 mM imidazole pH 8.0). Proteins were eluted with Elution buffer (300 mM imidazole containing Binding buffer pH8.0, without tween-20), and subjected to western blotting analysis with indicated primary antibodies, anti-FLAG (3 ug/ml, Sigma-Aldrich F3165), anti-VDAC1/Porin (1:1000, Abcam Ab15895) anti-HA (1:1000, Cell Signaling Technology, C29F4), anti-Calcineurin A (1:1000, Abcam ab52761), anti ubiquitin (1:1000, ENZ-ABS840 Enzo) and anti GAPDH (1:5000, Sigma -Aldrich).

### Fly stocks and breeding conditions

Flies were raised on standard cornmeal medium and were maintained at 23 °C, 70% relative humidity, on a 12 h light: 12 h dark cycle.

We used ActGal4 or nSybGal4 standard lines crossed with w1118, generous gifts from Dr. Alexander Whitworth (University of Sheffield) as controls. PINK1B9 and PK OE lines were already described before [88, 89] and were a kind gift from Dr. Alexander Whitworth. Mito-QC line was also a kind gift from Dr. Alexander Whitworth [93]. CanA-14F line was described before [128] and was a kind gift by Dr Pascal Dijkers.

### Climbing assay

The climbing assay (negative geotaxis assay) was used to assess locomotor ability. Climbing data were obtained from groups of untreated wildtype, untreated PINK1B9, FK506-treated wildtype, and FK506-treated PINK1B9. Briefly, 10 flies for each strain were collected in a vertically-positioned plastic tube (length 12 cm; diameter 5 cm) with a line drawn at 6 cm from the bottom of the tube. Flies were gently tapped to the bottom of the tube, and the number of flies that successfully climbed above the 6-cm mark after 10 s was noted. Fifteen separate and consecutive trials were performed for each experiment, and the results were averaged. At least 30 flies were tested for each genotype or condition.

The number of flies that could climb unto, or above, this line within 10 or 20 s was recorded and expressed as percentage of total flies.

### Analysis of mitochondrial respiration in flies

The rate of mitochondrial O_2_ consumption was monitored using an Oxytherm System (Hansatech) with magnetic stirring and temperature control, maintained at 30 °C. Five adult male flies per genotype were homogenized in respiration buffer (120 mM sucrose, 50 mM KCl, 20 mM Tris-HCl, 4 mM KH2PO4, 2 mM MgCl2, 1 mM EGTA, 1 g/l fatty acid-free BSA, pH 7.2) and the following additions were made: proline 10 mM, glutamate 10 mM, malate 4 mM, ADP 2.5 mM, oligomycin 2 µM, CCCP 1.25 µM, antimycin 1.25 µM. O_2_ consumption was obtained from the registered slope of the graph. Respiratory control ratio (RCR), state III versus state IV (ADP-stimulated respiration over oligomycin-administered respiration), was also determined from the registered graphs. Data from 10–11 independent experiments were averaged.

### Analysis of mitophagy in flies (Mito-QC analysis)

Brains from third instar larvae were dissected in PBS and fixed in 4% formaldehyde, pH 7, for 20 min, rinsed in PBS and mounted in Prolong Diamond Antifade mounting medium (Thermo Fischer Scientific). Samples were generally dissected in the morning and imaged in the afternoon of the same day. Because X chromosome nondisjunction is present in multiple balanced PINK1B9 mutant stocks, correct genotypes were determined by PCR-based genotyping of discarded tissue after dissection.

Fluorescence imaging was conducted using a confocal microscope (Andromeda iMIC spinning disc live cell microscope, TILL Photonics, 60 × objective). Z-stacks were acquired at 0.2 µm-steps.

Confocal images were analyzed using Fiji (ImageJ) software. The mito-QC Counter plugin was used to quantify the number of mitolysosomes, according to Montava-Garriga et al [129].

### Statistical analysis

We used Origin 7.0 Professional or Prism 8 for statistical analysis. All data are expressed as mean ± SEM unless specified otherwise. Statistical significance was measured by an unpaired t-test or one-way or two-way ANOVA followed by ad hoc multiple comparison test. *p*-values are indicated in the figure legend. Data information: n = number of biological replicate; *P ≤ 0.05, **P ≤ 0.01, ***P ≤ 0.001, ****P ≤ 0.0001.

### Supplementary information

Supplementary figure legends and supplementary figures

Figure Supplementary 1

Figure Supplementary 2

Figure Supplementary 3

Figure Supplementary 4

Figure Supplementary 5

Figure Supplementary 6

Figure Supplementary 7

Figure Supplementary 8

Figure Supplementary 9

Figure Supplementary 10

Figure Supplementary 11

Figure Supplementary 12

Figure Supplementary 13

Final Supplementary western blot uncrop

checklist

Related Manuscript File

## Data Availability

We confirm that all relevant data are available from the authors. Full and uncropped western blots are uploaded as ‘Supplemental Material’ (final supplementary figure).
