# Supplementary material for: Activation of Ca2+ phosphatase Calcineurin regulates Parkin translocation to mitochondria and mitophagy in flies
Source: Cell Death Differ. 2024 Jan 18;31(2):217–38. doi: 10.1038/s41418-023-01251-9 (PMC10850161; doi:10.1038/s41418-023-01251-9)

Activation of Ca^{2+} phosphatase Calcineurin regulates Parkin translocation to mitochondria and mitophagy in flies.

Elena Marchesan[1], Alice Nardin[1], Sofia Mauri[1], Greta Bernardo[1], Vivek Chander[1], Simone Di Paola[2,3], Monica Chinellato[1], Sophia von Stockum[1], Joy Chakraborty[1], Stephanie Herkenne[1], Valentina Basso[1], Emilie Schrepfer[1,4], Oriano Marin[5], Laura Cendron[1], Diego L. Medina[2,6], Luca Scorrano[1,4] and Elena Ziviani[1*]

[1]Department of Biology, University of Padova, Padova, Italy.

[2]Telethon Institute of Genetics and Medicine (TIGEM), Pozzuoli, Naples, Italy.

[3]current address: Institute for Experimental Endocrinology and Oncology (IEOS), National Research Council (CNR), Napoli, Italy

[4]Dulbecco-Telethon Institute, Venetian Institute of Molecular Medicine (VIMM), Padova, Italy

[5]Department of Biomedical Sciences (DSB), University of Padova, Padova, Italy.

[6]Medical Genetics Unit, Department of Medical and Translational Science, Federico II University, Naples, Italy

*Correspondence should be addressed to E.Z. (e-mail elena.ziviani@unipd.it)

SUPPLEMENTARY INFORMATION

Supplementary Figure 1: Parkin translocation to mitochondria is regulated by Calcineurin

Representative confocal images of wild type MEFs transfected with mCherry-Parkin and mito-YFP for 2 days before being treated with 40 μM BAPTA for 30 min or with 40 μM BAPTA for 30 min prior to 3 hrs 10μM CCCP treatment, as indicated. Graph bar shows mean±SEM of percentage of cells with mCherry-Parkin on mitochondria. Student's test ($n=3$; $p<0.01$).

Supplementary Figure 2: Parkin translocation to mitochondria is regulated by Calcineurin

Representative confocal images of wild type MEFs transfected with mCherry-Parkin and mito-YFP for 2 days before being treated with 0.6 μM FK506 for 30 min or with 0.6 μM FK506 for 30 min prior to 3hrs/10μM CCCP treatment, as indicated. Graph bar shows mean±SEM of percentage of cells with mCherry-Parkin on mitochondria for at least ≥ 300 cells per biological replicate. Student's test (n=3; $p<0.01$).

Supplementary Figure 3: Parkin translocation to mitochondria is regulated by Calcineurin

Graph bar shows mean±SEM of percentage of cells with mCherry-Parkin on mitochondria for at least ≥ 300 cells per biological replicate. Cells were transfected with mCherry-Parkin and after 2 days they were treated as indicated. One-way ANOVA followed by Tukey's multiple comparison test (n=3; $p<0.01$).

Supplementary Figure 4: Parkin translocation to mitochondria is regulated by Calcineurin

Western blot analysis of protein lysates extracted from MEFs downregulating Calcineurin and relative control. Cells were treated with CaN siRNA and control siRNA, protein lysates were collected after 2 days and subjected to Western blotting analysis with the indicated antibodies.

Supplementary Figure 5: Parkin translocation to mitochondria is regulated by Calcineurin

(A) Representative confocal images of wild type MEFs transfected with mCherry-Parkin, mito-YFP and with empty vector (EV) or constitutively active CaN (DCnA) for 2 days before being treated with DMSO as control or 10 μM CCCP for 3hrs.

(B) Quantification of A. Graph bar shows mean±SEM of percentage of cells with mCherry-Parkin on mitochondria for at least ≥ 300 cells per biological replicate. Two-way ANOVA followed by Tukey's multiple comparison test (n=3-9; $p<0.001$).

(C) Quantification of A by using Squassh. The graph bars show mean±SEM of Squassh colocalization coefficient for at least ≥ 50 images per biological replicate. 0=no colocalization, 1=perfect colocalization. Two-way ANOVA followed by Tukey's multiple comparison test (n=3-4; $p<0.01$).

Supplementary Figure 6: Parkin translocation to mitochondria is regulated by Calcineurin

(A) Western blot analysis of cytosolic and mitochondria fractions isolated from HEK293T cells upon PINK1 downregulation (PINK1 siRNA) and relative control (Ctrl siRNA), expressing empty vector (EV) or constitutively active CaN (ΔCnA). (B) Real-time PCR analysis of mRNA extracted from HEK293T cells downregulating PINK1 and relative control. Cells were treated with PINK1 siRNA and control siRNA, mRNAs were collected after 3 days and subjected to Real-time PCR with the indicated primers.

Supplementary Figure 7: Expression of constitutive active Calcineurin does not affect mitochondria membrane potential

Analysis of mitochondria membrane potential upon expression of constitutive active CaN. MEFs cells were incubated in presence of 10nM TMRM, and images were acquired with confocal microscope. Changes of mitochondria TMRM fluorescence (expressed as the % of initial fluorescence) were followed over time.

Supplementary Figure 8: Parkin translocation induced by Calcineurin is Miro1-dependent

(A) Western blot analysis of protein lysates extracted from MEFs downregulating Miro1 and relative control. Cells were treated with Miro1 siRNA and control siRNA, protein lysates were collected after 3 days, and subjected to Western blotting analysis with the indicated antibodies. (B) Real-time PCR analysis of mRNA extracted from MEFs downregulating Miro1 and relative control. Cells were treated with Miro1 siRNA and control siRNA, mRNA were collected after 3 days and subjected to Real-time PCR with the indicated primers.

Supplementary Figure 9: Calcineurin interacts with Parkin

(A) Representative images of HeLa cells transfected with mCherry-Parkin and probed with antibody against Calcineurin (PPP3CB). Cells were fixed, permeabilized and incubated with the indicated primary antibody, corresponding fluorophore-conjugated secondary antibody, and DAPI for nuclear staining.

(B) Representative images of HeLa cells transfected with Parkin-YFP probed with antibodies against GRASP65 and Parkin. Cells were fixed, permeabilized and incubated with the indicated primary antibodies, corresponding fluorophore-conjugated secondary antibodies, and Hoechst for nuclear staining. GRASP65 is a *cis*-Golgi resident protein and does not interact with Parkin.

(C) Representative images of HeLa cells probed with antibodies against GRASP65 and GM130. Cells were fixed, permeabilized and incubated with the indicated primary antibodies, corresponding fluorophore-conjugated secondary antibodies, and Hoechst for nuclear staining. GRASP65 is a peripheral membrane protein that resides in the *cis*-Golgi apparatus and interacts with GM130, which is also expressed in the *cis*-Golgi network.

Supplementary Figure 10: Calcineurin interacts with Parkin

HEK 293T cells were treated with CCCP-2hrs and subjected to immunoprecipitation (IP) of CaN using anti-CaN antibody. Western Blot analysis was performed with antibody anti-Parkin on the pulled down samples. Inputs represent 5% of the protein lysates and IP eluate 100% of the protein lysates.

Supplementary Figure 11: CCCP-induced mitophagy is impaired in cells downregulating Calcineurin

Representative scatterplots depicting the mean relative level of global mt-Keima signal in CaN downregulating cells and relative control.

Supplementary Figure 12: Calcineurin requires PINK1 to induce mitophagy in MEFs

(A) Representative Western blot analysis of protein lysates extracted from PINK1 WT and KO MEFs. Cells were transfected with constitutive active CaN (ΔCnA) and after 2 days they were treated with 10 μM CCCP for the indicated time.

(B) Quantification of (A). Line chart shows mean±SEM of ATP5A protein level normalized to Actin. Student's t-test (n=5; $p<0.05$).

(C) mt-Keima analysis of wild type (WT) and PINK1 KO MEFs. Cells were transfected with constitutive active CaN (ΔCnA) or empty vector (EV) for 2 days before being treated with DMSO or 10 μM CCCP for 3hrs, and subjected to FACS analysis. Two-way ANOVA followed by Tukey's multiple comparison test (n=6; $p<0.01$).

Supplementary Figure 13: Calcineurin requires PINK1-mediated DRP1 mitochondrial fission to induce mitophagy in MEFs

(A) Representative confocal images of cells of the indicated genotype transfected with mito-YFP and constitutive active CaN (DCnA) or empty vector (EV), treated with DMSO or 10μM CCCP for 3hrs.

(B) Quantification of (A) using Squassh. The graph bar shows mean ± SEM of mean object length of each image for at least ≥ 70 images per biological replicate. Two-way ANOVA followed by Tukey's multiple comparison test (n=3; $p<0.05$).

Supplementary Figure 1

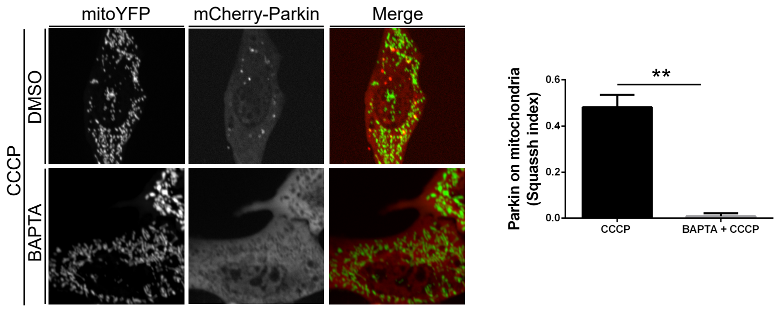

Supplementary Figure 2

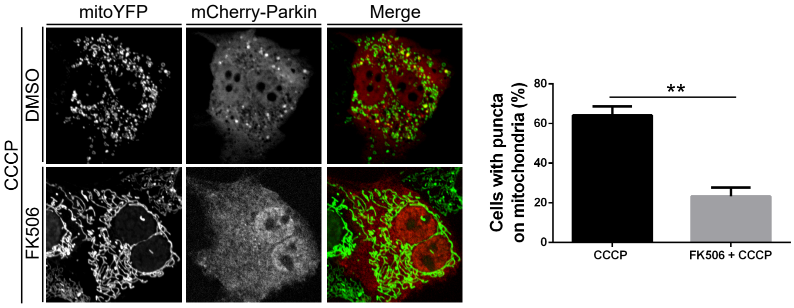

Supplementary Figure 3

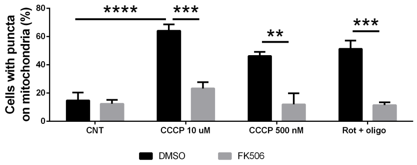

Supplementary Figure 4

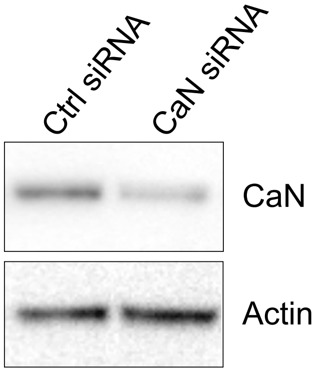

Supplementary Figure 5

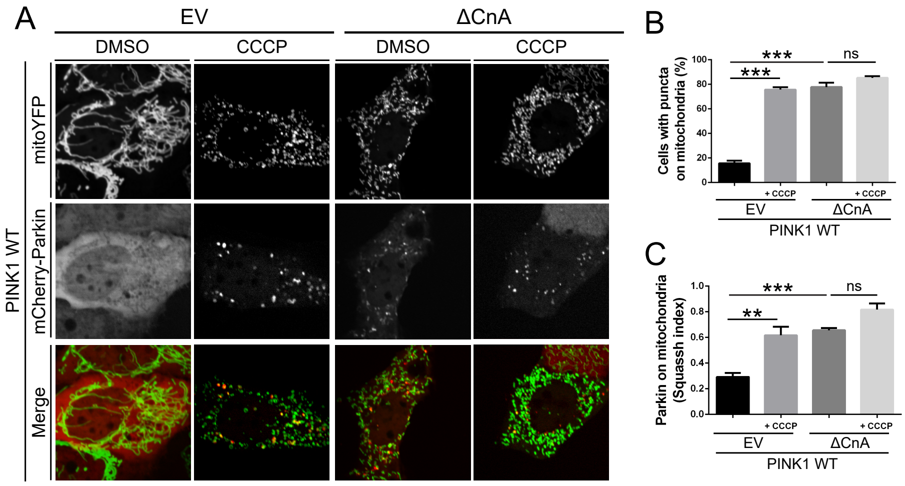

Supplementary Figure 6

A

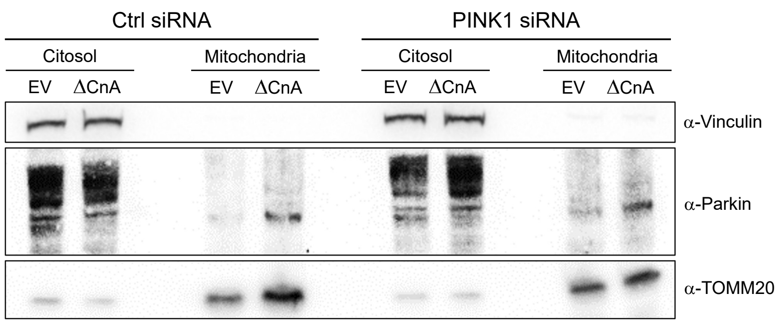

B

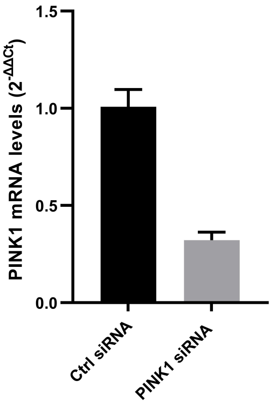

Supplementary Figure 7

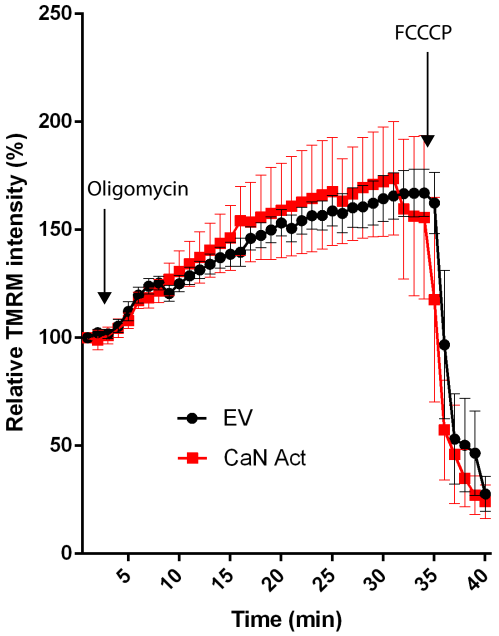

Supplementary Figure 8

A

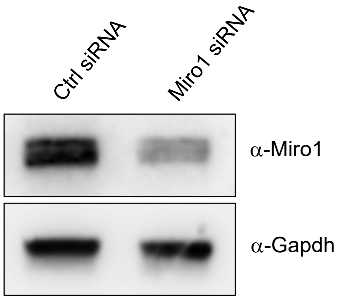

B

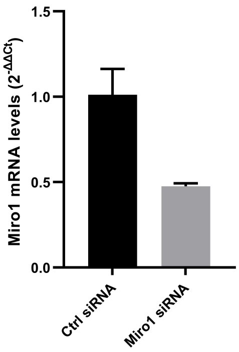

Supplementary Figure 9

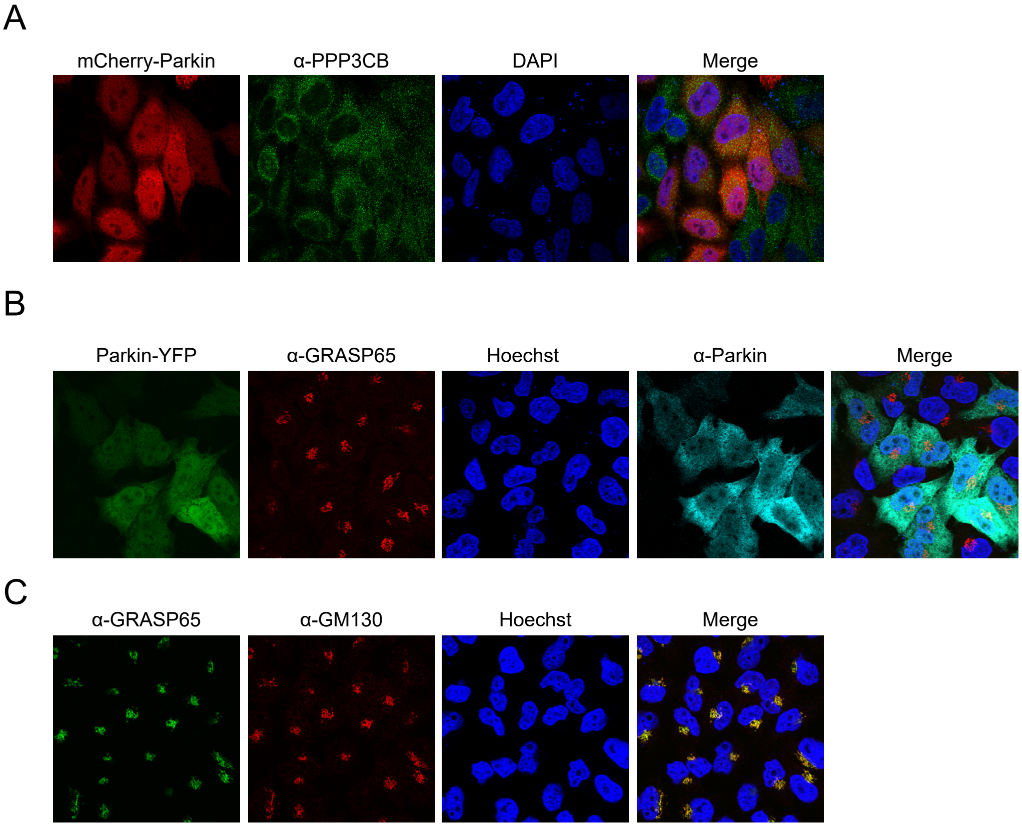

Supplementary Figure 10

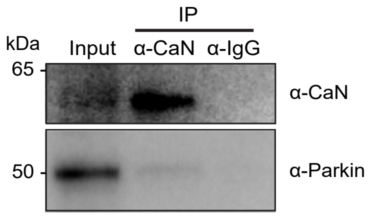

Supplementary Figure 11

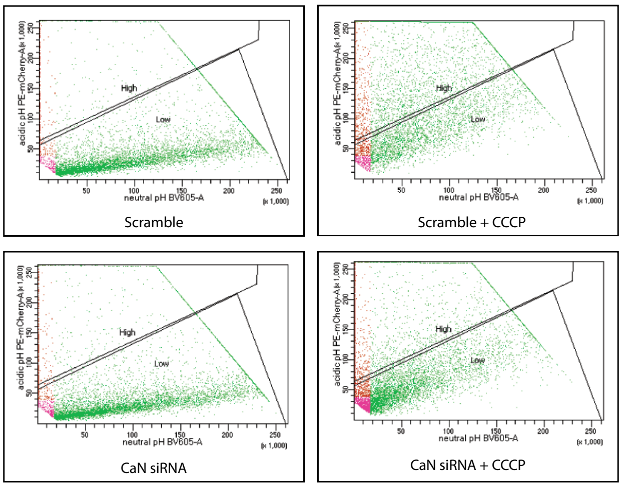

Scramble | Scramble + CCCP | CaN siRNA | CaN siRNA + CCCP

Supplementary Figure 12

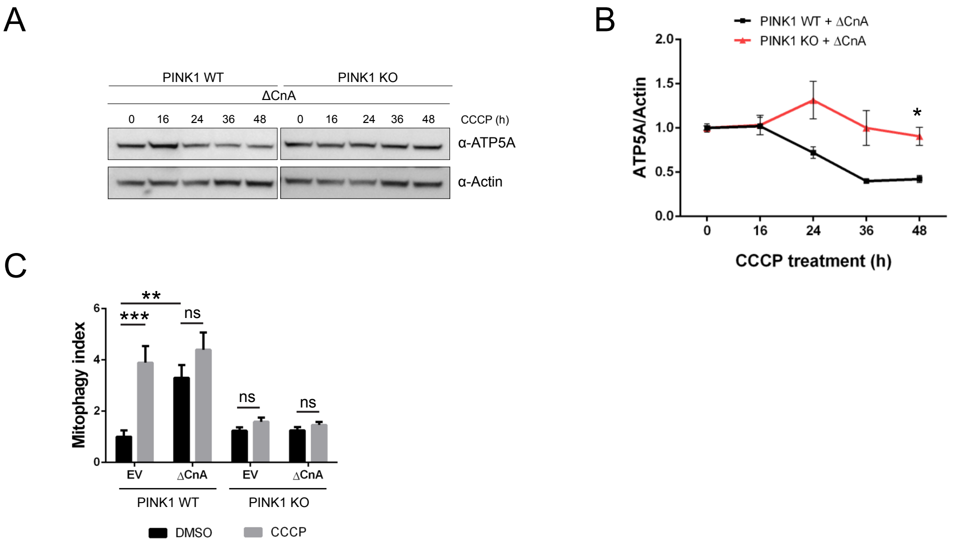

Supplementary Figure 13

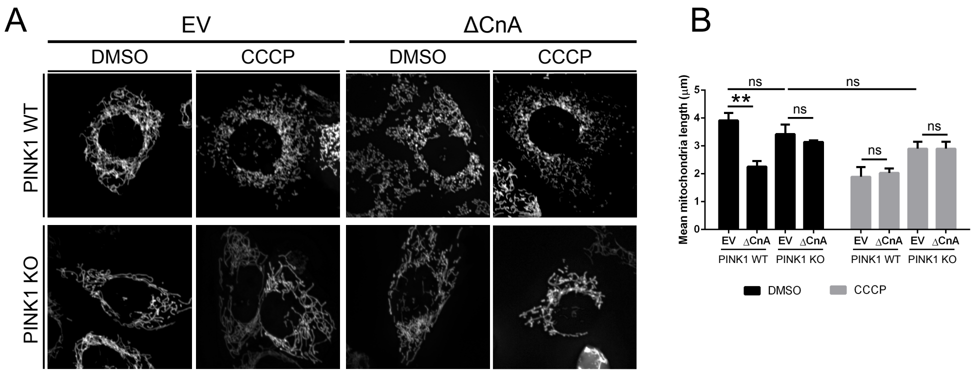

Supplement: Supplementary file 1 — Supplementary figure legends and supplementary figures [file 41418_2023_1251_MOESM1_ESM.pdf]
