# Supplementary figures and images for: Activation of Ca2+ phosphatase Calcineurin regulates Parkin translocation to mitochondria and mitophagy in flies
Source: Cell Death Differ. 2024 Jan 18;31(2):217–38. doi: 10.1038/s41418-023-01251-9 (PMC10850161; doi:10.1038/s41418-023-01251-9)

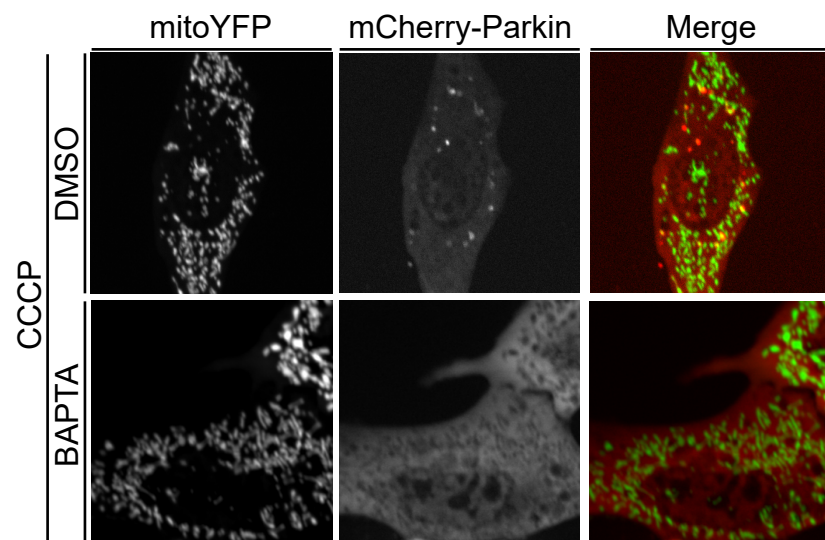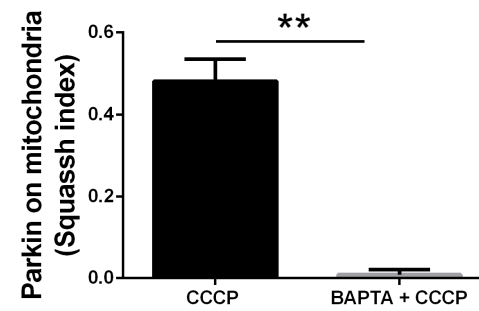

Supplement: Supplementary file 2 — Figure Supplementary 1 [file 41418_2023_1251_MOESM2_ESM.pdf]

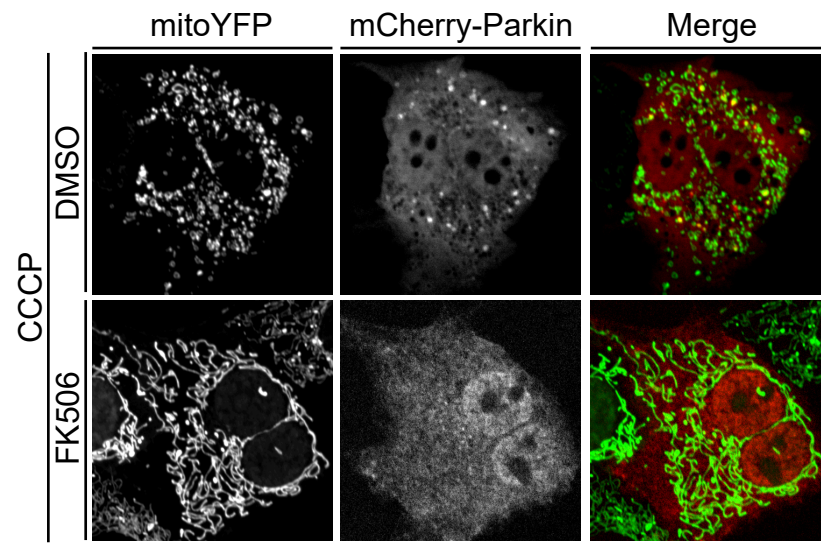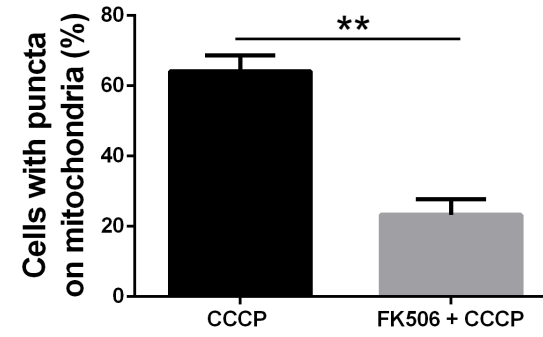

Supplement: Supplementary file 3 — Figure Supplementary 2 [file 41418_2023_1251_MOESM3_ESM.pdf]

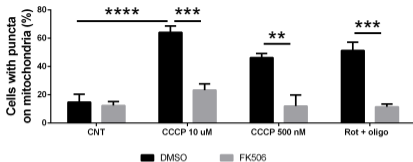

Supplement: Supplementary file 4 — Figure Supplementary 3 [file 41418_2023_1251_MOESM4_ESM.pdf]

Ctrl siRNA

CaN siRNA

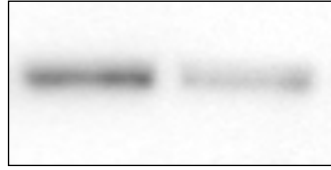

CaN

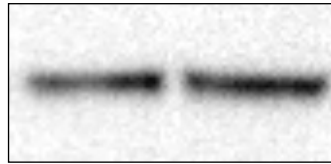

Actin

Supplement: Supplementary file 5 — Figure Supplementary 4 [file 41418_2023_1251_MOESM5_ESM.pdf]

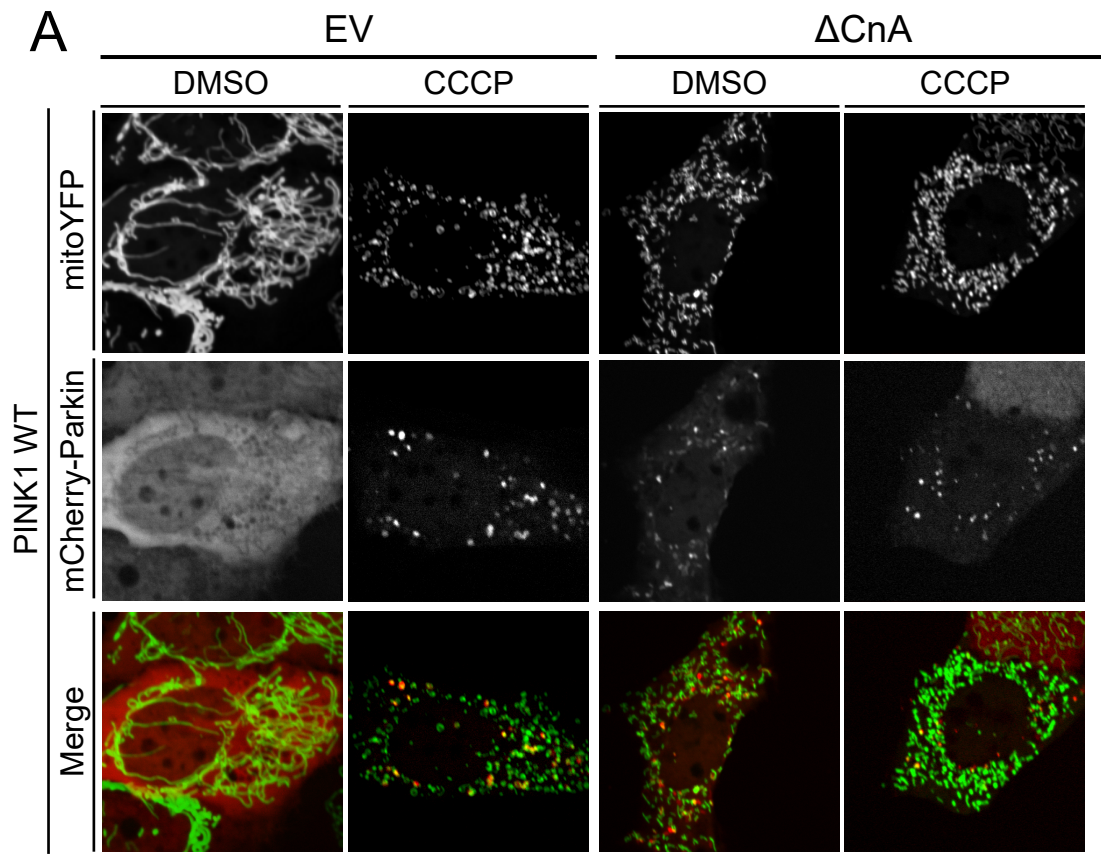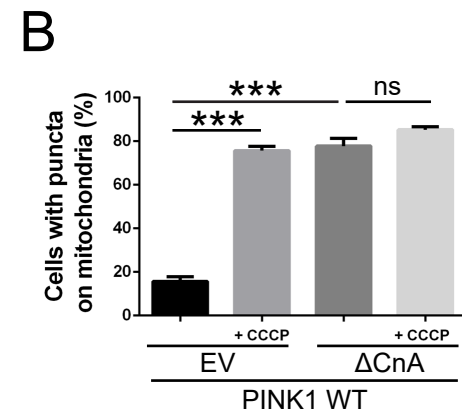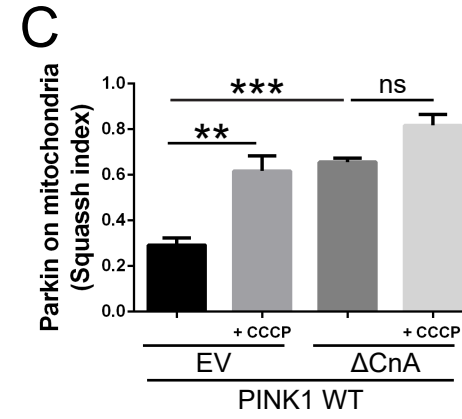

Supplement: Supplementary file 6 — Figure Supplementary 5 [file 41418_2023_1251_MOESM6_ESM.pdf]

A

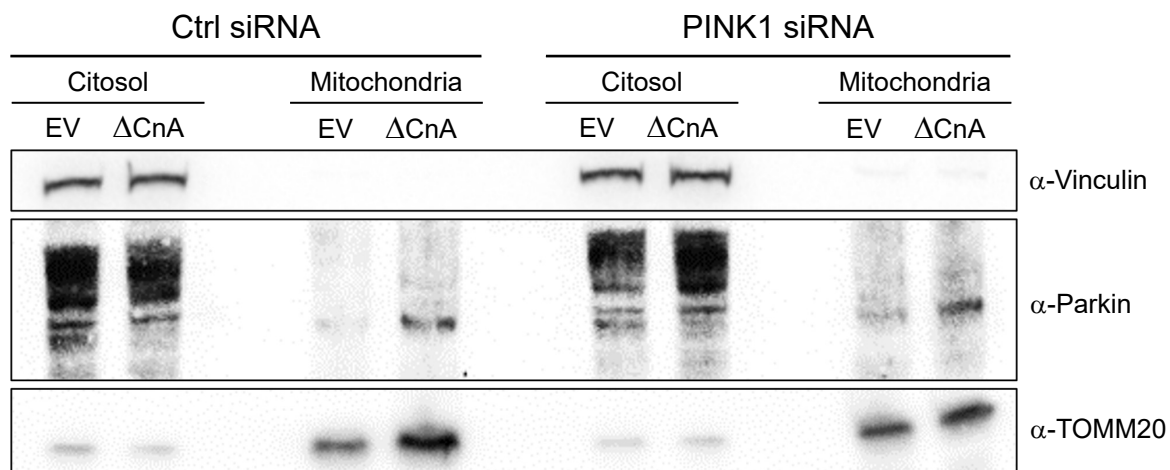

B

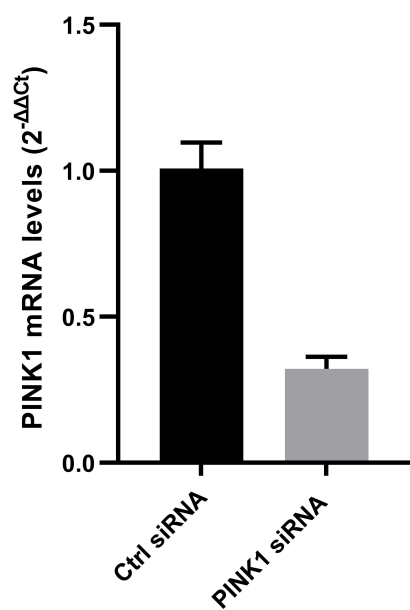

Supplement: Supplementary file 7 — Figure Supplementary 6 [file 41418_2023_1251_MOESM7_ESM.pdf]

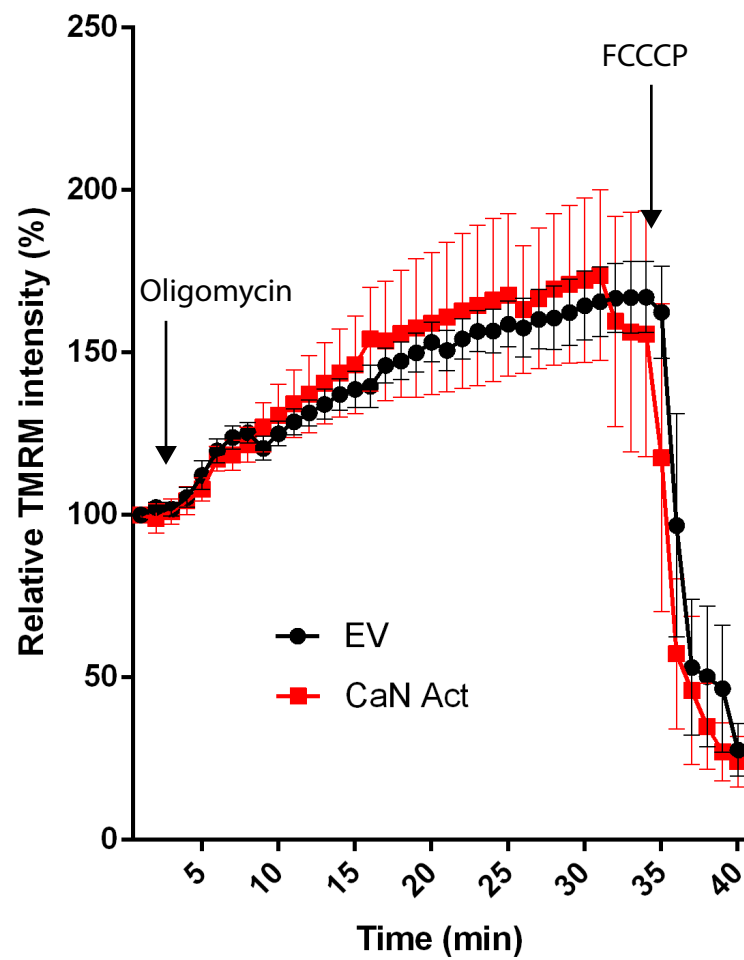

Supplement: Supplementary file 8 — Figure Supplementary 7 [file 41418_2023_1251_MOESM8_ESM.pdf]

A

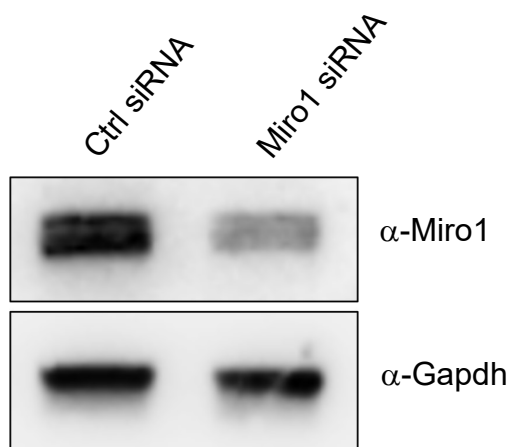

B

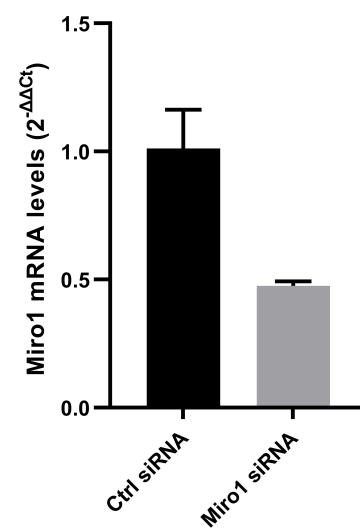

Supplement: Supplementary file 9 — Figure Supplementary 8 [file 41418_2023_1251_MOESM9_ESM.pdf]

A

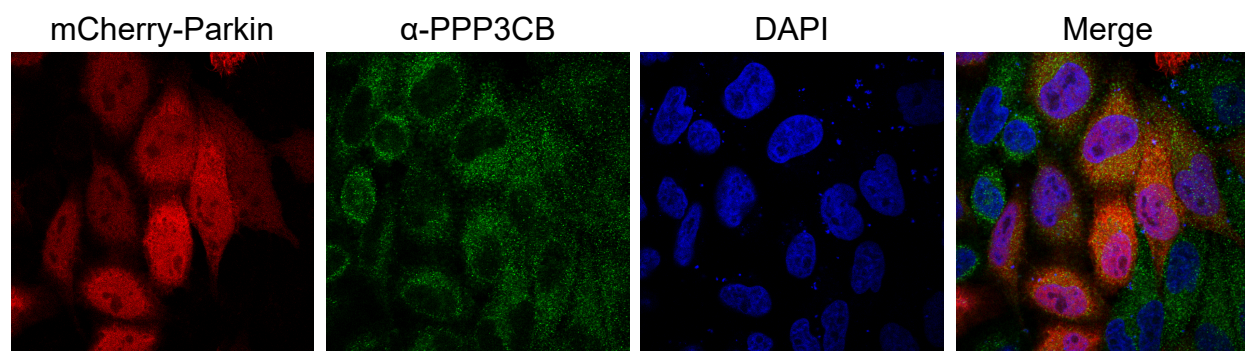

B

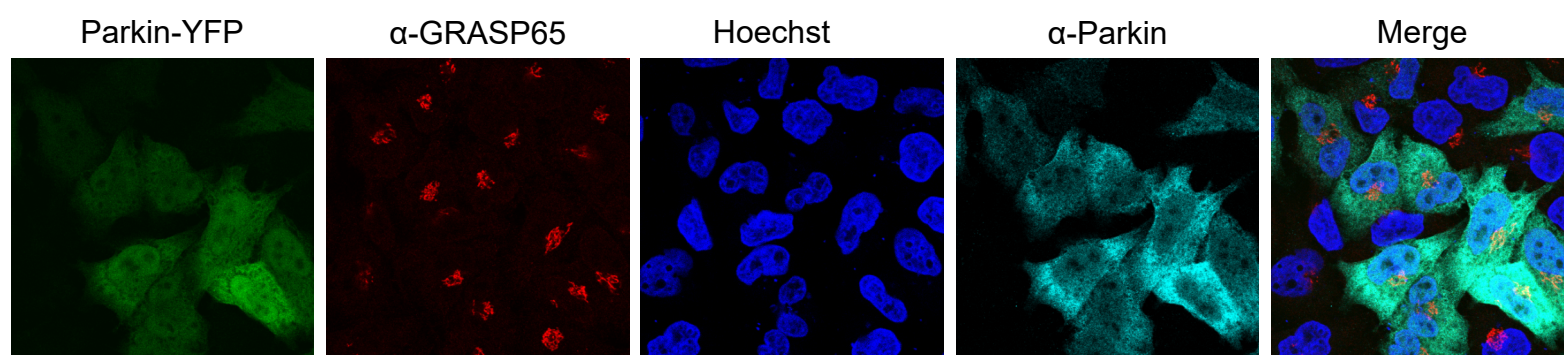

C

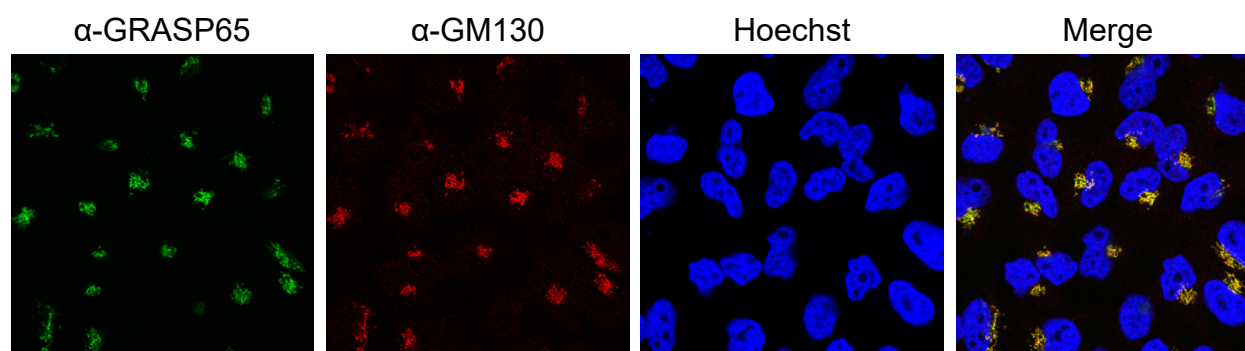

Supplement: Supplementary file 10 — Figure Supplementary 9 [file 41418_2023_1251_MOESM10_ESM.pdf]

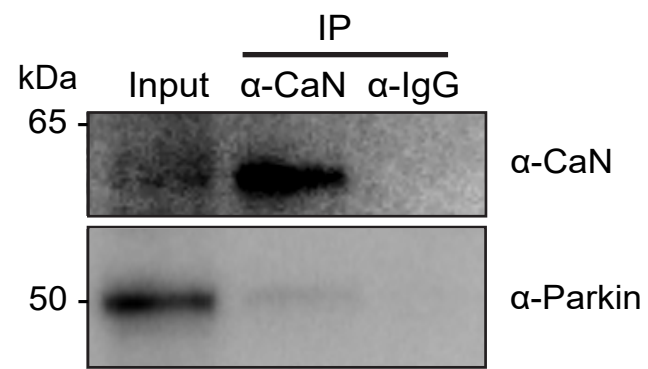

Supplement: Supplementary file 11 — Figure Supplementary 10 [file 41418_2023_1251_MOESM11_ESM.pdf]

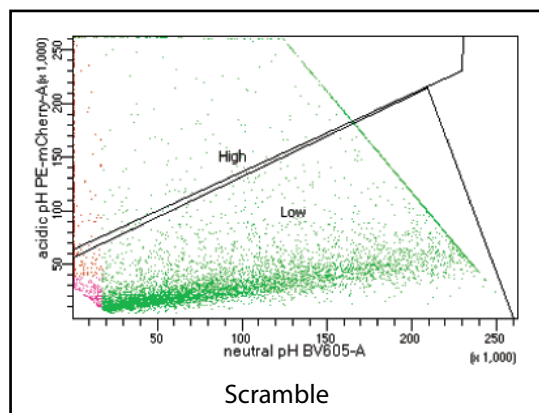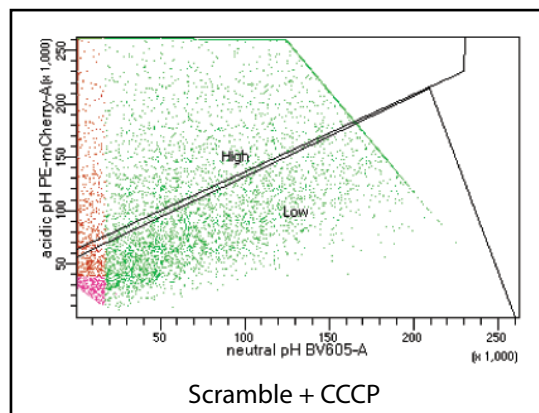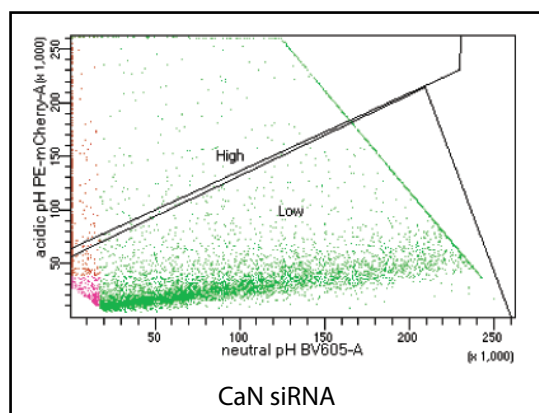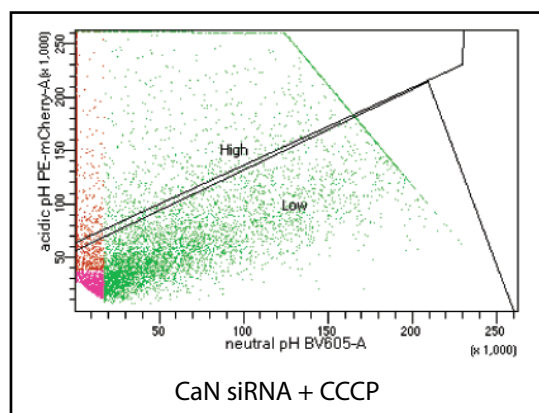

Supplement: Supplementary file 12 — Figure Supplementary 11 [file 41418_2023_1251_MOESM12_ESM.pdf]

A

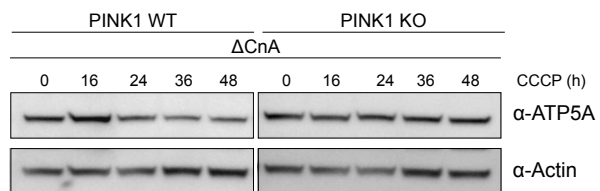

B

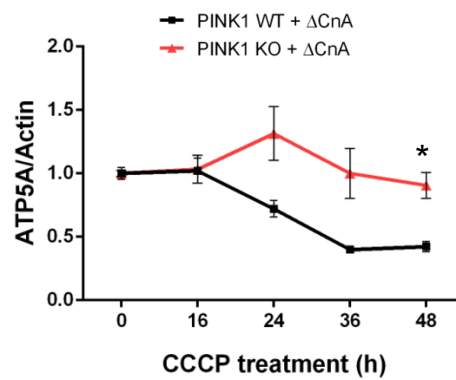

C

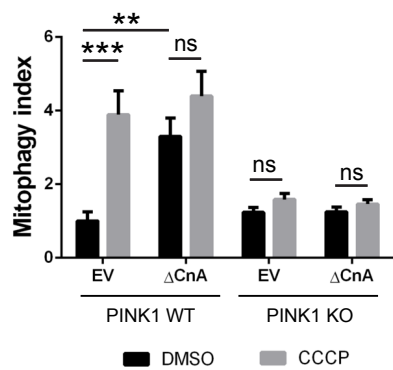

Supplement: Supplementary file 13 — Figure Supplementary 12 [file 41418_2023_1251_MOESM13_ESM.pdf]

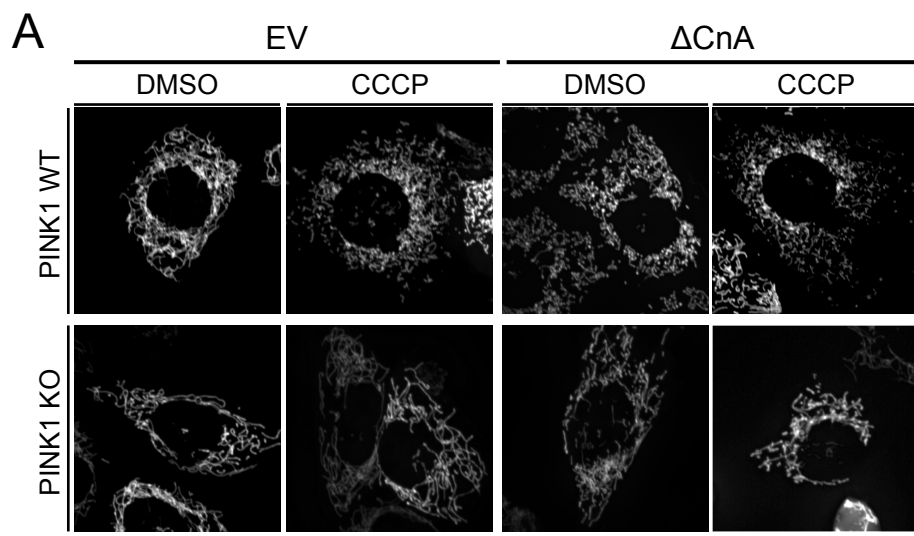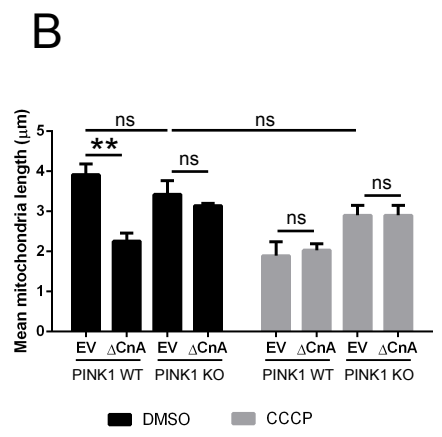

Supplement: Supplementary file 14 — Figure Supplementary 13 [file 41418_2023_1251_MOESM14_ESM.pdf]
