# Supplementary material for: Activation of Ca2+ phosphatase Calcineurin regulates Parkin translocation to mitochondria and mitophagy in flies
Source: Cell Death Differ. 2024 Jan 18;31(2):217–38. doi: 10.1038/s41418-023-01251-9 (PMC10850161; doi:10.1038/s41418-023-01251-9)

FIGURE 3G

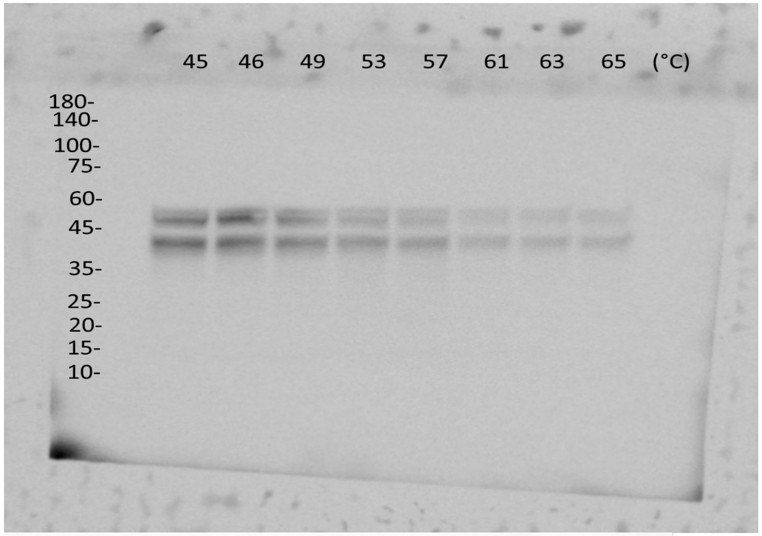

α-Parkin 1:1000
Santa Cruz Biotechnology
sc-32282

17/2/2017

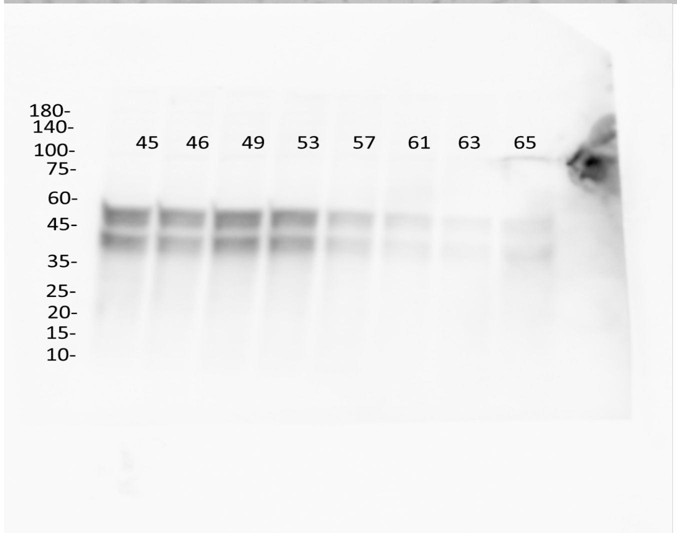

α-Parkin 1:1000
Santa Cruz Biotechnology
sc-32282

17/2/2017

FIGURE 3I

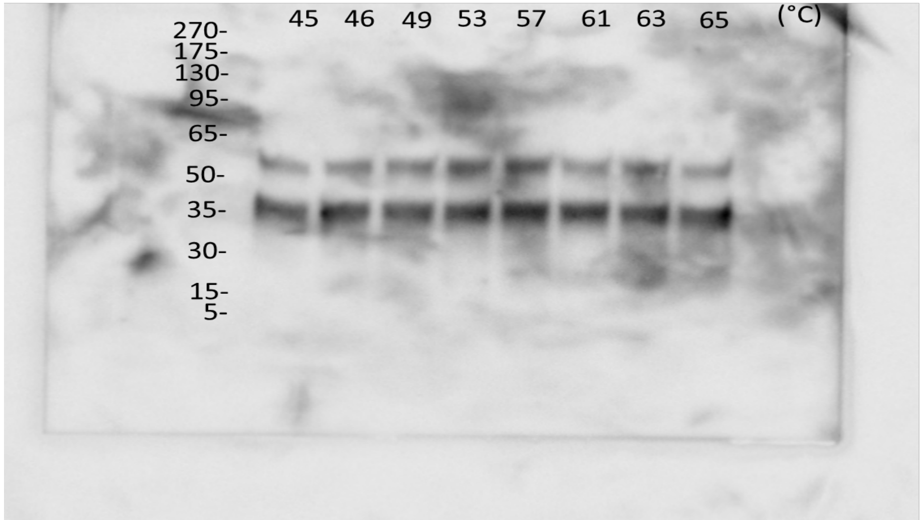

α-Parkin 1:1000
Santa Cruz Biotechnology
sc-32282

16/12/2019

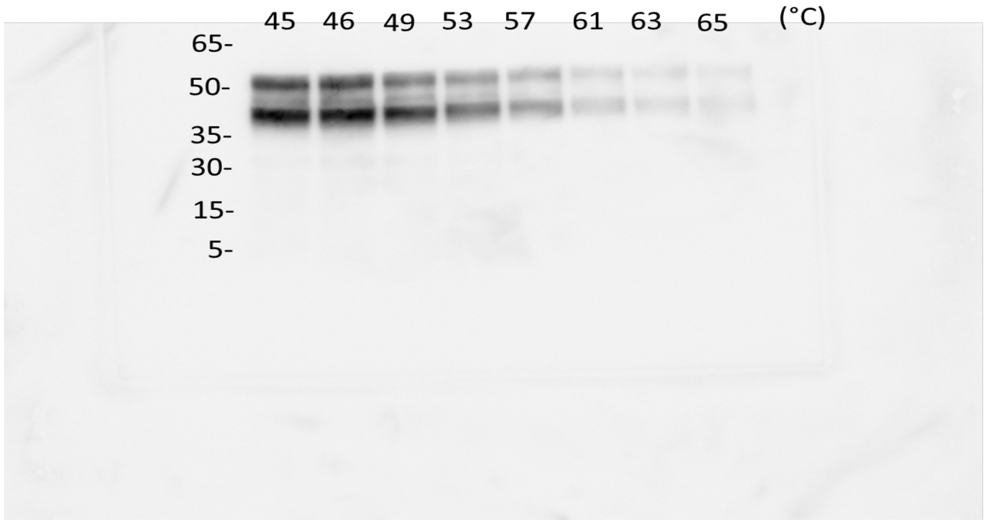

α-Parkin 1:1000
Santa Cruz Biotechnology
sc-32282

17/11/2019

FIGURE 5B

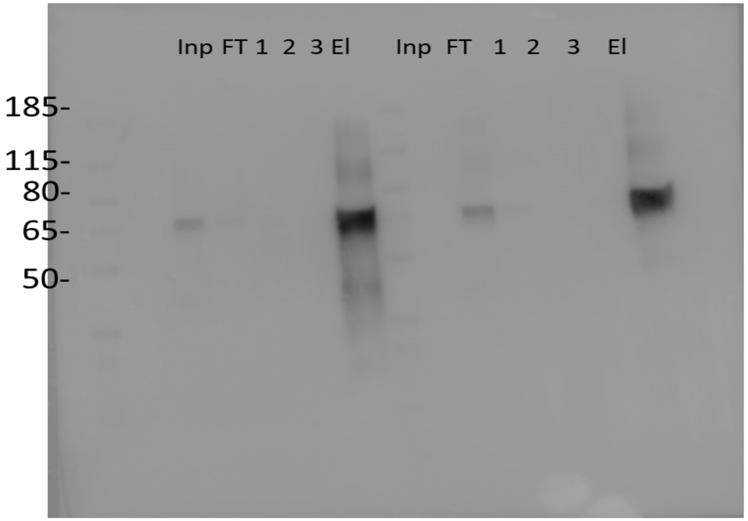

α – Parkin 1:1000
Santa Cruz SC-32282

18/11/21

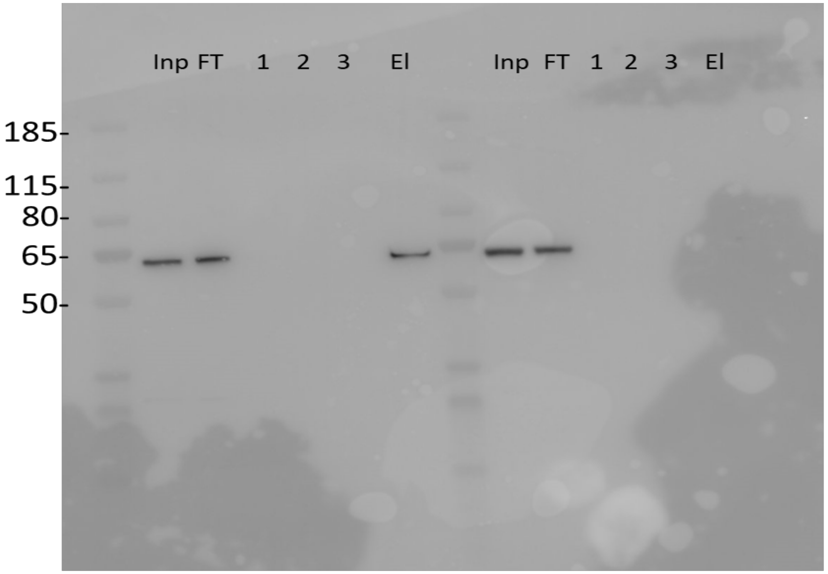

α – FLAG 1:10000
Sigma A8592

18/11/21

Legend
Inp: input represent 5% protein lysate of CaN-flag/USP14-FLAG expressing cells
FT: Flow through represents the protein fraction not bound to the beads
1,2,3: Three washes
EL: Elution of protein bound to the beads

FIGURE 5B

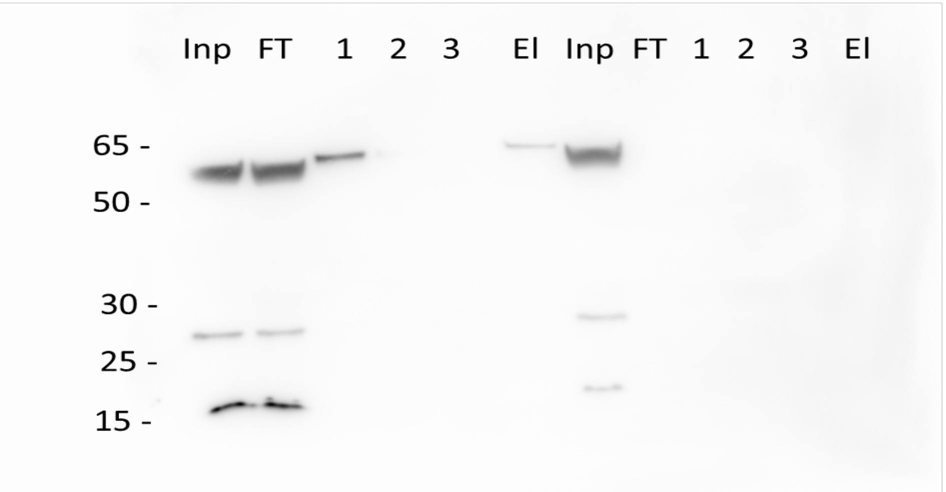

α – Flag 1:1000
Sigma A8592

6/12/21

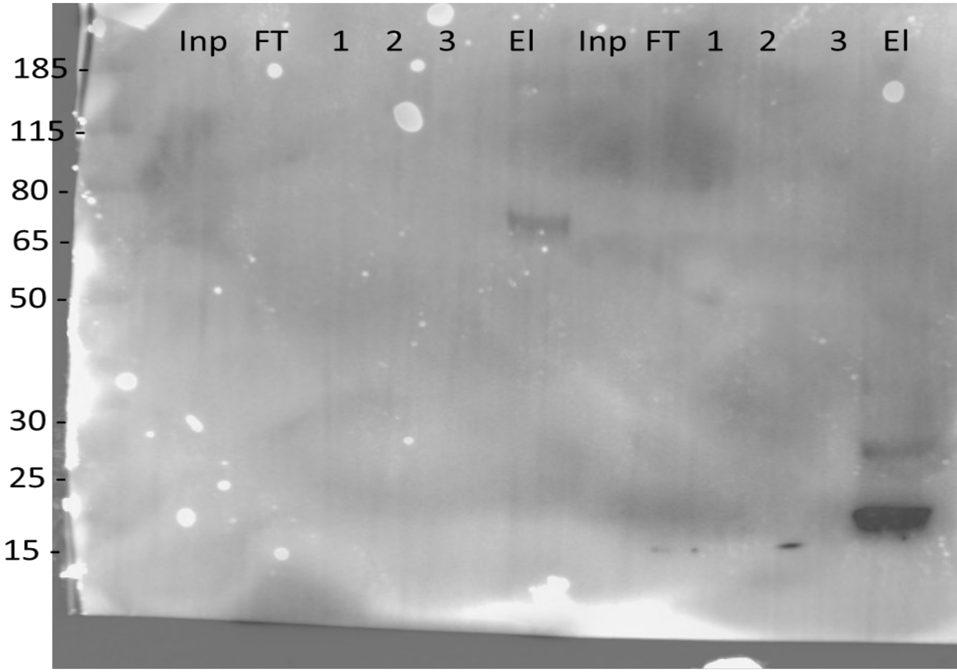

α – HIS 1:1000

6/12/21

Legend
Inp: input represent 5% protein lysate of CaN-flag expressing cells
FT: Flow through represents the protein fraction not bound to the beads
1,2,3: Three washes
EL: Elution of protein bound to the beads

FIGURE 5E

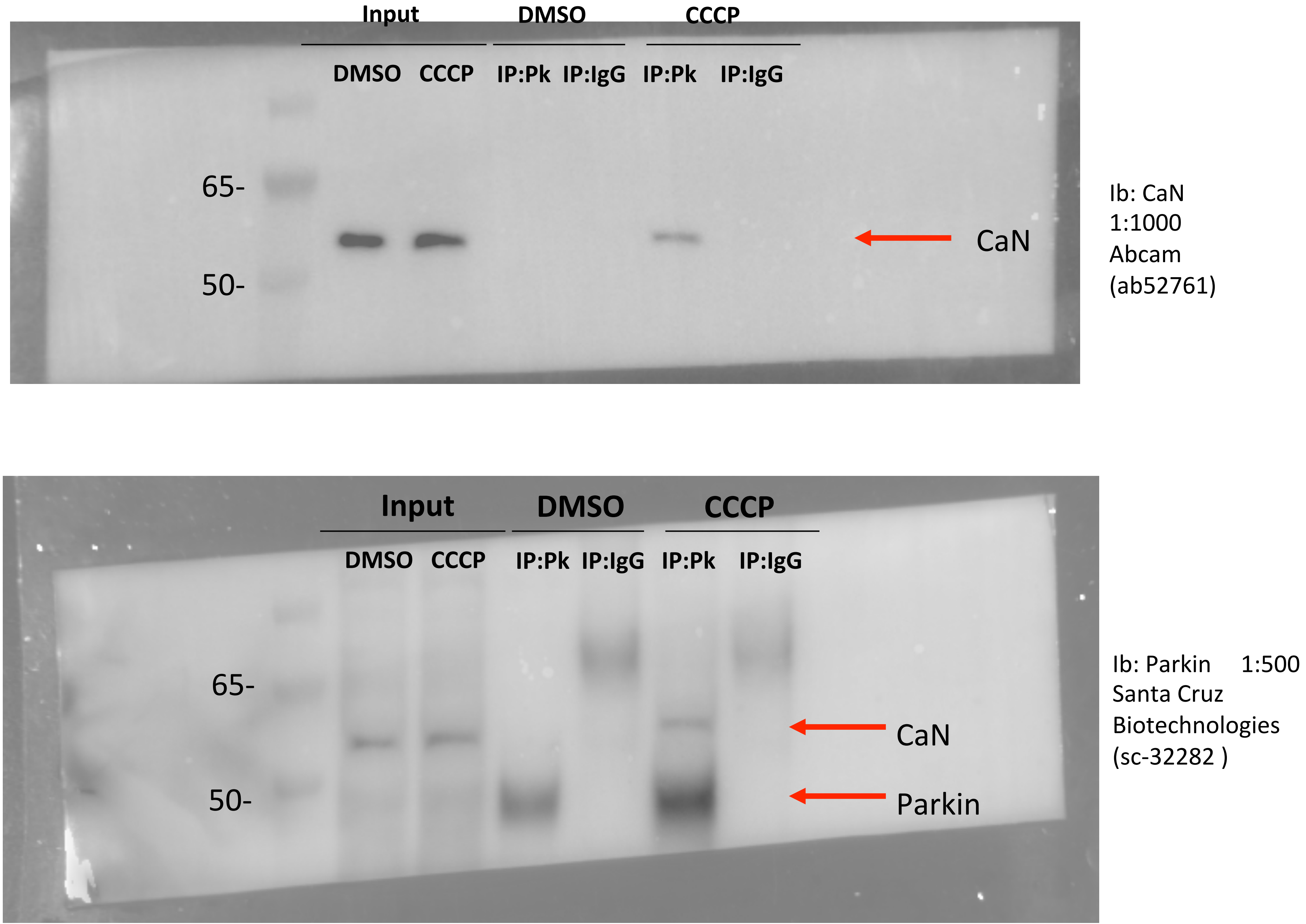
Input
DMSO
CCCP
DMSO
CCCP
IP:Pk
IP:IgG
IP:Pk
IP:IgG
65-
50-
CaN
Ib: CaN
1:1000
Abcam
(ab52761)
Input
DMSO
CCCP
DMSO
CCCP
IP:Pk
IP:IgG
IP:Pk
IP:IgG
65-
50-
CaN
Parkin
Ib: Parkin 1:500
Santa Cruz
Biotechnologies
(sc-32282)

FIGURE 7A

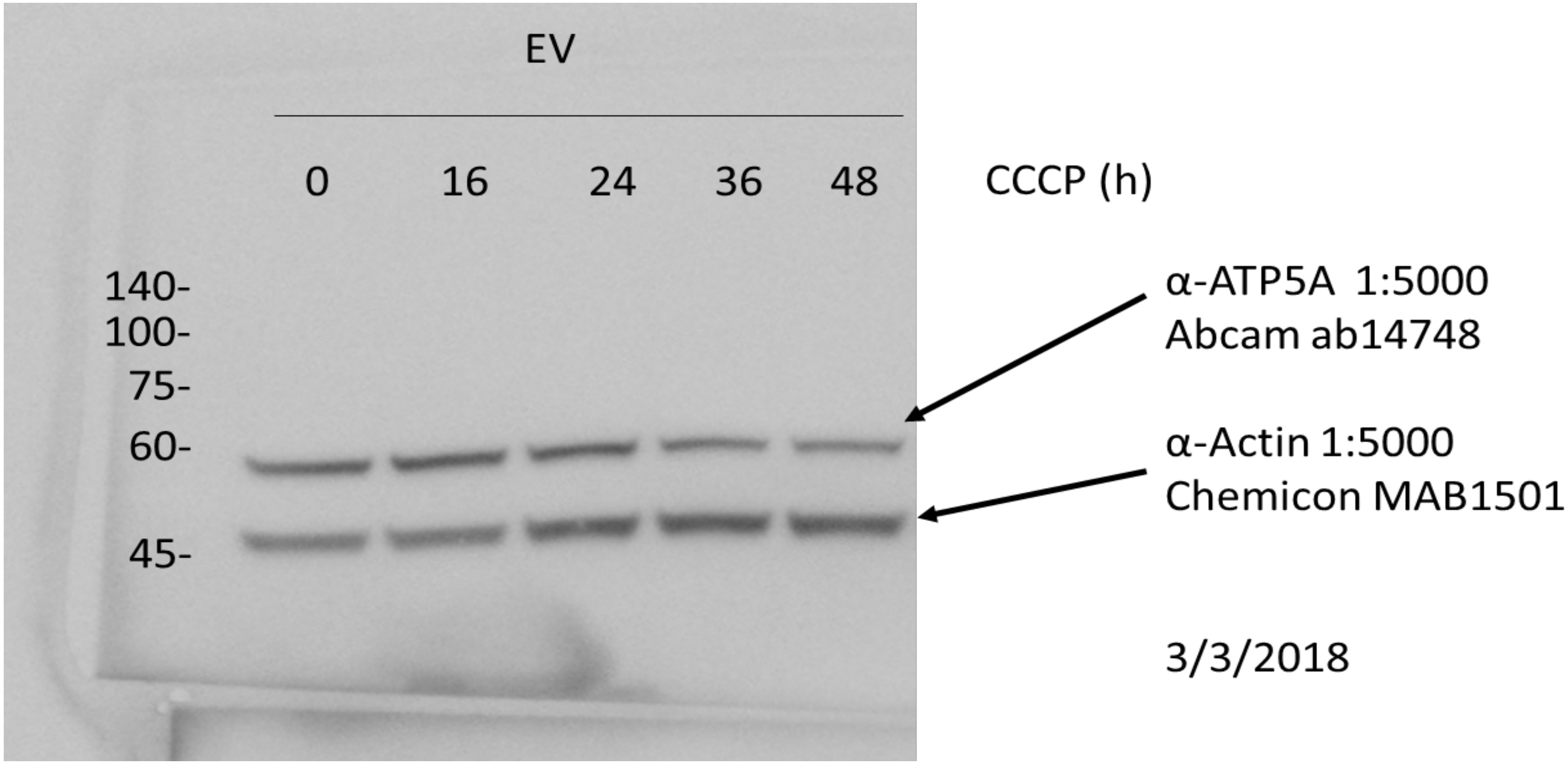

CCCP (h)

α-ATP5A 1:5000
Abcam ab14748

α-Actin 1:5000
Chemicon MAB1501

3/3/2018

FIGURE 7A

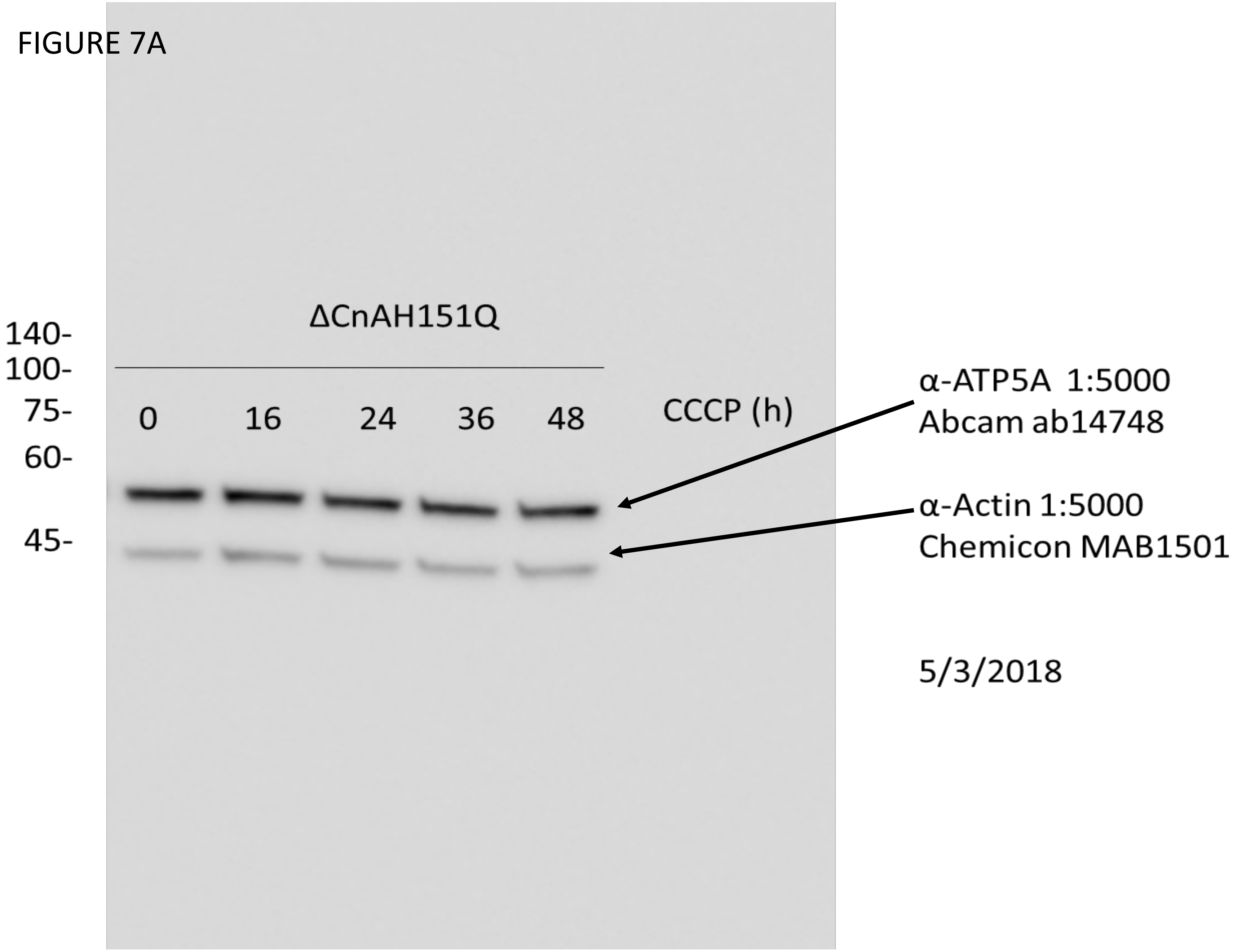
ΔCnAH151Q
140-
100-
75-
60-
45-
0
16
24
36
48
CCCP (h)
α-ATP5A 1:5000
Abcam ab14748
α-Actin 1:5000
Chemicon MAB1501
5/3/2018

FIGURE 7C

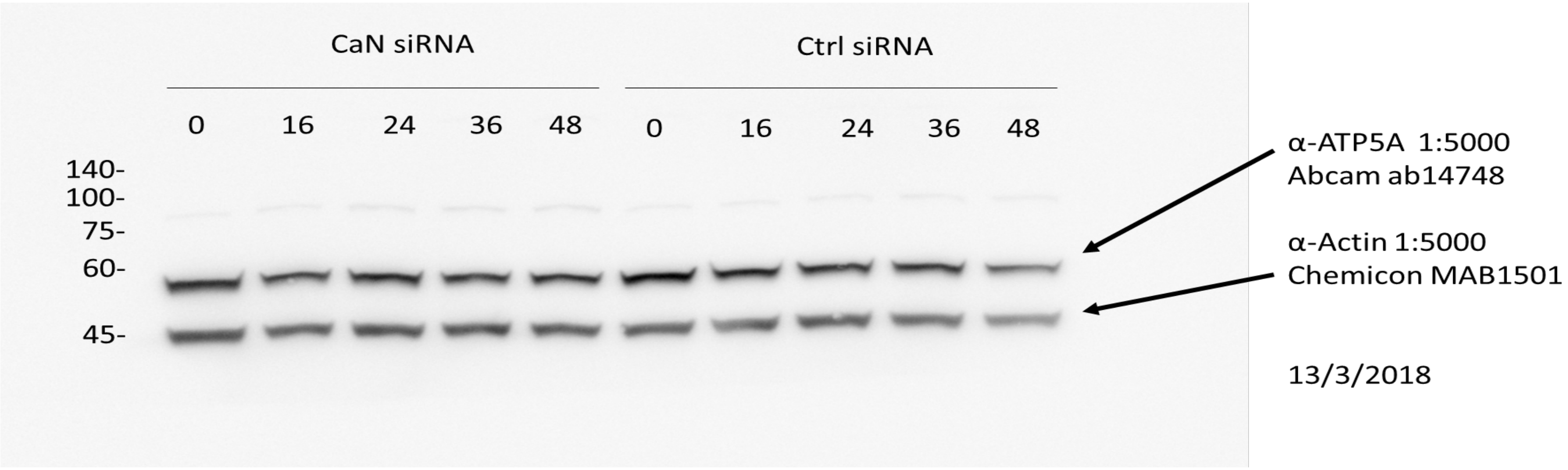
CaN siRNA
Ctrl siRNA
0
16
24
36
48
0
16
24
36
48
140-
100-
75-
60-
45-
α-ATP5A 1:5000
Abcam ab14748
α-Actin 1:5000
Chemicon MAB1501
13/3/2018

FIGURE 9A (left panel)-PINK1 WT

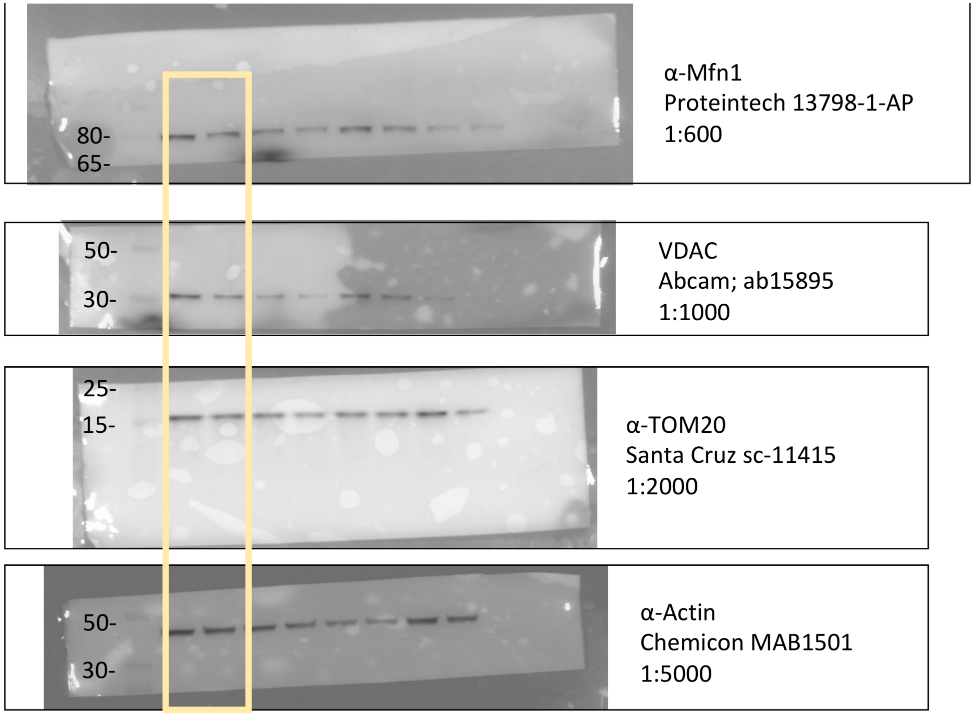

FIGURE 9A (right panel)-PINK KO

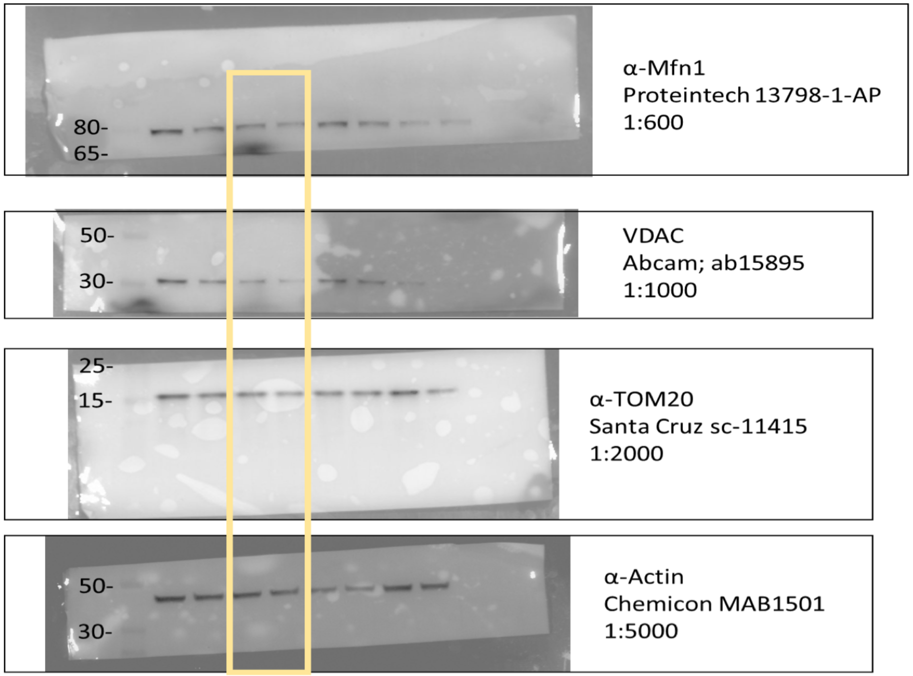

FIGURE 9F (panel on the left)-PINK1 WT

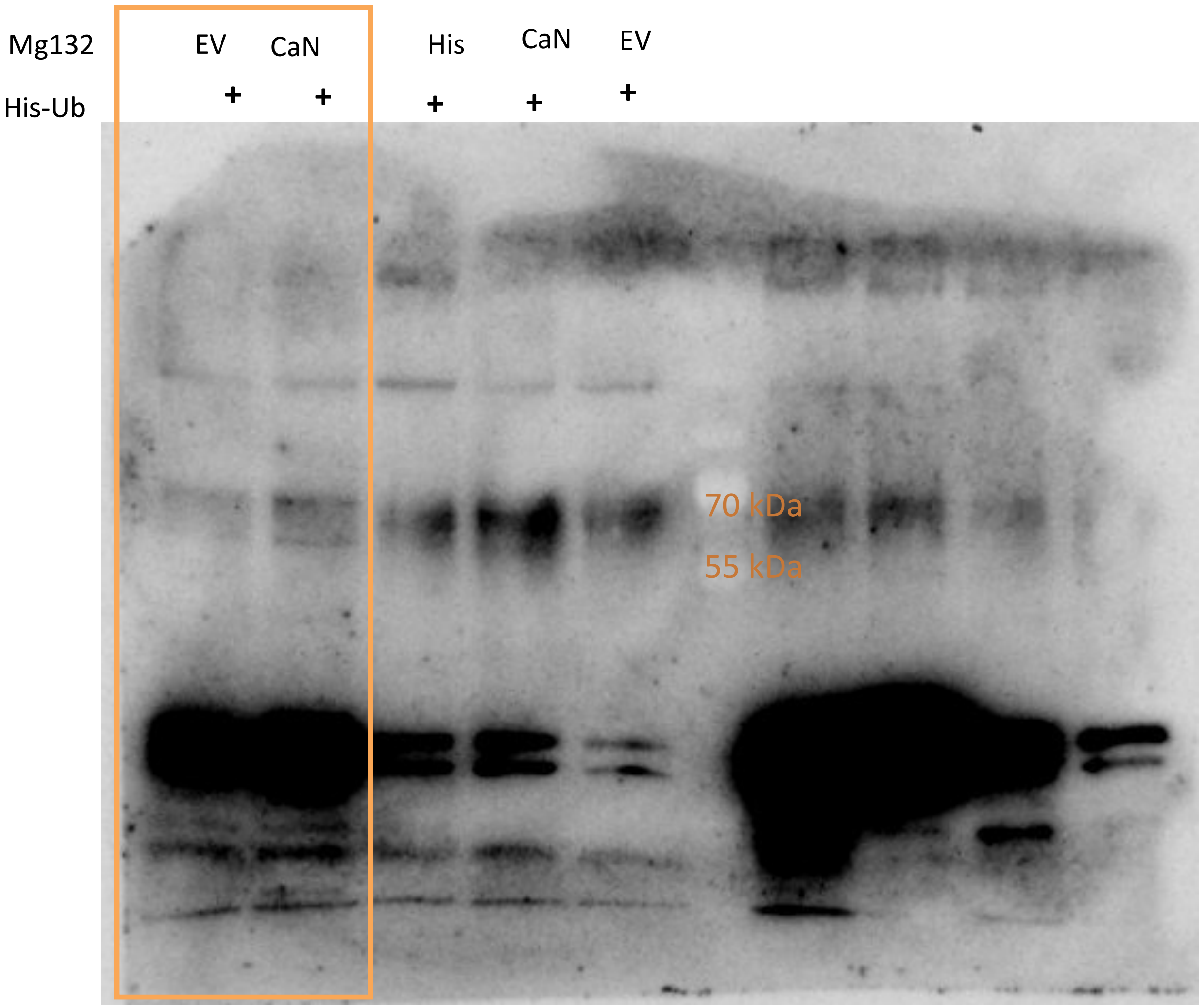

Ib: VDAC 1:1000
Abcam (Ab15895)

FIGURE 9F (panel on the left)-PINK1 WT

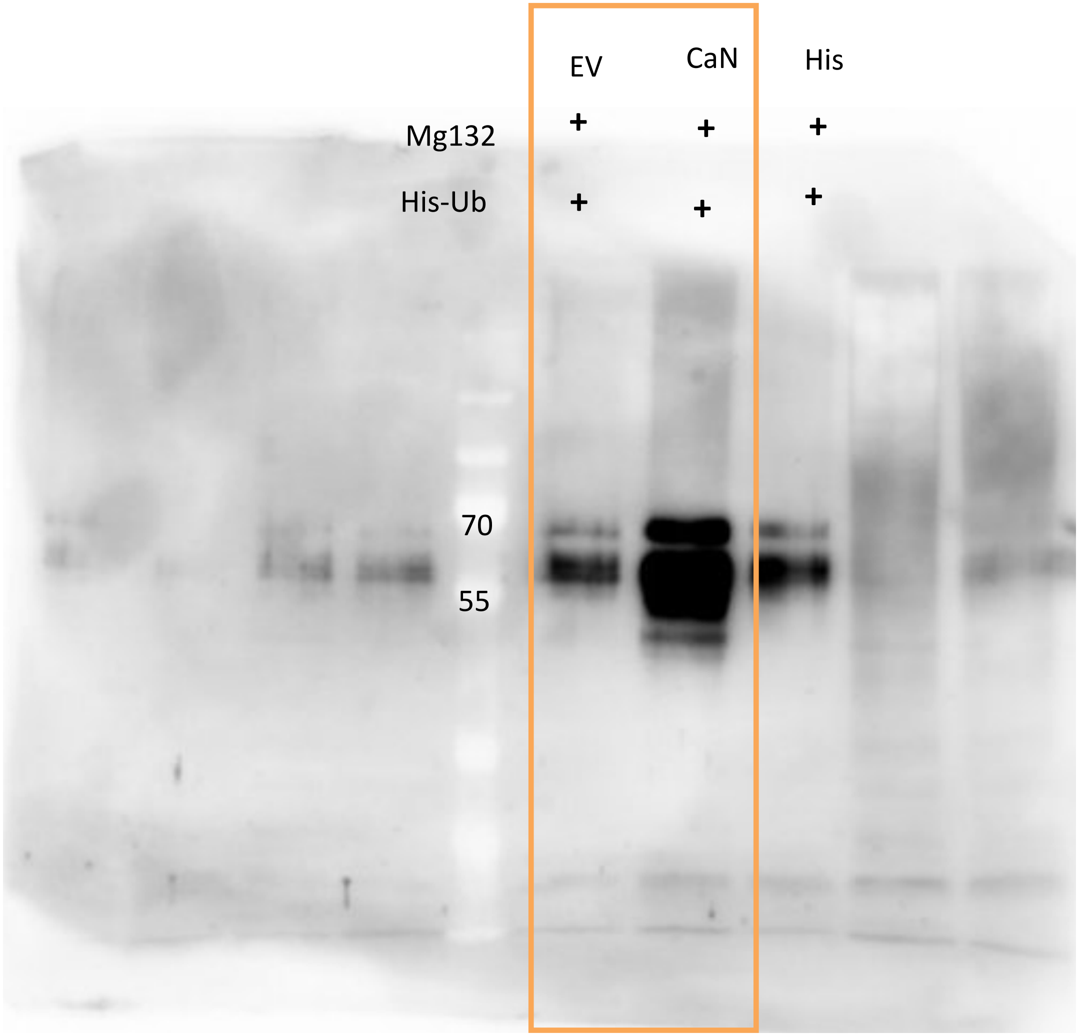

Ib: FLAG (Parkin) 3ug/ml
Sigma-Aldrich F3165

FIGURE 9F (panel on the right)-PINK1 KO

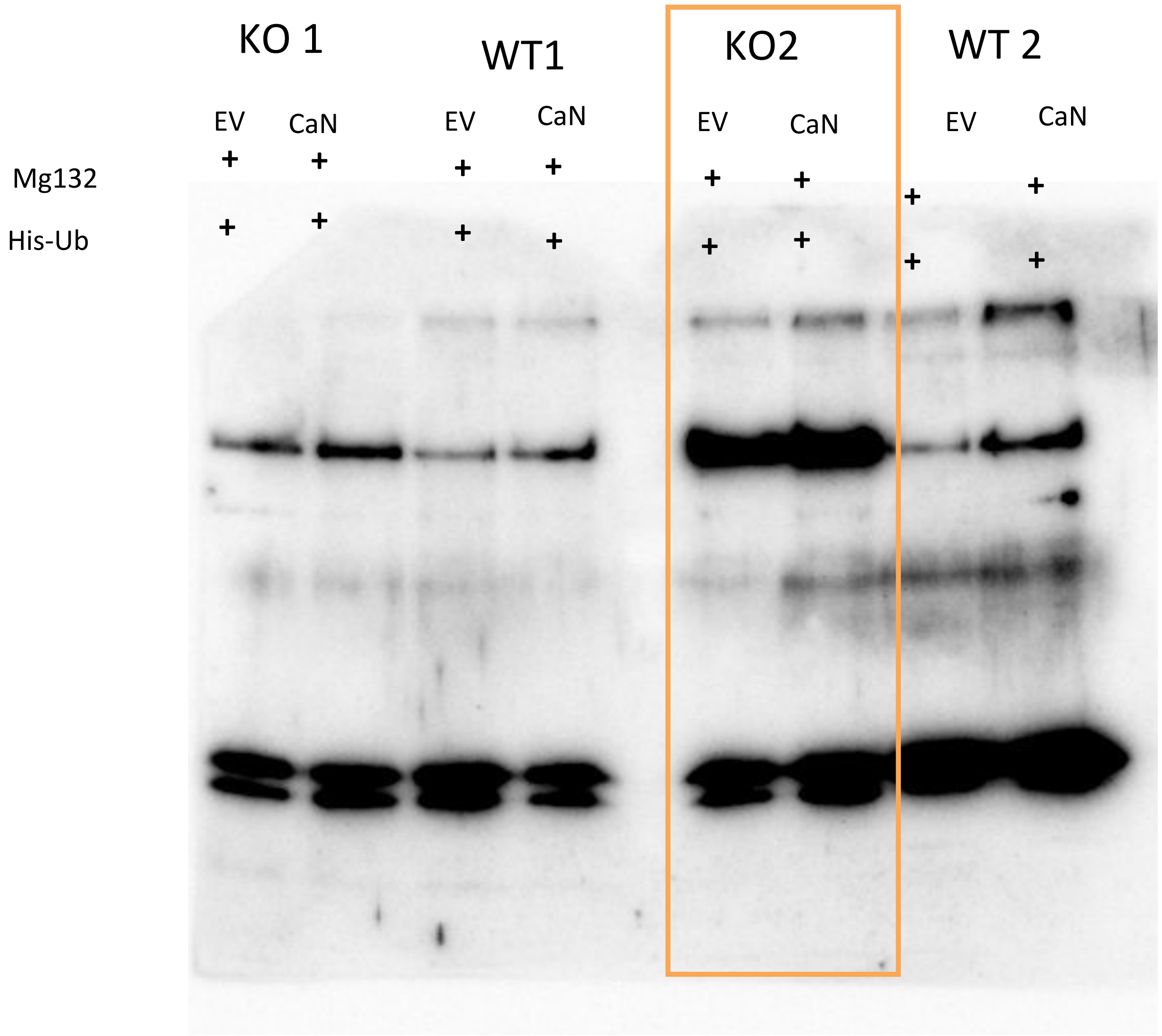

Ib: VDAC 1:1000
Abcam (Ab15895)

FIGURE 9F (panel on the right)-PINK1 KO

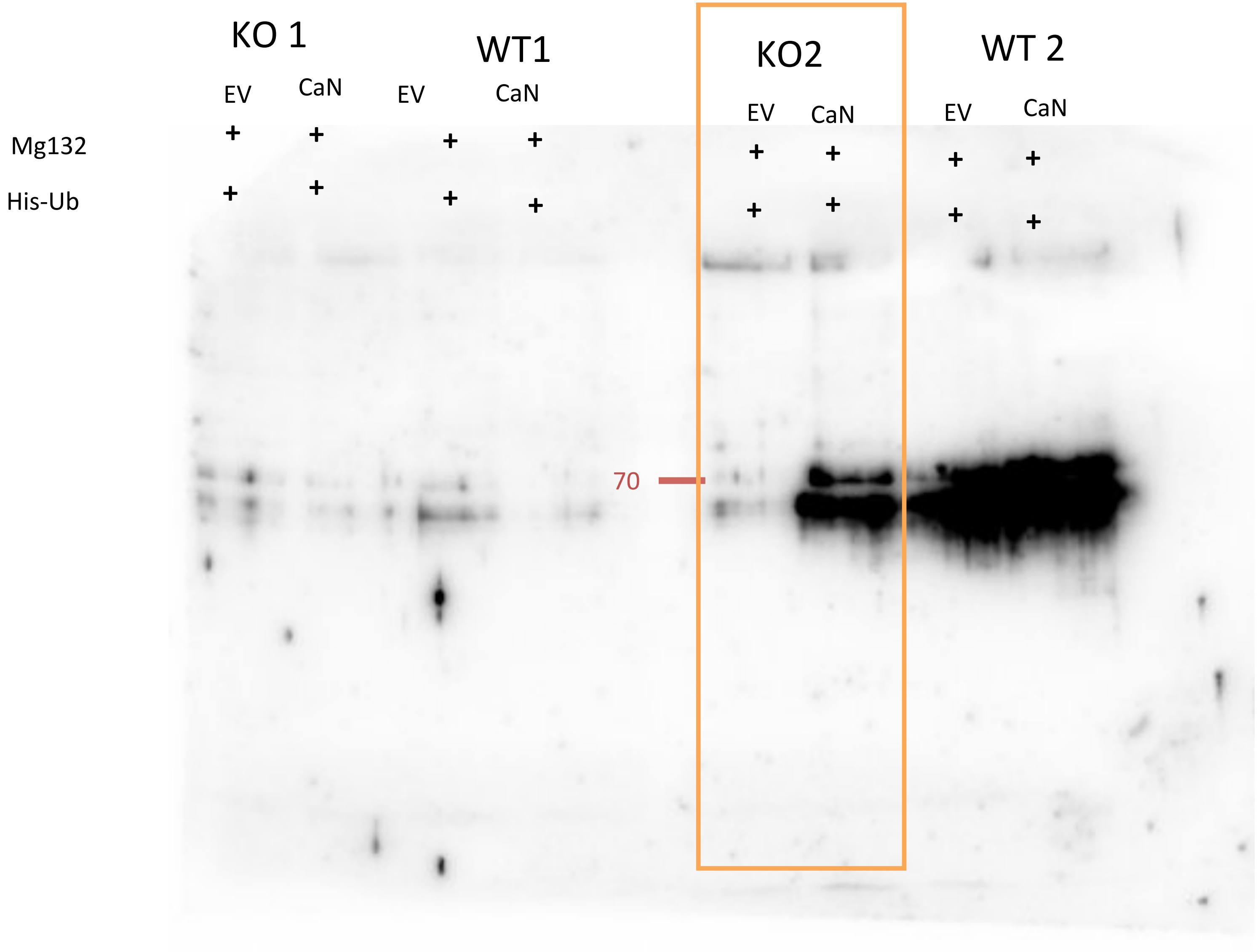

Ib: FLAG (Parkin) 3ug/ml
Sigma-Aldrich F3165

SUPPLEMENTARY FIGURE 4

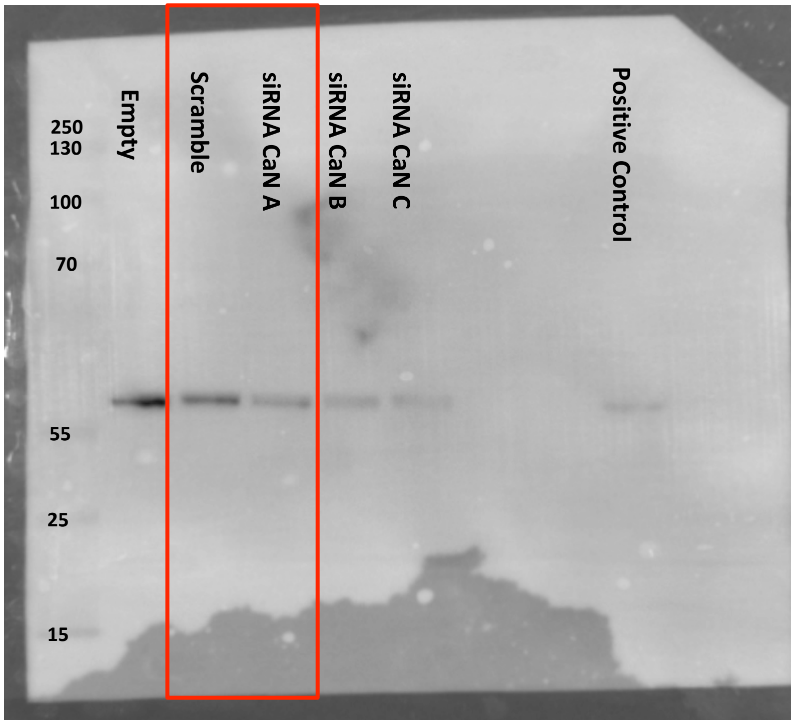

Ib: CaN 1:1000
Abcam (ab52761)

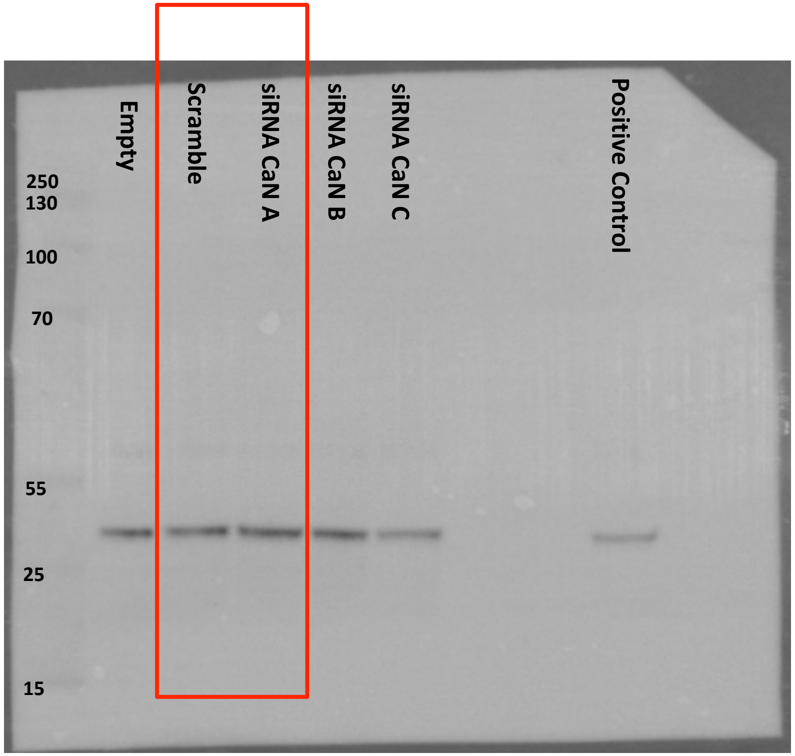

Ib: Actin 1:5000
Chemicon (MAB1501)

SUPPLEMENTARY FIGURE 6A

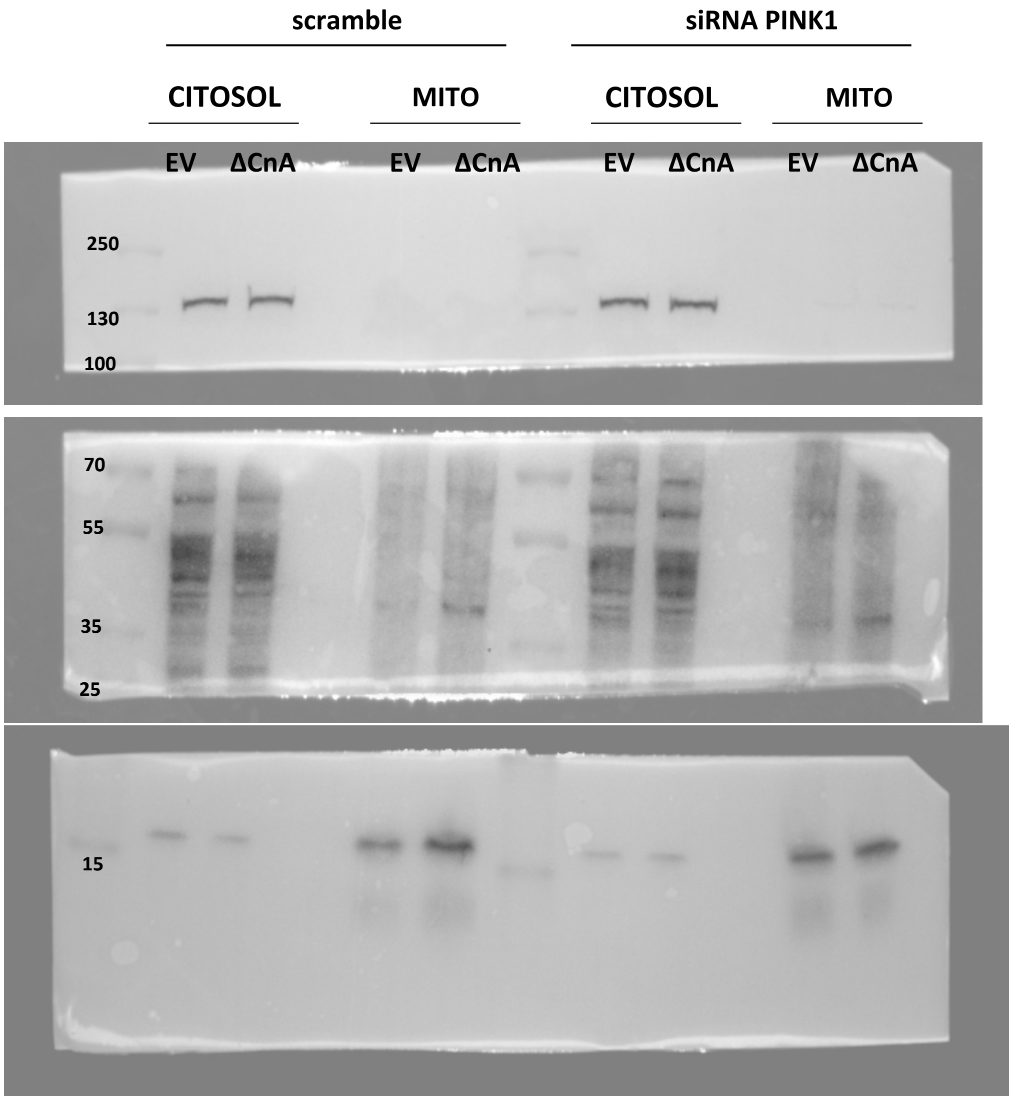

Ib: Vinculin 1:5000
Novus Biologicals (NB600-1293)

Ib: Parkin 1:500
Santa Cruz Biotechnologies (sc-32282)

Ib: TOM20 1:3000
Santa Cruz Biotechnologies (sc-11415)

SUPPLEMENTARY FIGURE 8A

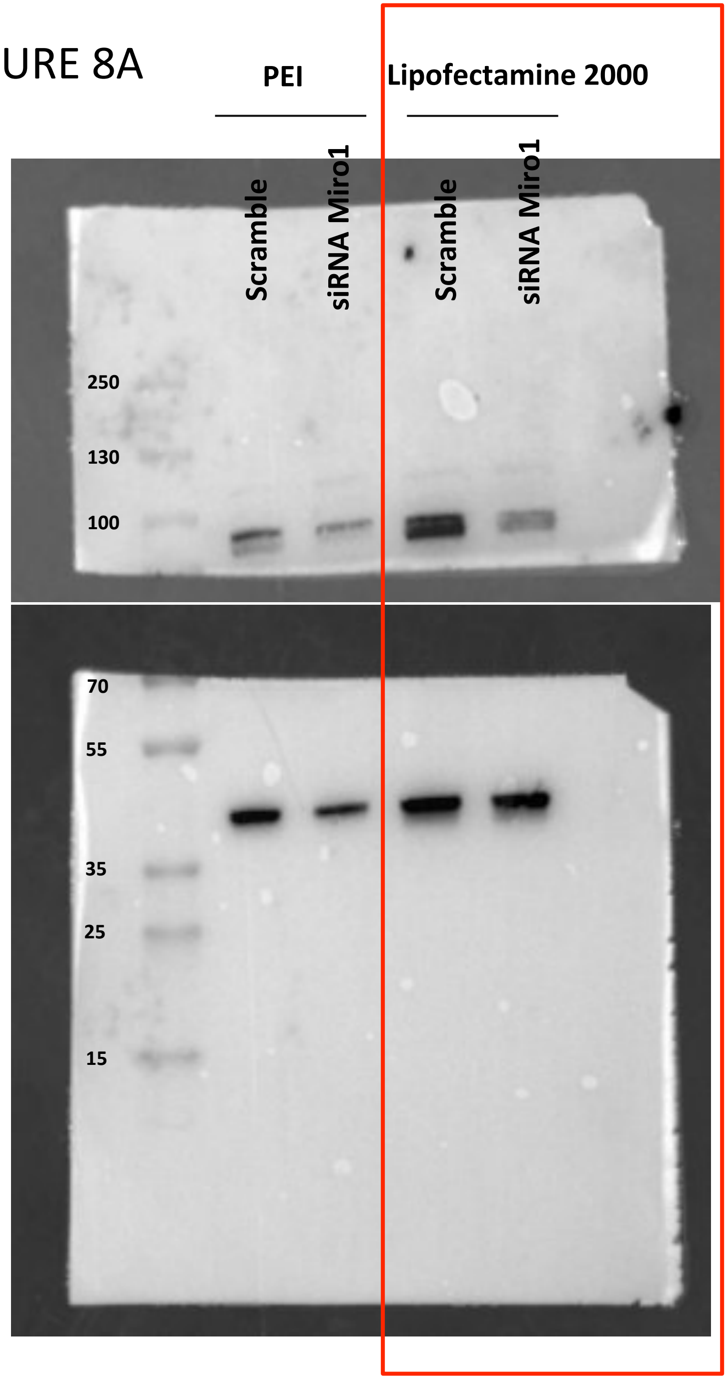

Ib: Miro1 1:1000
AB Clonal (A22551)

Ib: GAPDH 1:3000
Sigma Aldrich (G9545)

SUPPLEMENTARY FIGURE 10

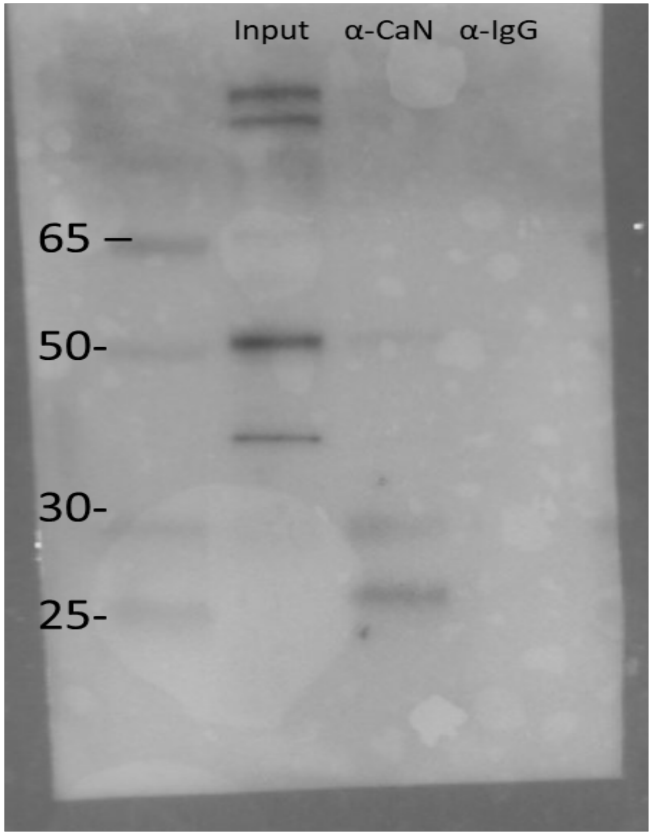

α-Parkin 1:1000
Abclonal A0968

11/03/2022

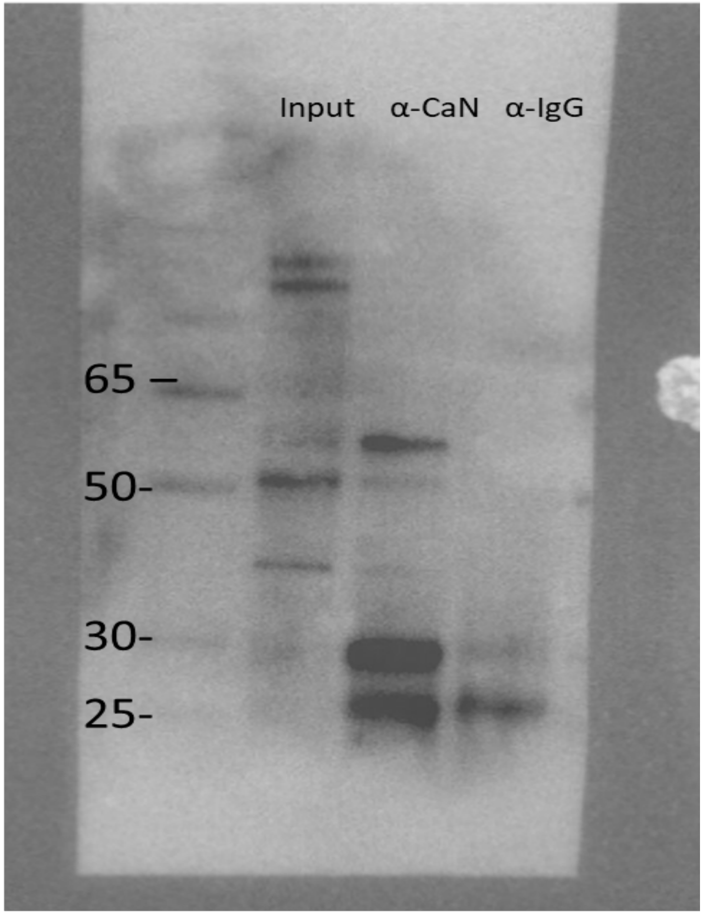

α- Calcineurin 1:1000
Abcam 52761

11/03/2022

SUPPLEMENTARY FIGURE 12A

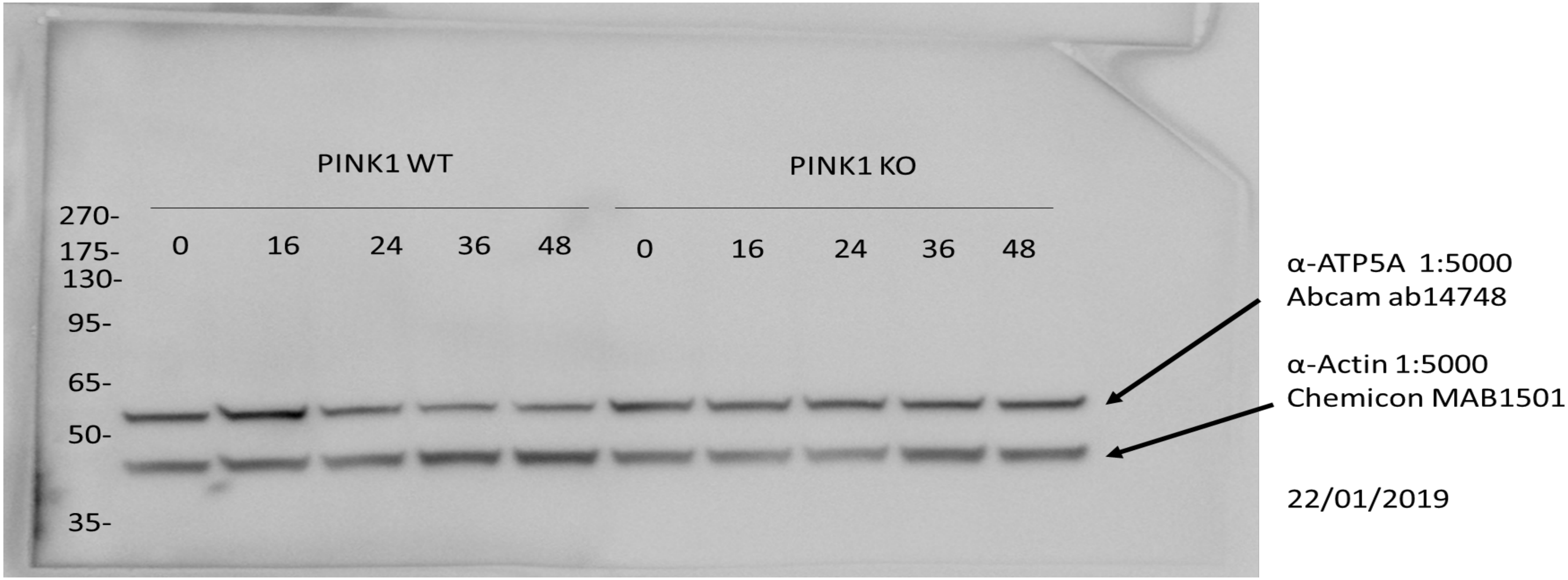

Supplement: Supplementary file 15 — Final Supplementary western blot uncrop [file 41418_2023_1251_MOESM15_ESM.pdf]
