# Supplementary material for: Activation of Ca2+ phosphatase Calcineurin regulates Parkin translocation to mitochondria and mitophagy in flies
Source: Cell Death Differ. 2024 Jan 18;31(2):217–38. doi: 10.1038/s41418-023-01251-9 (PMC10850161; doi:10.1038/s41418-023-01251-9)
Supplement: Supplementary file 17 — Related Manuscript File [file 41418_2023_1251_MOESM17_ESM.docx]

| **DIPARTIMENTO DI BIOLOGIA**  Via Ugo Bassi 58/b, 35131 Padova  Tel. +39 049 8276178  fax +39 049 8276181  [web:](http://www.biologia.unipd.it/) http://www.biologia.unipd.it  CF 80006480281  P.IVA 00742430283 | Dr Elena Ziviani  Associate Professor  University of Padova  Biology Department  Padova, Dec 5thOct 2022 |
| --- | --- |

Dear Professor Melino,

Please see in attachment our manuscript entitled “**Activation of Ca^2+^ phosphatase Calcineurin regulates Parkin translocation to mitochondria and mitophagy”.** We feel that our manuscript warrants consideration in CDD. In the following paragraphs we want to highlight the most important findings of our work, and the reasons why we think it deserve publication.

-Although a large number of Parkin substrates have been reported, the basis for Parkin substrate specificity is poorly understood. Moreover, **the mechanism underlying activation and translocation of Parkin onto mitochondria remains unclear,** because the presence of PINK1-dependent phosphorylation of Ubiquitin on impaired mitochondria **has been thought to be an indispensable prerequisite for Parkin activation and translocation**.

Our study challenges this view, and it shows that **Parkin translocation to mitochondria is PINK1 independent**, and it relays on the activation of Calcineurin.

Calcineurin (CaN) is a Ca^2+^-dependent phosphatase, **which can be activated upon physiological stimuli** such as those that are driven by Ca^2+^ oscillation. Independent works show that Parkin activation and mitophagy can also occur **in the absence of mitochondrial membrane potential depolarization (thus in the absence of PINK1 stabilization(Narendra *et al*, 2010))**, following proteotoxic stress(Burman *et al*, 2017; Jin & Youle, 2013) or **upon induction of mitochondrial Ca^2+^ oscillation**(Yu *et al*, 2021). These studies suggest that Parkin-dependent mitophagy can be activated upon physiological stimuli, which do not necessarily culminate in mitochondrial damage, and more importantly that **alternative PINK1-independent mechanisms of Parkin recruitment and activation can be predicted** (also supported by another study by Kubli DA., et al.(Kubli *et al*, 2015)).

Our study fully supports these works, and **it provides insights into the potential molecular mechanism underlying Parkin activation in the absence of PINK1,** that is via **CaN.**

-As highlighted by the reviewer, in our study there seem to be an **important difference** between Parkin-dependent mitophagy in PINK1 KO mouse embryonic fibroblasts (**MEFs**) as compared to **neuronal cells** of the PINK1 KO flies.

**In MEFs lacking PINK1**, CaN activation is sufficient to promote Parkin mitochondrial recruitment and activation, **but not mitophagy**. We explained this lack of effect by showing that in PINK1 KO MEFs expressing constitutive active CaN, **mitochondria do not fragment.** In particular, in these cells mitophagy is inhibited because **the fission machinery (Drp1 in particular) is not properly engaged.** As opposed to MEFs, **in neurons** of the fly heads, **PINK1 is dispensable for CaN-dependent mitophagy**. It is possible that **in neurons,** **mitophagic events predominately occur** **in a piece meal fashion,** through endosomal-lysosomal delivery and degradation of mitochondria derived vesicles (MDV)(McLelland *et al*, 2016; McLelland *et al*, 2014), **a process that does not require Drp1-dependent fission**(McLelland *et al.*, 2014). Neurons are **post-mitotic cells** with a complex architecture and a very much-developed endosomal-lysosomal system that accounts for important neuronal physiological functions. These **long-lived cells** might favour the MDV pathway over mitophagy to spare the entire organelle from degradation.

Another possibility is that **cell non-autonomous mechanisms** yet to be identified, which only occur in the complexity of the whole organism, may contribute to mitochondrial autophagy and quality control in neurons.

**Indeed, investigation of mitochondrial autophagy and quality control in specific cell type and in the whole organism remain fertile ground for further debate and discovery, and our paper will stands out to urge these studies.**

- Ou *in vivo* results show that CaN activation is protective *in vivo* in an animal model of neurodegeneration and mitochondrial dysfunction. Due to its diversified and crucial biological role and its abundant expression, particularly in the nervous system, CaN is suggested to play an important role for neuronal functions, and it is therefore an interesting target for neurodegenerative diseases. It is currently unclear whether **CaN serves to protect neurons during neurological disorders** or whether it boosts neuronal damage. **Our *in vivo* data clearly indicates that expression of constitutive active CaN is protective** in PINK1 KO flies, and that the protective effect is at least in part due to the mitophagic effect perpetuated by CaN activation.

Although the signalling cascade linking CaN to Parkin activation and mitophagy is not completely delineated, **our work warrants deeper investigation on the potential therapeutic opportunities of manipulating CaN expression in neurodegenerative conditions**.

Conclusively, we believe our work will set important milestones in the investigation of mitochondrial autophagy because (i) it provides insights into the molecular mechanism underlying PINK1-independent Parkin translocation and activation; (ii) it supports the importance of investigating mitochondrial autophagy and quality control in different cells types, and in whole organism; and (iii) it brings CaN upfront as therapeutic avenue to enhance quality control for counteracting neurodegenerative diseases.

With my best regards

Elena Ziviani

**References**

Burman JL, Pickles S, Wang C, Sekine S, Vargas JNS, Zhang Z, Youle AM, Nezich CL, Wu X, Hammer JA *et al* (2017) Mitochondrial fission facilitates the selective mitophagy of protein aggregates. *J Cell Biol* 216: 3231-3247

Jin SM, Youle RJ (2013) The accumulation of misfolded proteins in the mitochondrial matrix is sensed by PINK1 to induce PARK2/Parkin-mediated mitophagy of polarized mitochondria. *Autophagy* 9: 1750-1757

Kubli DA, Cortez MQ, Moyzis AG, Najor RH, Lee Y, Gustafsson AB (2015) PINK1 Is Dispensable for Mitochondrial Recruitment of Parkin and Activation of Mitophagy in Cardiac Myocytes. *PLoS One* 10: e0130707

McLelland GL, Lee SA, McBride HM, Fon EA (2016) Syntaxin-17 delivers PINK1/parkin-dependent mitochondrial vesicles to the endolysosomal system. *J Cell Biol* 214: 275-291

McLelland GL, Soubannier V, Chen CX, McBride HM, Fon EA (2014) Parkin and PINK1 function in a vesicular trafficking pathway regulating mitochondrial quality control. *EMBO J* 33: 282-295

Narendra DP, Jin SM, Tanaka A, Suen DF, Gautier CA, Shen J, Cookson MR, Youle RJ (2010) PINK1 is selectively stabilized on impaired mitochondria to activate Parkin. *PLoS Biol* 8: e1000298

Yu Z, Wang H, Tang W, Wang S, Tian X, Zhu Y, He H (2021) Mitochondrial Ca(2+) oscillation induces mitophagy initiation through the PINK1-Parkin pathway. *Cell Death Dis* 12: 632
